# Synthesis of Hybrid Cyclopeptides through Enzymatic Macrocyclization

**DOI:** 10.1002/open.201600134

**Published:** 2016-12-13

**Authors:** Emilia Oueis, Brunello Nardone, Marcel Jaspars, Nicholas J. Westwood, James H. Naismith

**Affiliations:** ^1^ School of ChemsitryUniversity of St AndrewsBSRC, North HaughSt AndrewsKY16 9STUK; ^2^ State Key Laboratory of BiotherapySichuan UniversityP. R. China; ^3^ Marine Biodiscovery CentreDepartment of ChemistryUniversity of AberdeenOld AberdeenAB24 3UEUK

**Keywords:** biotransformation, cyanobactin, hybrid macrocycles, macrocyclization, peptides

## Abstract

Natural products comprise a diverse array of molecules, many of which are biologically active. Most natural products are derived from combinations of polyketides, peptides, sugars, and fatty‐acid building blocks. Peptidic macrocycles have attracted attention as potential therapeutics possessing cell permeability, stability, and easy‐to‐control variability. Here, we show that enzymes from the patellamide biosynthetic pathway can be harnessed to make macrocycles that are hybrids of amino acids and a variety of manmade chemical building blocks, including aryl rings, polyethers, and alkyl chains. We have made macrocycles with only three amino acids, one of which can be converted to a thiazoline or a thiazole ring. We report the synthesis of 18 peptide hybrid macrocycles, nine of which have been isolated and fully characterized.

Natural products, in particular secondary metabolites, derived from various natural resources such as bacteria, fungi, marine sources, and plants, have been used as medicines since ancient times. Today they have utility directly as drugs, for example paclitaxel, or as active scaffolds, for example the β‐lactam ring.^1^ The advent of high‐throughput combinatorial chemistry has coincided with a decline in attention towards natural products by pharmaceutical companies.^2^ Recent progress in phenotypic screening and metabolomic technologies has rekindled interest in natural products.1a, 2, 3 In addition to vast structural and chemical diversity, natural products are often stereochemically complex; this feature has allowed them to evolve both specificity and potency.^4^ Macrocycles, in particular, whether peptidic or polyketides, are especially appealing, owing to their inherent chemical stability and structural rigidity,^5^ the later property reducing the entropic penalty for binding, thus raising affinity.

Several natural macrocycles possess useful biological and medicinal activities.^6^ Hybrid peptide macrocycles show increased chemical and structural diversity, for example, largazole (PKS/NRPS hybrid), zizyphines (cyclopeptide alkaloids), and maytansin (macrolide)6c, 7 (Figure 1). A drawback of peptidic macrocycles is their polarity, both in the side chain and the backbone, where it is an inescapable consequence of the peptide bond. High polarity hinders transport across lipid bilayers;^8^ strategies such as methylation, prenylation, and heterocyclization aim to overcome these problems.^9^ The synthesis of macrocyclic compounds where the backbone is a hybrid of both peptidic and non‐peptidic scaffolds is desirable, as it permits diversification and exquisite control of the physicochemical properties.^10^

**Figure 1:**
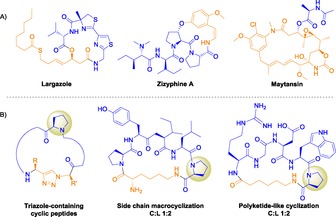
Structures of hybrid molecules. A) Natural hybrid macrocyclic compounds.6c, 7 B) Hybrid cyclopeptides previously synthesized using PatGmac.^11^ C:L indicates the ratio of the enzymatic macrocyclization product to the linear cleavage product.

PatGmac, the macrocyclization domain of PatG from the biosynthetic pathway of patellamides,^12^ recognizes octapeptides containing a thiazoline ring at the C‐terminal adopting a *cis* conformation followed by the recognition sequence AYD(GE).12b PatGmac is as a versatile tool capable of tolerating a range of different precursor peptides;^11^, ^13^ a proline can replace the thiazoline ring,^11^, 13a the amino acids of the core peptide are variable almost at will,^11^, ^13^ PatGmac has been reported to make macrocycles of up to 22 amino acids,13b and can tolerate non‐natural amino acids and triazole amide bond mimics.11a, 13a In an elegant paper by Schmidt and co‐workers,11b the chemistry of the *N*‐terminus of the core peptide was extensively evaluated to study the cyclization patterns of lysine and its analogues. Depending on the length of the sequence and the amino acid side chain, both backbone and side‐chain cyclization were observed along with a linear product. The synthetic utility of PatGmac derives precisely from its promiscuity towards the core sequence. The cyclic dehydratases, PatD and LynD, which make thiazoline and oxazoline rings from cysteine and serine (threonine), respectively, have also been shown to tolerate the presence of non‐natural amino acids,13a but it is not known how they tolerate non‐amino acid groups. Here, we have used enzymes from cyanobactin biosynthesis to generate eighteen hybrid peptidic/non‐peptidic macrocycles that explore a wide range of chemical variation. Nine of these macrocycles (**1**–**6**,**7 a–c**, Figure 2) were made at sufficient scale to allow full characterization.

**Figure 2:**
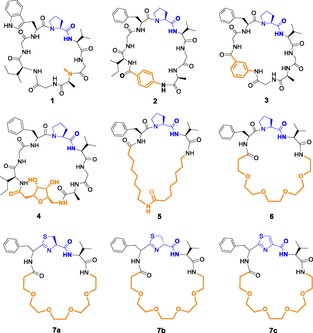
Structures of fully characterized macrocycles **1**–**6**, **7 a–c**.

Nineteen analogues (**8**–**26**) were synthesized based on a common precursor peptide VGAGIGFP, in which one or more amino acids were substituted with non‐natural, non‐amino‐acid scaffolds. All but one of the substrates finished with a Pro in the core peptide (one had a Cys) and all possessed a C‐terminal AYD, the minimal recognition sequence for PatGmac, or AYD‐Doc (8‐amino‐3,6‐dioxaoctanoic acid; Table 1). Peptides **8**–**10** contain a one‐residue substitution involving small non‐natural amino acids β‐Ala, GABA, and Doc (8‐amino‐3,6‐dioxaoctanoic acid), respectively. Peptide **11** has an *N‐*Me amino acid. Peptides **12**–**18** contain either an isomer of amino benzoic acid (Abz) at different positions or a ribose‐derived sugar amino acid (Rib). Finally, peptides **19**–**26** represent the hybrid peptides, where a polyketide chain 7‐aminoheptanoic acid (7Ahp), 8‐aminooctanoic acid (8Aoc), or a polyether amino acid (PEG)_4_ is introduced at different positions of the peptide. Peptide **26** has a Cys, which requires a heterocyclization reaction prior to macrocylization. The peptides were all synthesized through solid‐phase peptide synthesis by using the Fmoc strategy and commercially available reagents (see the Supporting Information). The fully protected Fmoc–sugar amino acid **27** was synthesized in a similar approach to the one reported in the literature^14^ for the synthesis of the Boc‐protected analogue starting from *d*‐ribose. Minor changes, such as the use of benzyl ester protection in the synthesis, were required to produce the desired compound **27** in good yields (see Scheme 1 and the Supporting Information). The azide of compound **28** was selectively reduced under the Staudinger reaction conditions to afford the corresponding amine, which was immediately Fmoc protected to yield **29**. Benzyl deprotection^15^ gave **27** in 62 % yield. Compound **27** was used for peptide synthesis, and deprotection of the alcohols was accomplished during the final acidic cleavage of the peptide.

**Table 1:** Synthetic hybrid precursor peptides and their corresponding PatGmac macrocyclization products.

Peptide	Sequence^[a]^	Product^[b]^	Macrocycle^[c]^
**8**	VGA‐**β‐Ala**‐IGWP**AYD**‐Doc	C	
**9**	VGA‐**GABA**‐IGWP**AYD**‐Doc	C	
**10**	VGA‐**Doc**‐IGWP**AYD**‐Doc	C	
**11**	VG(***N*** **‐Me**)AGIGWP**AYD**‐Doc	C	**1**
**12**	VGA‐**2‐Abz**‐IGFP**AYD**	C	
**13**	VGA‐**3‐Abz**‐IGFP**AYD**	C	
**14**	VGA‐**4‐Abz**‐IGFP**AYD**	C	**2**
**15**	VGAG‐**2‐Abz**‐GFP**AYD**	C	
**16**	VGAG‐**3‐Abz**‐GFP**AYD**	C	**3**
**17**	VGAG‐**4‐Abz**‐GFP**AYD**	C	
**18**	VGA‐**Rib**‐IGFP**AYD**	C	**4**
**19**	**8Aoc**‐AGIGFP**AYD**	L	
**20**	V‐**7Ahp**‐GAGFP**AYD**	C	
**21**	VGAG‐**7Ahp**‐P**AYD**	NR	
**22**	VGAG‐**8Aoc**‐FP**AYD**	C	
**23**	V‐**8Aoc**‐FP**AYD**	L	
**24**	V‐**8Aoc‐8Aoc**‐FP**AYD**	C	**5**
**25**	V‐**(PEG)_4_**‐FP**AYD**	C	**6**
**26**	V‐**(PEG)_4_**‐FC**AYD**	C	**7**

[a] Highlighted is the minimal recognition sequence where the peptide is cleaved. Non‐natural scaffolds in the sequence are in bold. [b] All successful enzymatic reactions afforded exclusively either cyclic or linear products, as detailed. Doc=8‐amino‐3,6‐dioxaoctanoic acid; GABA=γ‐aminobutyric acid; Abz=aminobenzoic acid; Rib=ribose sugar amino acid; 8Aoc=8‐aminooctanoic acid; 7Ahp=7‐aminoheptanoic acid, PEG=polyethylene glycol; C=macrocyclic; L=linear; NR=no reaction. [c] The corresponding isolated macrocycle number.

**Scheme 1:**
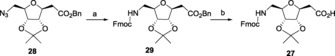
Synthesis of ribose sugar amino acid derivative **27**. Reagents and conditions: a)  Ph_3_P, THF/H_2_O, rt, O/N (i); FmocOSu, DIEA, CH_2_Cl_2_, rt, O/N, 56 % (ii); b) Et_3_SiH, Pd/C, MeOH, rt, 10 min, 60 %.

Substrates **12**–**26** were purified before reaction with the enzyme(s), and substrates **8**–**11** were used without any further purification after cleavage from the resin.

The macrocylization reactions were set up on small scale (<1 mL reactions) with PatGmac in bicine buffer at pH 8.1 and 37 °C. The reactions were followed by MALDI‐MS to monitor progress. MS–MS fragmentation data established the structure of the different products with the presence of the PV dipeptide fragment being indicative of macrocycle formation. Table 1 summarizes the results of the reactions; in brief, only peptide **21** gave no reaction, whereas **20** and **23** yielded a linear cleaved product. The reaction rate for these transformations was still slow, with reaction times ranging between 2–4 weeks. Nine representative cyclic hybrid peptides (**1**–**6**, **7 a–c**; Figure 2), products of peptides **11**, **14**, **16**, **18**, **24**, **25**, and **26**, were purified from a large‐scale reaction and fully characterized by using NMR spectroscopy. One of the products of peptide **26** (**7 a**) was subsequently oxidized.

Substrates with β‐Ala and GABA (**8**, **9**) processed essentially as native peptides, perhaps unsurprisingly because these are very similar to amino acids. More interestingly, a longer chain in that position, namely Doc (**10**), also gave only the macrocycle. The inclusion of a *N‐*methylated amino acid in peptide **11** proceeded to afford the corresponding macrocycle **1**. The incorporation of a rigid aromatic scaffold, 2‐, 3‐, or 4‐aminobenzoic acid at either position 4 or 5 of the core peptide (**12**–**17**), proceeded normally. Remarkably, the presence of a sugar amino acid (Rib, **18**), known to cause conformational restraints in peptidic analogues,^16^ afforded exclusively the macrocyclic product **4**.

Schmidt and co‐workers have previously shown the tolerance of PatGmac for polyketide‐like scaffolds such as alkyl chains as part of the core peptide.11b Peptide **19**, having a *N*‐terminal 8Aoc unit, gave only linear peptide, whereas **20**, which has a *N*‐terminal Val preceding the alkyl chain of 7Ahp, yielded exclusively the cyclic product. Comparing substrates **19** and **20** indicated that an amino acid at the *N*‐terminus was important with our sequence for efficient macrocyclization (Table 1). Schmidt and co‐workers,11b showed both cyclic and linear products were obtained with an *N*‐terminal amino‐alkyl chain of a different peptidic sequence (Figure 1). Peptide **21** with the 7Ahp chain immediately before the required Pro in the core peptide failed to react, but when a Phe was introduced in between 8Aoc and Pro (**22**), the macrocycle was obtained. It seems that an additional amino acid is required *N*‐adjacent to the terminal Pro or thiazoline of the core peptide for it to be processed by the enzyme (see Table 1 and the Supporting Information).

We observed a length requirement for the macrocyclization reaction. Compound **23**, which has an 8Aoc unit between a *N*‐terminal Val and the Phe–Pro at the C‐terminus, gave only a linear product, whereas **24** with two molecules of 8Aoc, yielded only macrocycle **5**. Substrate **25** was designed with a pegylated chain (PEG)_4_ and yielded the corresponding macrocycle **6**. Full conversion of substrates **25** and **26** was difficult to achieve, even after long incubation times (4 weeks, adding more enzyme), reaching only to about 70 % conversion to macrocycle (the remainder was unreacted starting material). In **26**, the Pro was replaced with a Cys, which was converted to thiazoline with the engineered LynD enzyme^17^ (establishing this enzyme tolerated PEG) and subsequently macrocyclized to **7** (Scheme 2).

**Scheme 2:**
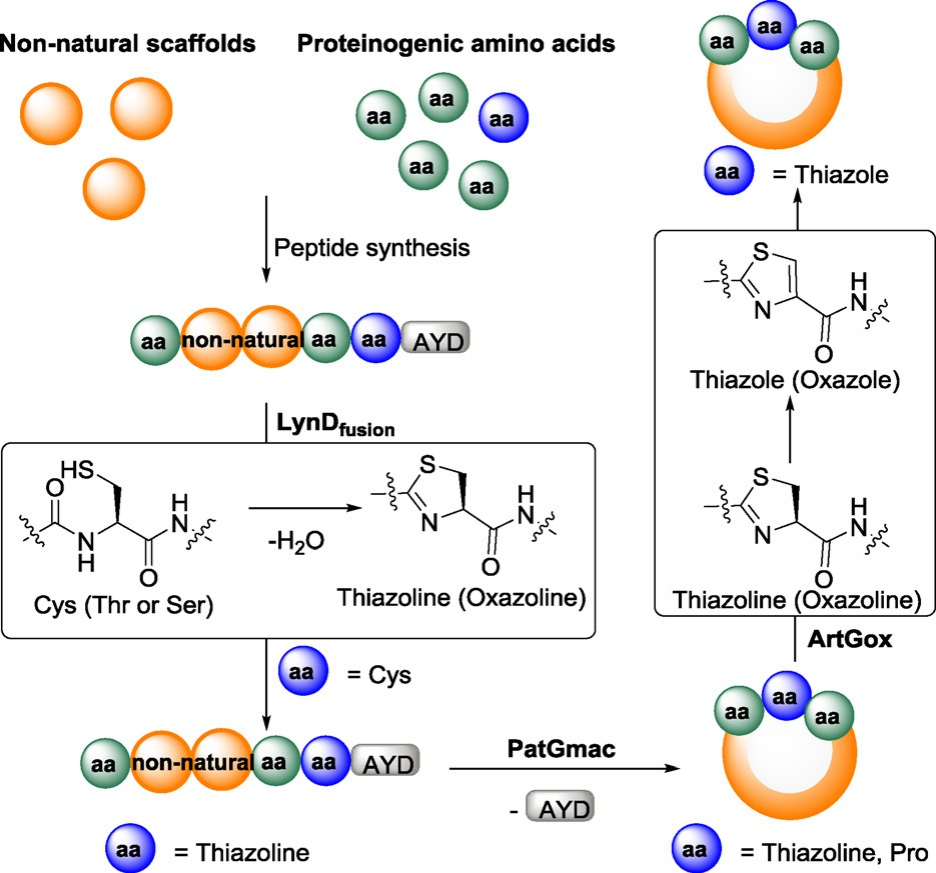
General reaction scheme of the enzymatic transformations.

Compounds **1** and **2** were present as a single conformer, whereas compounds **3**–**6** were all present as a mixture of two conformers in approximately 2:1 (**3**, **4**) or 3:2 (**5**, **6**) ratios, as confirmed by exchange spectroscopy (EXSY) experiments (see the Supporting Information). Compound **7**, containing the thiazoline ring, shows two separable peaks in the HPLC spectrum, in an 8:2 (**7 a**/**7 b**) ratio. Both compounds have identical HRMS and MS–MS fragmentation data. The NMR spectra of these two compounds are, nonetheless, different, but confirm the same peptidic sequence [cyclic V(PEG)_4_FThz]; however, an EXSY experiment showed no exchange. We postulate that **7 a** and **7 b** are epimers. As thiazoline with the natural (*R*) *l*‐configuration is a substrate for the oxidase enzyme,12d **7 a** and **7 b** were incubated in the presence of this enzyme overnight. **7 a** afforded compound **7 c**, which was isolated, purified, and characterized by NMR as the thiazole, confirming the oxidase enzyme also tolerates non‐peptide groups (Scheme 2). 7 **b** was, however, not enzymatically oxidized. We suggest the thiazoline stereocenter of **7 a** is (*R*) *l*, whereas it has inverted to a (*S*) *d*‐configuration in **7 b**. This has precedent; the thiazoline moiety of piperidine thiopeptides is known to be spontaneously epimerized from the (*R*) *l‐* to the (*S*) *d*‐configuration.^18^

Natural peptide hybrid macrocycles have interesting and useful biological properties,6c but are, of course, limited to natural building blocks. The routine synthesis of macrocycles composed of peptide and non‐peptide building blocks has wide potential in medicinal chemistry, allowing access to novel scaffolds and the fine‐tuning of properties. The patellamide biosynthetic pathway has enzymes that catalyze a number of valuable chemical reactions including heterocylization, oxidation, and macrocyclization. The enzymes in the pathway largely separate recognition from catalysis, and are thus assumed to have a high degree of promiscuity.12c We have used these enzymes to synthesize diverse macrocycles containing methylated amide, sugars, non‐natural amino acids, aromatic rings, thiazole, thiazoline, polyether moieties, and polyketide‐like chains. The overall yield of the enzymatic processes (starting from substrate) varied from 27 to 66 % (Table S2). More interestingly, the enzymes can synthesize a product with just three amino acids. Thus, we have established that these enzymes are tools that, when combined with chemical synthesis of substrates, can produce highly modified, complex, and diverse peptide/non‐peptide hybrid macrocycles.

## Supporting information

As a service to our authors and readers, this journal provides supporting information supplied by the authors. Such materials are peer reviewed and may be re‐organized for online delivery, but are not copy‐edited or typeset. Technical support issues arising from supporting information (other than missing files) should be addressed to the authors.

Supplementary
