# Supplementary material for: Synthesis of Hybrid Cyclopeptides through Enzymatic Macrocyclization
Source: ChemistryOpen. 2016 Dec 13;6(1):11–4. doi: 10.1002/open.201600134 (PMC5288752; doi:10.1002/open.201600134)
Supplement: Supplementary file 1 — Supplementary [file OPEN-6-11-s001.pdf]

ChemistryOPEN

Supporting Information

© 2017 The Authors. Published by Wiley-VCH Verlag GmbH & Co. KGaA, Weinheim

Synthesis of Hybrid Cyclopeptides through Enzymatic Macrocyclization

Emilia Oueis,[a] Brunello Nardone,[a] Marcel Jaspars,[c] Nicholas J. Westwood,*[a] and James H. Naismith*[a, b]

open_201600134_sm_miscellaneous_information.pdf

I. STRUCTURES OF THE NON NATURAL, NON AMINO ACID SCAFFOLDS 2

II. STRUCTURES OF THE SYNTHESIZED HYBRID CYCLIC PEPTIDES 2

III. STRUCTURES OF THE LINEARLY CLEAVED HYBRID PEPTIDES 4

IV. GENERAL INFORMATION AND MATERIALS 4

V. GENERAL PROCEDURES 7

PatGmac cloning, expression and purification 7

LynDfusion cloning, expression and purification 7

ArtGox cloning, expression and purification 7

Solid-phase peptide synthesis of peptides 8-26, 36-38 7

Pat Gmac macrocylization reaction of peptides 8-25, 36-38 8

One pot heterocyclization / macrocylization reaction of peptide 26 8

Enzymatic oxidation of thiazoline-containing macrocycle 7a-b 9

Synthesis of the sugar amino acid (Rib) 27 9

VI. MS AND HPLC DATA OF STARTING AND FINAL HYBRID PEPTIDES 16

VII. NMR DATA OF FINAL CYCLIC PEPTIDES 18

VIII. NMR SPECTRA OF FINAL CYCLIC PEPTIDES 28

IX. EXSY NMR OF FINAL CYCLIC PEPTIDES 37

X. NMR SPECTRA OF SUGAR ANALOGUES INTERMEDIATES 40

XI. MS-MS DATA OF ENZYMATIC REACTION PRODUCTS OF PEPTIDES 8-26 45

XII. MALDI-MS TRACES OF THE REACTIONS NOT PROCESSED BY PATGMAC WITH STARTING HYBRID PEPTIDES 21, 36-38 52

XIII. LC-MS TRACES OF FINAL CYCLIC PEPTIDES 56

XIV. LC TRACES AND MS SPECTRA OF THE CRUDE RESULTING PEPTIDES AFTER CLEAVAGE FROM THE RESIN OF PEPTIDES 8-11 65

XV. LC-MS TRACES OF STARTING HYBRID PEPTIDES 12-26, 36-38 69

XVI. LC-MS TRACES OF THE SUGAR ANALOGUES INTERMEDIATES 87

XVII. REFERENCES 89

I. Structures of the non natural, non amino acid scaffolds

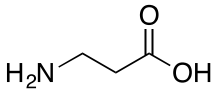

β-Ala

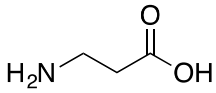

GABA

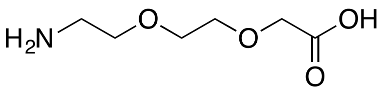

Doc

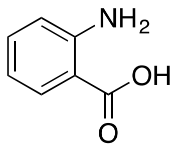

2-Abz

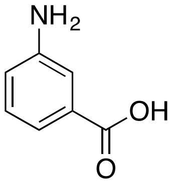

3-Abz

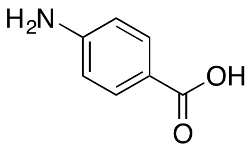

4-Abz

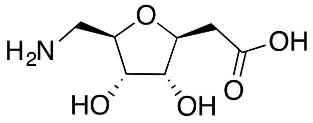

Rib

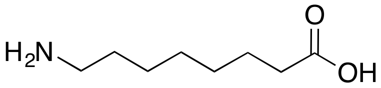

8Aoc

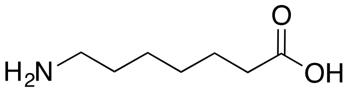

7Ahp

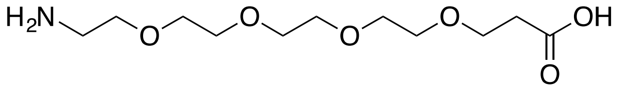

$(PEG)_4$

II. Structures of the synthesized hybrid cyclic peptides

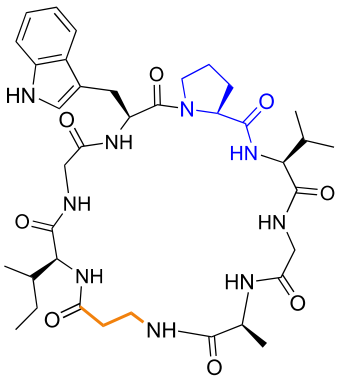

Cyclo(-VGA-β-Ala-IGWP-)

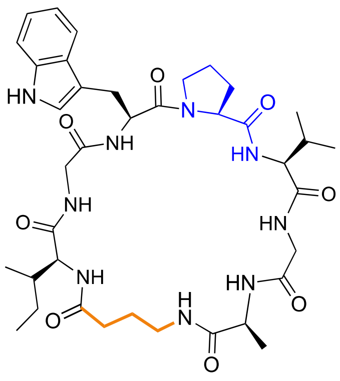

Cyclo(-VGA-GABA-IGWP-)

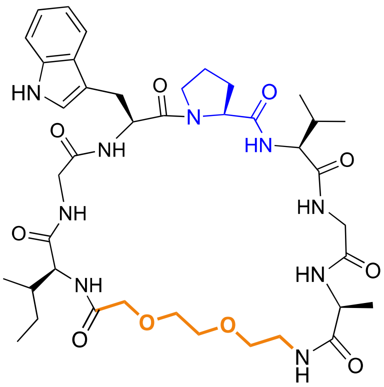

Cyclo(-VGA-Doc-IGWP-)

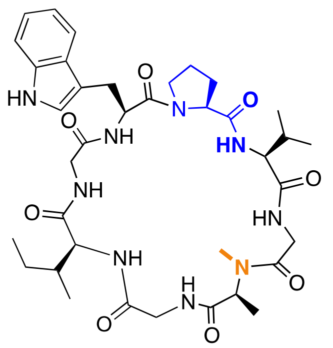

(1) Cyclo(-VG(*N*-Me)AGIGWP-)

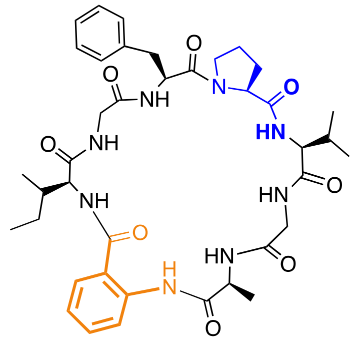

Cyclo(-VGA-2-Abz-IGFP-)

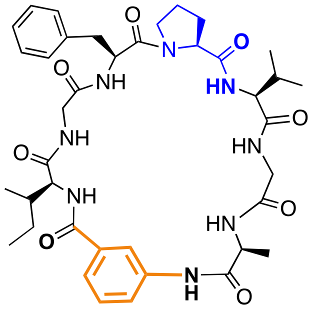

Cyclo(-VGA-3-Abz-IGFP-)

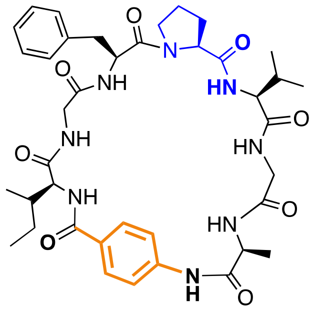

(2) Cyclo(-VGA-4-Abz-IGFP-)

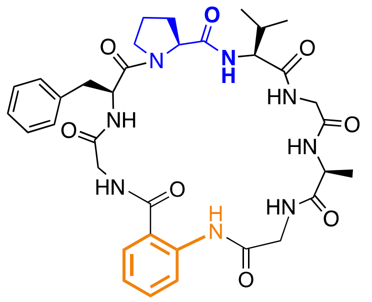

Cyclo(-VGAG-2-Abz-GFP-)

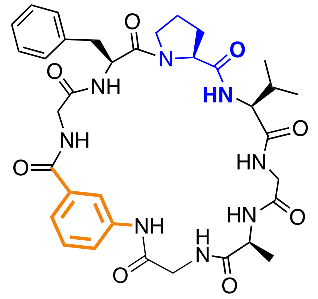

(3) Cyclo(-VGAG-2-Abz-GFP-)

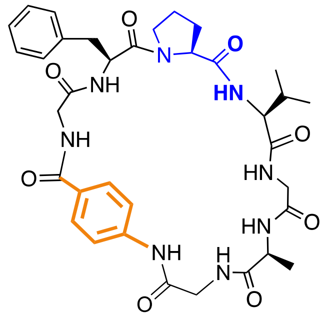

Cyclo(-VGAG-4-Abz-GFP-)

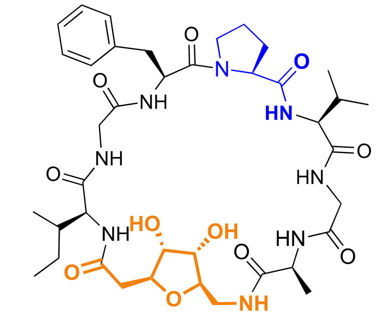

(4) Cyclo(-VGA-Rib-IGFP-)

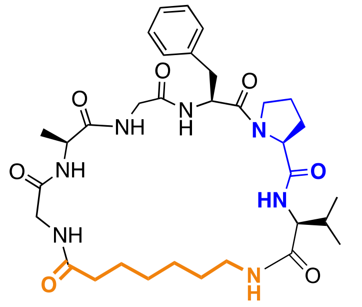

Cyclo(-V-7Ahp-GAGFP-)

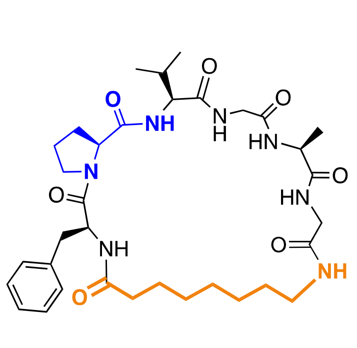

Cyclo(-VGAG-8Aoc-FP-)

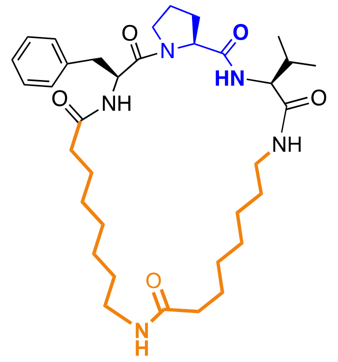

(5) Cyclo(-V-8Aoc-8Aoc-FP-)

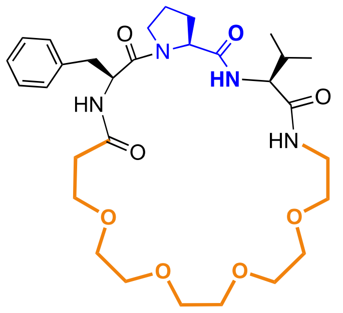

(6) Cyclo(-V-(PEG)$_4$-FP-)

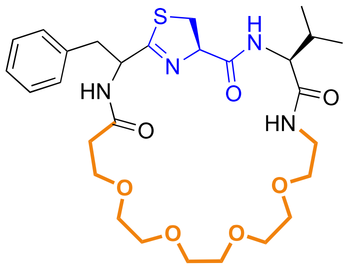

(7a) Cyclo(-V-(PEG)$_4$-FThz-)

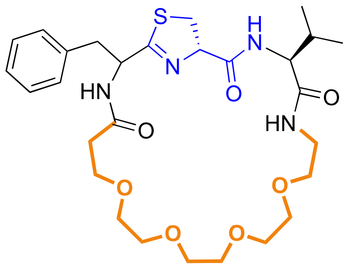

(7b) Cyclo(-V-(PEG)$_4$-FThz-)

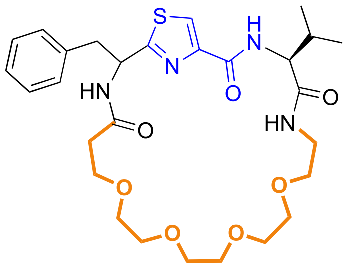

(7c) Cyclo(-V-(PEG)$_4$-FTzl-)

III. Structures of the linearly cleaved hybrid peptides

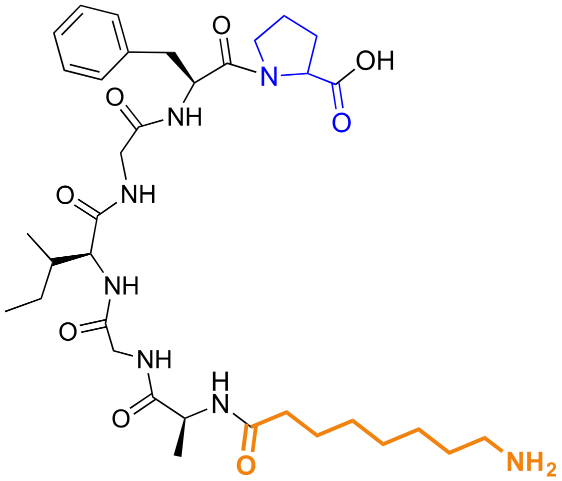

8Aoc-AGIGFP

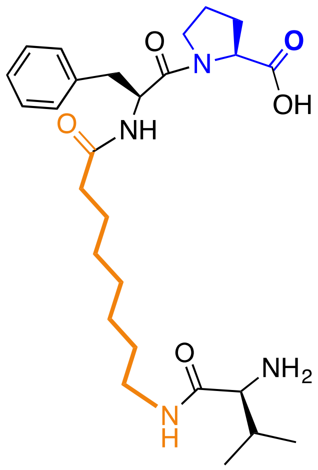

V-8Aoc-FP

IV. General information and materials

The HPLC grade acetonitrile (MeCN) was purchased from Fisher. Aqueous buffers and aqueous mobile-phases for HPLC were prepared using water purified with an Elga® Purelab® Milli-Q water purification system (purified to 18.2 MΩ.cm) and filtered over 0.45 μm filters. Solvents, amino acids and coupling reagents were purchased commercially from different sources and used without any further purification. Anhydrous CH_3CN and MeOH were purchased from Sigma.

NMR spectra (^{1}H, 2D) of the cyclic peptides were recorded on a Bruker Ascend 700 spectrometer {δH (700 MHz), δC (175 MHz)} at ambient temperature in deuterated DMSO. NMR spectra ((^{1}H, 2D, ^{13}C) of the synthetic intermediates were recorded on a Bruker Ascend 500 spectrometer {δH (500 MHz), δC (126 MHz)} at ambient temperature in deuterated $CDCl_3$. Chemical shifts are expressed in parts per million

(ppm) from DMSO-d6 (δH = 2.50, δC = 39.52) or $CDCl_3$ (δH = 7.26, δC = 77.16).[1] Multiplicities are described as s (singlet), d (doublet), q (quadruplet), dd (doublet of doublets), dd (doublet of doublets of doublets), t (triplet), dd (doublet of triplets), m (multiplet), br (broad). Coupling constants *J* are quoted in Hertz (Hz) to the nearest 0.1 Hz. Signals of protons and carbons were assigned, as far as possible, by using the following two dimensional NMR spectroscopy techniques: [^{1}H, ^{1}H] COSY (Correlation Spectroscopy), [^{1}H, ^{1}H] TOCSY (Total Correlation Spectroscopy), [^{1}H, ^{1}H] ROESY (Rotating-frame NOE Spectroscopy), [^{1}H, ^{13}C] HSQC (Heteronuclear Single Quantum Coherence) and long range [^{1}H, ^{13}C] HMBC (Heteronuclear Multiple Bond Connectivity). EXSY (Exchange Spectroscopy) experiment was used to identify equilibrium chemical exchange at rt.

Low-resolution mass spectra were obtained with an Agilent 6130 single quad apparatus equipped with an electrospray ionization source. High-resolution mass spectra (HRMS) were obtained with a Thermo Exactive Orbitrap mass spectrometer or obtained from the EPSRC mass facility in Swansea. Infrared (IR) spectra were recorded on a Shimadzu IRAffinity-1 Fourier transform IR spectrophotometer using Pike MIRacle ATR accessory. Analysis was carried out using Shimadzu IRsolution v1.50 and only characteristic peaks are reported in wavenumbers (cm^{-1}). Optical rotations were measured at 20 °C on a PerkinElmer Precisely/Model-341 polarimeter operating at the sodium D line with a 100 mm path cell. The resulting optical rotation is the average of a duplicate obtained each by averaging 10 readings.

Reactions performed in the enzymatic media were monitored using MALDI-MS acquired using a 4800 MALDI TOF/TOF Analyser (ABSciex, Foster City, CA) equipped with a Nd:YAG 355 nm laser and calibrated using a mixture of peptides. The spot was analysed in positive MS mode between 500 and 4000 m/z, by averaging 1000 laser spots. The samples, diluted in water to reduce the buffer concentration, (0.5 μL) were applied to the MALDI target along with alpha-cyano-4-hydroxycinnamic acid matrix (0.5 μL, 10 mg/mL in 50:50 acetonitrile:0.1% TFA) and allowed to dry. MSMS data were acquired using a TripleTOF 5600+. The sample was subjected to chromatography on an Acclaim PepMap 100 C18 trap and an Acclaim PepMap RSLC C18 column (ThermoFisher Scientific), using a nano-LC Ultra 2D plus loading pump and nano-LC as-2 autosampler (Eksigent). The sample was injected at neutral pH to avoid acid catalyzed ring opening where possible. The trap was washed with 2% acetonitrile, 0.05% trifluoroacetic acid, and the desired peptide

was then eluted with a gradient of increasing acetonitrile, containing 0.1 % formic acid (15-40% acetonitrile in 5 min, 40-95% in a further 1 min, followed by 95% acetonitrile to clean the column, before re-equilibration to 15% acetonitrile). The eluent was sprayed into a TripleTOF 5600+ electrospray tandem mass spectrometer (Sciex) operating with standard nanospray conditions, and analyzed in Product Ion Scan mode isolating the m/z of interest. The collision energy was adjusted to give optimal fragmentation. The MSMS fragmentation pattern was interrogated for diagnostic peaks.

Automated solid-phase peptide synthesis (SPPS) was carried out on a Biotage® Syro Wave™ system in polypropylene (PP) syringe with a PTFE frit. Final cleavage and deprotection were completed manually.

Analytical RP-HPLC was performed on an Agilent infinity 1260 series equipped with either a MWD detector and a single quadrupole MS using a Macherey-Nagel Nucleodur C18 column (10 µm x 4.6 x 250 mm) using the following chromatographic systems. **System A:** 1 mL/min flow rate with MeCN and 0.1 % aqueous TFA [95% TFA (5 min), linear gradient from 5 to 95% of MeCN (35 min), 95% MeCN (40 min)] and UV detection at 220 nm (**A1**) or at 210 nm (**A2**). **System B:** 1 mL/min flow rate with MeCN and 5mM ammonium carbonate (AC) [95% AC (5 min), linear gradient from 5 to 95% of MeCN (35 min), 95% MeCN (40 min)] and UV detection at 220 nm. **System C:** 1 mL/min flow rate with MeCN and 0.1 % aqueous TFA [95% TFA (3 min), linear gradient from 5 to 95% of MeCN (30 min), 95% MeCN (35 min)] and UV detection at 280 nm.

Semi-preparative RP-HPLC was performed on an Agilent Infinity 1260 series equipped with an MWD detector using a Macherey-Nagel Nucleodur C18 column (10 µm x 16 x 250 mm at 10 mL/min or 10 µm x 21 x 250 mm at 21 mL/min) and fractions were collected automatically by peak detection at the specified wavelength using an Agilent 1260 Infinity preparative-scale fraction collector. Two chromatographic systems were used; **System P1**: MeCN and 0.1 % aqueous TFA [95% TFA (5 min), linear gradient from 5 to 95% of MeCN (35 min), 95% MeCN (40 min)] and UV detection at 280 nm for peptides **12**-**26**, 220 nm for hybrid cyclic peptides **1**-**7**, **7c** and 254 nm for intermediate **27**. **System P2**: MeCN and 5mM ammonium carbonate (AC) [95% AC (5 min), linear gradient from 5 to 95% of MeCN (35 min), 95% MeCN (40 min)] and UV detection at 220 nm. The runs were stopped as soon as the products eluted off the column for hybrid cyclic peptides **7**.

V. General procedures

PatGmac cloning, expression and purification

The PatGmac enzyme was cloned from genomic DNA *(Prochlon sp.*) into the pHISTEV vector, expressed in *Escherichia coli* BL21 (DE3) cells grown on auto-induction medium, and purified as previously described by Koehnke *et al*.[2] However, subsequent to the Nickel column eluting with 250 mM imidazole, the remaining purification steps were replaced with dialysis in a bicine buffered solution [20 mM Bicine, 150 mM NaCl, pH 8.1] to remove the imidazole and the reducing agent.

LynDfusion cloning, expression and purification

The LynDfusion was cloned, expressed in *Escherichia coli* BL21 (DE3) cells grown on auto-induction medium, and purified as previously described by Koehnke *et al*.[3]

ArtGox cloning, expression and purification

The ArtGox enzyme was cloned, expressed in *Escherichia coli* BL21 (DE3) cells grown on auto-induction medium, and purified as previously described.[4]

Solid-phase peptide synthesis of peptides 8-26, 36-38

The different precursor peptides were synthesized by standard solid-phase (SPPS) on a Rink amide resin or a Chem-Matrix Rink amide resin (0.47 mmol/g, **12-26, 36-38**) or a tentagel resin (0.21 mmol/g, **8-11**) using the Fmoc strategy and Fmoc-protected amino acids (aa). A double coupling strategy using a 5-fold excess with HBTU/DIEA and DIC/oxyma pure (DIC, 0.5 M in DMF), Oxyma, 1 M in DMF, HBTU, 0.5 M in DMF), and DIEA, 2 M in NMP) was used for all amino acids for 30 minutes at 75 °C. The Fmoc deprotection was done in 20% piperidine/DMF for 12 min at rt.

For the final cleavage and side chain deprotection, the beads (washed with CH_2Cl_2 and dried) were transferred into a flacon tube and the cleavage cocktail was added and left shaking for 2h: 96% TFA, 2.5% H_2O, 1.5% TIS. In the presence of cysteines, 1.5% of 1,2-ethanedithiol (EDT) was also added. The resin was filtered, washed with CH_2Cl_2, and the filtrate concentrated under reduced pressure. Peptides (**12-26**) were then precipitated in cold Et_2O and the precipitate purified by HPLC using system P1. LCMS traces of peptides **12**-**26** can be found in section XI. Peptides (**8-11**) were

analyzed by LCMS and used without any further purification. LC traces and MS spectra of peptides **8-11** can be found in section XII.

8-aminooctanoic acid (8Aoc), 7-aminoheptanoic acid (7Ahp), 2, 3, and 4-aminobenzoic acids (Abz), 8-amino-3,6-dioxaoctanoic acid (Doc), GABA= γ-aminobutyric acid, and all other known amino acids, were all purchased in their Fmoc-protected form. The $(PEG)_4$ amino acidic chain was purchased unprotected and later Fmoc protected. The fully protected Fmoc-sugar amino acid **27** was synthesized starting from ribose in 7 steps.

Pat Gmac macrocylization reaction of peptides 8-25, 36-38

The reactions were conducted in 20 mM bicine buffer, 500 mM NaCl, and 5% DMSO solution, pH 8.1 and incubated at 37 °C (without shaking) until full consumption of the starting peptide occured (MALDI monitoring). The reaction set-up was prepared in the following order; final concentrations:

1- A solution of the linear peptide in DMSO (between 10 and 50 mM); 100 μM
2- DMSO; 5%
3- 20 mM Bicine, 150 mM NaCl, pH 8.1 buffer
4- 5 M NaCl; 500 mM
5- PatGmac enzyme; 60 μM

The small reaction set-ups were just aliquoted and analyzed by MALDI monitoring. The large-scale reaction mixtures were extracted 3 times with *n*-butanol (BuOH): H_2O (1/1, v/v). BuOH was added to the aqueous reaction, vigorously mixed, and then centrifuged for 10 min at high speed to help separate the two phases. The combined BuOH fractions were evaporated under reduced pressure to dryness. The crude material was solubilized in a minimum volume of H_2O/MeCN and immediately purified by HPLC.

One pot heterocyclization / macrocylization reaction of peptide 26

The heterocyclization reaction were first conducted in 20 mM bicine buffer and 150 mM NaCl solution, pH 9.0 and incubated at 27 °C (without shaking) in the presence of ATP and Magnesium Chloride ($MgCl_2$) overnight (MALDI monitoring). The reaction set-up was prepared in the following order; final concentrations:

1- A solution of the linear peptide in DMSO; 110 μM
2- 20 mM Bicine, 150 mM NaCl, pH 9.0 buffer

3- 100 mM ATP; 5 mM

4- 1 M MgCl2; 5 mM

5- LynDfusion enzyme; 5 μM

Then, the salt concentration was increased to 500 mM, DMSO was added (to help with the solubilization of products), and PatGmac enzyme was added to the reaction mixture. Below are the final concentrations for the macrocyclization reaction:

1- Peptide; 90 μM

2- DMSO; 5%

3- 5 mM NaCl; 500 mM

4- PatGmac enzyme; 50 μM

Enzymatic oxidation of thiazoline-containing macrocycle 7a-b

The oxidation reaction were conducted in 20 mM bicine buffer, 500 mM NaCl, and 5% DMSO solution, pH 8.1 and incubated at 27 °C (without shaking) in the presence of FMN and the macrocyclase enzyme. The reaction set-up was prepared in the following order; final concentrations:

1- Thiazoline-containing macrocycle; 100 μM

2- DMSO; 5%

3- 20 mM Bicine, 150 mM NaCl, pH 8.1 buffer

4- 5 M NaCl; 500 mM

5- FMN; 100 μM

6- Oxidase; 20 μM

Same work up/purification as for the macrocyclization reaction followed.

Synthesis of the sugar amino acid (Rib) 27

Two strategies were used for the synthesis of the sugar amino acid 27 depending on the protecting group of the carboxylic acid (Schemes S1, S2, S3).

Scheme S1: Synthesis of intermediates 28 and 34 for both strategies[5].

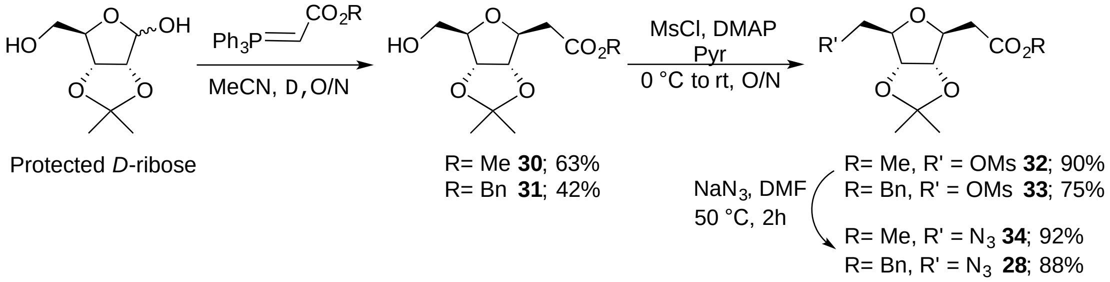

A mixture of diisopropylidene ribose (1 eq.) and methyl or benzyl (triphenylphosphoranylidene) acetate (1.5 eq.) in acetonitrile (1M) was heated to reflux under argon overnight. The solution was concentrated to a small volume then EtOAc was added and the solution washed with water and brine. The organic layer was dried over $MgSO_4$, filtered, and concentrated. The crude mixture was purified over silica gel to afford the corresponding ester.

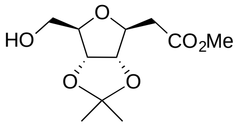

30: Diisopropylidene ribose (4 g, 21 mmol) and methyl(triphenylphosphoranylidene) acetate (10.59 g, 31.6 mmol) in ACN (190 mL) were reacted according to the procedure above. Purification over silica gel afforded the corresponding methyl ester **30** as a yellow oil in 63% yield. The NMR spectroscopic data were in agreement with those described in the literature.[6]

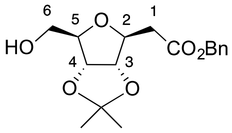

31: Diisopropylidene ribose (1.93 g, 8.1 mmol) and benzyl(triphenylphosphoranylidene) acetate (5 g, 12.2 mmol) in ACN (90 mL) were reacted according to the procedure above. Purification over silica gel afforded the corresponding benzyl ester **31** as a yellow oil in 42% yield. *The other diastereomer is present in a 0.09:1 ratio as determined by ^{1}H NMR. $[\alpha]_D^{20}$ =–18 (c=1.0 in MeOH); ^{1}H NMR (500 MHz, $CDCl_3$) δ=1.33 (s, 3H), 1.54 (s, 3H), 2.34 (dd, J = 4.1, J = 8.5, 1H, OH), 2.68 (dd, J = 6.6, J = 15.9, 1H, CH_2(1)), 2.80 (dd, J = 4.9, J = 15.8, 1H, CH_2(1)), 3.58-3.68 (m, 1H, CH_2(6)), 3.80 (dt, J = 3.3, J = 12.1, 1H, CH_2(6)), 4.07 (q, J = 3.4, 1H, CH(5)), 4.28 (dt, J = 4.9, J = 6.4, 1H, CH_2(2)), 4.55 (dd, J = 4.9, J = 6.7, 1H, CH_2(3)), 4.72 (dd, J = 3.9, J = 6.7, 1H, CH(4)), 5.10-5.20 (m, 2H, CH_2-Bn), 7.30-7.40 (m, 5H, CH_{ar}-Bn); ^{13}C NMR: (126 MHz, $CDCl_3$) δ=25.6 (CH_3), 27.5 (CH_3), 37.8 (CH_2(1)), 62.9 (CH_2(6)), 66.8 (CH_2, Bn), 80.8 (CH(2)), 81.7 (CH(4)), 84.0 (CH(3)), 84.9 (CH(5)), 114.6 (C, *i*-pro), 128.50 (CH_{ar}-Bn), 128.54 (CH_{ar}-Bn), 128.7 (CH_{ar}-Bn), 135.6 (C-Bn), 170.8 (CO-Bn); IR (neat) cm^{-1}: 2939, 1728, 1381, 1257, 1211, 1157, 1064, 864; MS (ESI+) m/z (%): [M+Na^+] 345.2 (30), [M+H_2O^+] 340.2 (100), [M+H^+] 323.2 (90); HRMS (ESI+): m/z calcd for $C_{17}H_{22}O_6$ +Na^+: 345.1309 [M+Na^+]; found: 345.1301; HPLC t_R= 22.50 (purity = 92%; System A2).

Alcohol **30** or **31** (1 eq.) with DMAP (0.5%) in pyridine was cooled to 0 °C and methanesulfonyl chloride (1.1 eq.) was added dropwise under argon atmosphere. The reaction was left to warm to room temperature and was left overnight. Pyridine was concentrated under reduced pressure and water and CH_2Cl_2 were added. The aqueous layer was extracted with CH_2Cl_2 (2x), and the combined organic layers were washed successively with 1 M HCl, aqueous sat. $NaHCO_3$, and brine. The organic layer was dried over $MgSO_4$, filtered, and concentrated. The crude mixture was purified over silica gel to afford the corresponding mesylate.

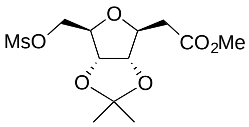

32: Alcohol **30** (3.28 g, 13.3 mmol) and DMAP (8 mg) in Pyridine (24 mL) and MsCl (1.11 mL, 14.4 mmol) were reacted according to the procedure above. Purification over silica gel afforded the corresponding mesylate **32** as a yellow oil in 90% yield. The NMR spectroscopic data were in agreement with those described in the literature.[7]

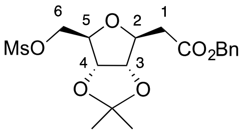

33: Alcohol **31** (2.6 g, 8.1 mmol) and DMAP (5 mg) in Pyridine (15 mL) and MsCl (683 µL, 8.82 mmol) were reacted according to the procedure above. Purification over silica gel afforded the corresponding mesylate **33** as a colorless oil in 75% yield. *The other diastereomer is present in a 0.075:1 ratio as determined by ^{1}H NMR. $[\alpha]_D^{20}$=−4 (*c*=1.0 in MeOH); ^{1}H NMR (500 MHz, $CDCl_3$) δ=1.33 (s, 3H), 1.54 (s, 3H), 2.65 (dd, *J* = 7.2, *J* = 15.8, 1H, CH_2(1)), 2.75 (dd, *J* = 5.2, *J* = 15.8, 1H, CH_2(1)), 2.96 (s, 3H, OMs), 4.13-4.18 (m, 1H, CH(5)), 4.28 (dd, *J* = 4.7, *J* = 11.2, 1H, CH_2(6)), 4.33 (dd, *J* = 3.6, *J* = 11.3, 1H, CH_2(6)), 4.34-4.38 (m, 1H, CH(2)), 4.55 (dd, *J* = 4.3, *J* = 6.8, 1H, CH(3)), 4.60 (dd, *J* = 4.6, *J* = 6.8, 1H, CH_2(4)), 5.15 (q, *J* = 10.8, *J* = 6.4, 2H, CH_2-Bn), 7.30-7.40 (m, 5H, CH_{ar}-Bn); ^{13}C NMR (126 MHz, $CDCl_3$) δ=25.6 (CH_3), 27.5 (CH_3), 37.6 (CH_2(1)), 38.4 (CH_3-OMs), 66.8 (CH_2, Bn), 69.3 (CH_2, (CH_2(6)), 81.2 (2xCH, CH(2), CH(4)), 82.1 (CH(5)), 84.2 (CH(3)), 115.2 (C, *i*-pro), 128.5 (CH_{ar}-Bn), 128.6 (CH_{ar}-Bn), 128.8 (CH_{ar}-Bn), 135.7 (C-Bn), 170.2 (CO-Bn); IR (neat) cm^{-1}: 2940, 1736, 1350, 1165, 1072, 964, 817; MS (ESI+) m/z (%): [*M*+Na^+] 423.1 (20), [*M*+H_2O^+] 418.2 (100), [*M*+H^+] 401.2 (35);

HRMS (ESI+): m/z calcd for $C_{18}H_{24}O_8S+Na^+$: 423.1084 [M+Na^+]; found: 423.1076; HPLC t_R= 25.47 (purity = 99%; System A2).

To a solution of the alcohol (1 eq.) in DMF was added sodium azide (2.5 eq.) and the resulting mixture was stirred at 75 °C for 2 h. The reaction mixture was allowed to cool down to room temperature. Water was added and the reaction extracted with EtOAc (3x). The organic layer was dried over $MgSO_4$, filtered, and concentrated. The crude mixture was purified over silica gel to afford the azide.

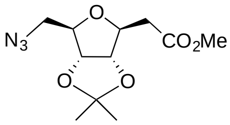

34: Mesylate **32** (3.6 g, 11.1 mmol) and NaN_3 (1.8 g, 27.8 mmol) in DMF (40 mL) were reacted according to the procedure above. Purification over silica gel afforded the corresponding azide **34** as colorless oil in 92% yield. The NMR spectroscopic data were in agreement with those described in the literature.[7]

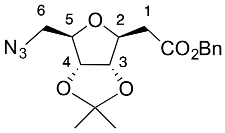

28: Mesylate **33** (2.39 g, 6 mmol) and NaN_3 (970 mg, 14.9 mmol) in DMF (20 mL) were reacted according to the procedure above. Purification over silica gel afforded the corresponding azide **28** as a colorless oil in 88% yield. *The other diastereomer is present in a 0.067:1 ratio as determined by ^{1}H NMR. $[\alpha]_D^{20}$=+30 (c=1.0 in MeOH); ^{1}H NMR (500 MHz, $CDCl_3$) δ=1.33 (s, 3H), 1.53 (s, 3H), 2.71 (dd, J = 6.8, J = 15.9, 1H, CH_2(1)), 2.77 (dd, J = 5.6, J = 15.9, 1H, CH_2(1)), 3.31 (dd, J = 4.8, J = 13.1, 1H, CH_2(6)), 3.51(dd, J = 3.8, J = 13.0, 1H, CH_2(6)), 4.04-4.10 (m, 1H, CH(5)), 4.29-4.34 (m, 1H, CH(2)), 4.54-4.59 (m, 2H, CH(3), CH(4)); 5.11-4.19 (m, 2H, Bn); 7.30-7.40 (m, 5H, CH_{ar}-Bn); ^{13}C NMR (126 MHz, $CDCl_3$) δ=25.6 (CH_3), 27.5 (CH_3), 38.3 (CH_2(1)), 52.3 (CH_2(6)), 66.7 (CH_2, Bn), 80.9 (CH(2)), 82.1 (CH(4)), 83.2 (CH(5)), 84.3 (CH(3)), 115.1 (C, *i*-pro), 128.50 (CH_{ar}-Bn), 128.7 (CH_{ar}-Bn), 135.8 (C-Bn), 170.3 (CO-Bn); IR (neat) cm^{-1}: 2932, 2099, 1735, 1381, 1258, 1211, 1157, 1072, 884; MS (ESI+) m/z (%): [M+Na^+]: 370.2 (35), [M+H_2O^+]: 365.3 (100), [M+H^+]: 348.2 (15); HRMS (ESI+): m/z calcd for $C_{17}H_{21}N_3O_5+Na^+$: 370.1373 [M+Na^+]; found: 370.1368; HPLC t_R= 27.51 (purity = 99%; System A2).

Scheme S2: Strategy 1 for the synthesis of 27 with the methyl ester protection

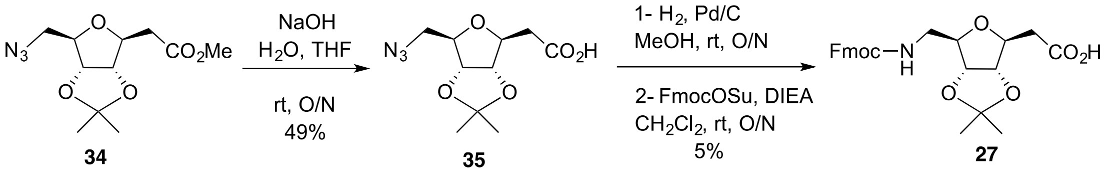

34 **35** **27**

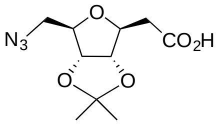

35: To a solution of **34** (150 mg, 0.55 mmol)* in THF (1 mL) was added 1 M aqueous LiOH (0.85 mL). The mixture was stirred overnight and more LiOH (1 mL) was added and the reaction left to complete 24h. HCl 2M was added until pH 2 and the compound was extracted with EtOac (3x). The combined organic layers were washed with brine and then dried over $MgSO_4$, filtered, and concentrated. The crude mixture was purified over silica gel to afford the corresponding carboxylic acid **26** as a colorless oil in 49% yiels. The NMR spectroscopic data were in agreement with those described in the literature.[8]

* When the reaction was set up on 1.5 g, only 55% conversion was observed even if left longer more LiOH added.

27: Azide **35** (450 mg, 1.75 mmol) was solubilized in anhydrous MeOH under argon and 10% Pd/C was added (187 mg, 0.17 mmol). After three cycles of argon/vacuum, the reaction was stirred under $H_{2(g)}$ atmosphere overnight. The reaction was then filtered over celite, washed with MeOH, and evaporated. The crude amine compound was used for the next step without any further purification. The crude was solubilized in CH_2Cl_2 (20 mL) and Fmoc *N*-hydroxysuccinimide ester (FmocOSu; 1.18 g, 3.5 mmol) was added. DIEA (670 μL, 3.85 mmol) was then added slowly to the reaction mixture and left stirring overnight. The reaction was then washed with HCl 1M and brine, dried over $MgSO_4$, filtered, and concentrated. The crude mixture was purified over silica gel to afford the desired Fmoc-protected ribose derivative **27** as a yellow oil in 5% yield. (Improved synthesis in Scheme S3)

Scheme S3: Strategy 2 for the synthesis of 27 with the benzyl ester protection

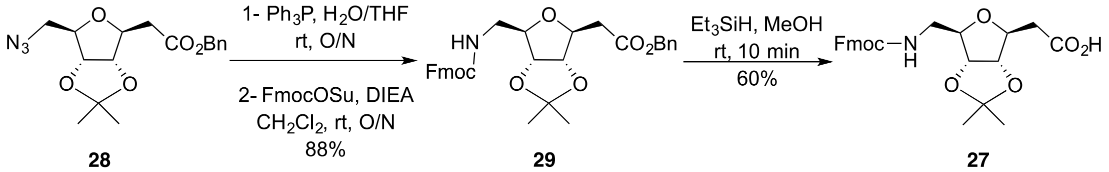

28 **29** **27**

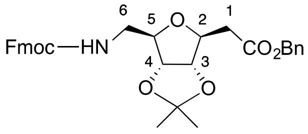

29: To a solution of **28** (1.5 g, 4.3 mmol) in THF/H_2O; 4:1 (51 mL) was added triphenylphosphine (Ph_3P) and the reaction was left stirring overnight. The TFH was evaporated under reduced pressure and the reaction extracted with CH_2Cl_2 (3x). The combined organic layers were dried over $MgSO_4$, filtered, and concentrated. The crude mixture was used for the next step without any further purification. The crude was solubilized in CH_2Cl_2 (26 mL) and FmocOSu (2.92 g, 4.3 mmol) was added. DIEA (900 μL, 8.6 mmol) was then added slowly to the reaction mixture and left stirring overnight. The reaction was then washed with HCl 1M, aqueous sat. $NaHCO_3$, and brine, dried over $MgSO_4$, filtered, and concentrated. The crude mixture was purified over silica gel to afford compound **29*** as colorless oil that crystallized into a white solid overtime in 88% yield over two steps. *The other diastereomer is present in a 0.06:1 ratio as determined by ^{1}H NMR. $[\alpha]_D^{20}$=–9 (c=1.0 in MeOH); ^{1}H NMR (500 MHz, $CDCl_3$) δ=1.32 (s, 3H), 1.53 (s, 3H), 2.65 (dd, J = 6.4, J = 16.0, 1H, CH_2(1)), 2.78 (dd, J = 4.9, J = 16.0, 1H, CH_2(1)), 3.44 (t, J = 5.1, 2H, CH_2(6)), 4.03 (q, J = 4.2, 1H, CH(5)), 4.20-4.28 (m, 2H, CH(2), CH-Fmoc), 4.38-4.45 (m, 2H, CH_2-Fmoc), 4.45-4.50 (m, 1H, CH(4)), 4.50-4.55 (m, 1H, CH(3)), 5.12-5.20 (m, 2H, CH_2-Bn), 5.26-5.36 (m, 1H, NH), 7.27-7.37 (m, 7H, CH_{ar}-Bn (5H), CH_{ar}-Fmoc (2H)), 7.39 (t, J = 7.5, 2H, CH_{ar}-Fmoc), 7.63 (d, J = 7.4, 2H, CH_{ar}-Fmoc), 7.76 (d, J = 7.5, 2H, CH_{ar}-Fmoc); ^{13}C NMR (126 MHz, $CDCl_3$) δ=25.6 (CH_3), 27.5 (CH_3), 37.8 (CH_2(1)), 42.8 (CH_2(6)), 47.4 (CH-Fmoc), 66.8 (2x CH_2, Fmoc, Bn), 80.6 (CH(2)), 82.2 (CH(4)), 83.2 (CH(5)), 84.0 (CH(3)), 115.0 (C, *i*-pro), 120.1 (CH-Fmoc), 125.3 (CH-Fmoc), 127.2 (CH-Fmoc), 127.8 (CH-Fmoc), 128.5 (CH-Bn), 128.6 (CH-Bn), 128.7 (CH-Bn), 135.7 (C-Bn), 141.4 (C-Fmoc), 141.5 (C-Fmoc), 144.0 (C-Fmoc), 144.1(C-Fmoc), 156.8 (CO-Fmoc), 170.5 (CO-Bn); IR (neat) cm^{-1}: 3333, 2947, 2360, 1721, 1551, 1443, 1381, 1250, 1165, 1096, 1057, 1011, 980, 871;

MS (ESI+) m/z (%): [M+Na^+]: 566.3 (35), [M+H_2O^+]: 561.3 (85), [M+H^+]: 544.3 (100); HRMS (ESI+): m/z calcd for $C_{32}H_{33}NO_7+Na^+$: 566.2149[M+Na^+]; found: 566.2137; HPLC t_R= 31.33 (purity = 97%; System A1).

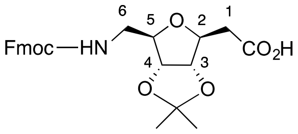

27: Benzyl ester **29** (176 mg, 0.32 mmol) was solubilized in anhydours MeOH under argon and 5% Pd/C was added (137.8 mg, 0.07 mmol). After three cycles of argon/vacuum, and under argon, triethylsilane (517 µL, 3.2 mmol) was added dropwise and very slowly. The generation of $H_{2(g)}$ causes violent bubbling in the reaction mixture. After 10 min. the reaction was over and filtered over celite and washed with MeOH. The crude product was purified by HPLC to afford the desired TFA salt of the Fmoc-protected ribose derivative **27*** as a white solid in 60% yield. *The other diastereomer is present in a 0.05:1 ratio as determined by ^{1}H NMR $[\alpha]_D^{20}$=–5 (c=1.0 in MeOH); ^{1}H NMR (500 MHz, $CDCl_3$) δ=1.33 (s, 3H), 1.53 (s, 3H), 2.60 (dd, J = 7.0, J = 16.1, 1H, CH_2(1)), 2.76 (dd, J = 4.5, J = 16.2, 1H, CH_2(1)), 3.44 (dd, J = 4.8, J = 5.6, 1H, CH_2(6)), 3.96-4.05 (m, 1H, CH(5)), 4.21-4.26 (m, 2H, CH(2), CH-Fmoc), 4.37-4.50 (m, 4H, CH(3), CH(4), CH_2-Fmoc), 5.28 (t, J = 5.2, 1H, NH), 7.31 (td, J = 0.8, J = 7.4, 2H, CH_{ar}-Fmoc), 7.40 (t, J = 7.4, 2H, CH_{ar}-Fmoc), 7.60 (d, J = 7.4, 2H, CH_{ar}-Fmoc), 7.76 (d, J = 7.6, 2H, CH_{ar}-Fmoc); ^{13}C NMR (126 MHz, $CDCl_3$) δ= 25.6 (CH_3), 27.5 (CH_3), 37.5 (CH_2(1)), 42.7 (CH_2(6)), 47.3 (CH-Fmoc), 67.0 (CH_2-Fmoc), 80.4 (CH(2)), 82.2 (CH(4)), 83.4 (CH(5)), 83.9 (CH(3)), 115.1 (C, *i*-pro), 120.2 (CH-Fmoc), 125.2 (CH-Fmoc), 127.2 (CH-Fmoc), 127.9 (CH-Fmoc), 141.4 (C-Fmoc), 141.5 (C-Fmoc), 143.9 (C-Fmoc), 144.0(C-Fmoc), 156.9 (CO-Fmoc), 174.4 (CO-CO_2H); IR (neat) cm^{-1}: 3339, 2987, 2934, 1701, 1530, 1450, 1251, 1209, 1153, 1072, 860, 758; MS (ESI+) m/z (%): [M+Na^+]: 476.2 (35), [M+H^+]: 454.2 (100); HRMS (ESI+): m/z calcd for $C_{25}H_{27}NO_7+H^+$: 454.1860 [M+H^+]; found: 454.1855; m/z calcd for $C_{25}H_{27}NO_7+Na^+$: 476.1680 [M+Na^+]; found: 476.1670; HPLC t_R= 26.32 (purity = 99%; System A2).

VI. MS and HPLC data of starting and final hybrid peptides

Table S1: MS data, HRMS, retention time, HPLC purities, and product information (under PatGmac reaction conditions) of starting hybrid peptides 7-24

	**Peptide**[a]	**MS [M+H]$^+$**	**Calc. HRMS [M+H]$^+$**	**Found HRMS [M+H]$^+$**	**Rt**[b]	**Purity**[c]	**Pdct**[d]	**MS of product [M+H]$^+$**
**8**	VGA-**β-Ala**-IGWPAYD-Doc	1264.8	-	-	14.95	-	C	752.4
**9**	VGA-**GABA**-IGWPAYD-Doc	1278.8	-	-	15.05	-	C	766.4
**10**	VGA-**Doc**-IGWPAYD-Doc	1338.8	-	-	15.28	-	C	826.4
**11**	VG(***N*-Me**)AGIGWPAYD-Doc	1264.6	-	-	15.30	-	C (**1**)	752.4
**12**	VGA-**2-Abz**-IGFPAYD	1127.6	1127.5520	1127.5500	19.23	99%	C	761.4
**13**	VGA-**3-Abz**-IGFPAYD	1127.6	1127.5520	1127.5511	18.50	99%	C	761.4
**14**	VGA-**4-Abz**-IGFPAYD	1127.6	1127.5520	1127.5515	18.33	99%	C (**2**)	761.4
**15**	VGAG-**2-Abz**-GFPAYD	1071.4	1071.4894	1071.4875	17.35	99%	C	705.3
**16**	VGAG-**3-Abz**-GFPAYD	1071.4	1071.4894	1071.4864	16.68	99%	C (**3**)	705.3
**17**	VGAG-**4-Abz**-GFPAYD	1071.4	1071.4894	1071.4877	16.45	99%	C	705.3
**18**	VGA-**Rib**-IGFPAYD	1181.6	1181.5837	1181.5831	16.97	98%	C (**4**)	815.4
**19**	H-**8Aoc**-AGIGFPAYD	1050.5	1050.5619	1050.5600	17.73	99%	L	702.4
**20**	V-**7Ahp**-GAGFPAYD	1022.5	1022.5306	1022.5289	16.77	99%	C	656.4
**21**	VGAG-**7Ahp**-PAYD	875.5	875.4621	875.4605	14.95	99%	NR	-
**22**	VGAG-**8Aoc**-FPAYD	1036.5	1036.5462	1036.5445	17.46	99%	C	670.4
**23**	V-**8Aoc**-FPAYD	851.5	851.4662	851.4646	18.15	99%	L	503.3
**24**	V-**8Aoc-8Aoc**-FPAYD	992.6	992.5815	992.5796	19.48	95%	C (**5**)	626.4
**25**	V-**(PEG)$_4$**-FPAYD	957.5	957.4928	957.4910	17.18	99%	C (**6**)	591.3
**26**	V-**(PEG)$_4$**-FCAYD	963.4	963.4492	963.4489	17.36	95%	C (**7**)	579.3

[a] For structures of the non-natural amino acidic scaffolds, refer to section I.
[b] Retention time as observed by analytical HPLC following system A.
[c] Purity assessed by analytical HPLC at 220 nm UV absorption. LCMS traces of all precursor peptides can be found in section XV.
[d] The product obtained after reaction of the starting peptide with PatGmac as detected by Maldi. C: Cyclic peptide, L: Linear peptide (obtained from the cleavage of the recognition sequence AYD), NR: no reaction.

Table S2: MS data, HRMS, retention time, HPLC purities, and yields of the final cyclic hybrid peptides

	Cyclic Peptide		MS $[M+H]^+$	Calcd HRMS $[M+H]^+$	Found HRMS $[M+H]^+$	Calcd HRMS $[M+Na]^+$	Found HRMS $[M+Na]^+$	rt	Purity (%)[a]	Yield (%)[b]
**1**	VG(***N*-Me**)AGIGWP		752.4	752.4090	752.4103	774.3909	774.3923	21.29	96	51
**2**	VGA-**4-Abz**-IGFP		761.4	761.3981	761.3976	783.3800	783.3793	22.18	93	29
**3**	VGAG-**3-Abz**-GFP		705.3	703.3210[c]	702.3209[c]	727.3194	727.3156	18.90	99	27
**4**	VGA-**Rib**-IGFP		815.4	815.4298	815.4298	837.4117	837.4113	19.44	97	49
**5**	V-**8Aoc-8Aoc**-FP		626.9	626.4276	-	648.4095	648.4088	24.42	95	43 (61)
**6**	V-**(PEG)$_4$**-FP		591.3	591.3388	591.3386	613.3208	613.3199	20.91	98	45 (62)
**7a**	V-**(PEG)$_4$**-FThz	80%	579.3	579.2847	579.2840	601.2666	601.2656	22.18	95[d]	66
**7b**		20%			579.2839		601.2654	22.20	95[d]	14
**7c**	V-**(PEG)$_4$**-FTzl		577.3	577.2690	577.2683	599.2510	599.2496	22.39	98	62

[a] Purity assessed by analytical HPLC at 220 nm UV absorption. LCMS traces can be found in section XIII
[b]The yield of the enzymatic transformation(s) leading to the compound (Corrected yield for 70% conversion).
[c] Calcd and found HRMS of $[M-H]^-$
[d] The main compound peak is 95% pure. For **7a**, <5% of **7b** is present as determined by NMR. For **7b**, 20% of **7a** is present as determined by NMR. The peaks are not separated on analytical LCMS.

Figure S1: HPLC-prep trace of hybrid cyclic peptide 7

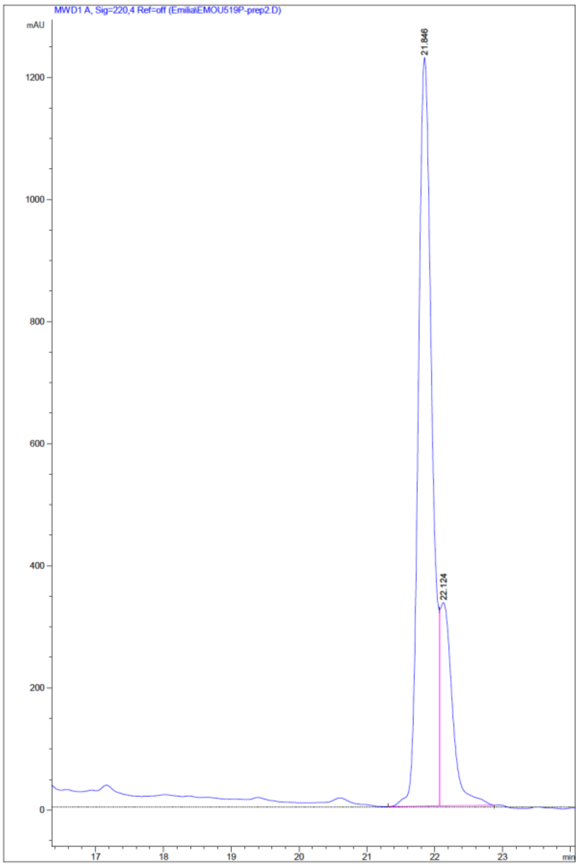

VII. NMR data of final cyclic peptides

Copies of the proton NMR spectra for each compound depicting the different species, when present, by color code as well as copies of the HSQC spectra and EXSY spectra can be found in sections VI and VII.

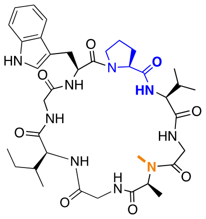

1

1; Cyclo(-VG$_a$(*N*-Me)AG$_b$IG$_c$FP-)

^{1}H NMR: (700 MHz, DMSO); HSQC and HMBC analysis (700 MHz, DMSO)

Amino acid*	Atom	^{1}H chemical shift (CS)	^{13}C chemical shift (CS)
Val	NH	8.02, d, *J* = 8.9	-
	α CH	3.77-3.71, m	61.1
	β CH	2.00-1.95, m	29.5
	γ CH_3	0.79, d, *J* = 6.6;	19.4
		0.77, d, *J* = 6.6	19.7
	CO	-	171.2
Gly (a)	NH	8.30, t, *J* = 5.4	-
	α CH_2	4.01-3.98, m; 3.46-3.43, m	43.3
	CO	-	170.5
Ala	Me(N)	3.01, s	33.4
	α CH	4.48-4.46, m	56.3
	β CH_3	1.35, d, *J* = 7.1	14.4
	CO	-	172.3
Gly (b)	NH	7.17, t, *J* = 4.8	-
	α CH_2	4.05-4.01; 3.77-3.71, m	41.0
	CO	-	170.1
Ile	NH	7.85, d, *J* = 5.9	-
	α CH	4.05-4.01, m[1]	58.3
	β CH	1.94-1.90, m	35.7
	γ CH_2	1.42-1.39, m; 1.26-1.21, m	24.8
	γ CH_3	0.89, d, *J* = 6.9	15.9
	δ CH_3	0.81, t, *J* = 7.4	11.4
	CO	-	171.1
Gly (c)	NH	7.63, t, *J* = 5.4	-
	α CH_2	3.81-3.79, m; 3.77-3.71, m	41.4
	CO		170.8
Trp	NH*	8.37, br	-
	α CH	4.42, dt, *J* = 8.5, *J* = 4.5	54.1
	β CH_2	3.22-3.13, m; 3.10-3.07, m	27.2

1 α-CH signal of Ile and α-CH signal of Gly (b) are overlapped.

	NH[1]-indole	11.0, d, *J* = 1.8	-
	Ar CH	7.42, d, *J* = 7.9	118.7
	Ar CH	7.38, d, *J* = 8.1	111.8
	Ar CH	7.24, d, *J* = 2.2	124.9
	Ar CH	7.09, t, *J* = 7.6	121.6
	Ar CH	6.98, t, *J* = 7.1	118.8
	Ar C	-	109.0
	Ar C	-	127.1
	Ar C	-	136.7
	CO	-	171.1
Pro	α CH	3.37-3.35, m	60.3
	β CH_2	1.60-1.58, m; 0.13-0.09, m	29.8
	γ CH_2	1.36-1.34, m; 1.16-1.14, m	21.7
	δ CH_2	3.22-3.13, m	46.3
	CO	-	170.7

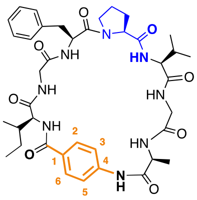

2; Cyclo(-VG$_a$A-4-Abz-IG$_c$FP-)

^{1}H NMR: (700 MHz, DMSO); HSQC and HMBC analysis (700 MHz, DMSO)

**Amino acid***	**Atom**	**^{1}H chemical shift (CS)**	**^{13}C chemical shift (CS)**
Val	NH	7.58, d, *J* = 9.7	-
	α CH	4.36-4.32, m	56.0
	β CH	1.95-1.89, m	31.1
	γ CH_3	0.87-0.84, m	17.7/18.8
	CO		172.3
Gly (a)	NH	8.94, t, *J* = 5.3	-
	α CH_2	3.84, dd, *J* = 4.4, *J* = 14.7	43.2
		3.52-3.47, m	
	CO	-	169.4
Ala	NH	8.89, d, *J* = 7.4	-
	α CH	4.33-4.29, m	49.0
	β CH_3	1.35, d, *J* = 7.4	16.7
	CO	-	171.0
4-Abz	NH	9.38, s	-
	Ar CH	8.00, d, *J* = 8.6 (NH)	118.1
		7.88, d, *J* = 8.7 (CO)	128.4
	Ar C	(C-NH)	129.1
		(C-CO)	141.2
	CO	-	166.0
Ile	NH	8.32, d, *J* = 9.3	-
	α CH	4.35-4.32, m	57.6

	β CH	1.89-1.84, m	35.5
	γ CH_2	1.54-1.43, m; 1.10-1.02, m	24.5
	γ CH_3	0.87-0.82, m	15.4
	δ CH_3	0.87-0.82, m	10.7
	CO	-	171.6
Gly (c)	NH	8.43, t, *J* = 6.2	-
	α CH_2	3.87, dd, *J* = 6.3, *J* = 15.5<br>3.40-3.36, m	42.7
	CO		168.8
Phe	NH	7.97, d, *J* = 9.0	-
	α CH	4.84-4.79, m	51.0
	β CH_2	2.98, dd, *J* = 5.8, *J* = 13.6;<br>2.84, dd, *J* = 8.4, *J* = 13.6	37.6
	Ar CH	7.26-7.22, m ; 7.22-7.18, m	127.8/128.9/126.0
	Ar C		137.2
	CO	-	170.4
Pro	α CH	4.05, dd, *J* = 1.8, *J* = 8.1	58.8
	β CH_2	1.81-1.75, m; 1.36-1.30, m	26.4
	γ CH_2	1.71-1.64, m; 1.54-1.43, m	24.1
	δ CH_2	3.40-3.37, m; 3.32-3.27, m	46.4
	CO	-	169.5

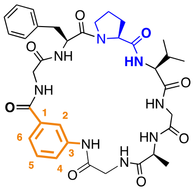

3; Cyclo(-VGaAG$_b$-3-Abz-G$_c$FP-) **2:1**

^{1}H NMR: (700 MHz, DMSO); HSQC and HMBC analysis (700 MHz, DMSO)

Amino acid*	Atom	[1]H CS	[13]C CS	[1]H CS	[13]C CS
Val	NH	7.95-7.92, m	-	7.27-7.23, m	-
	α CH	3.61-3.56, m	59.4	4.14-4.11, m	57.2
	β CH	1.75-1.69, m	28.9	1.87-1.78, m	30.6
	γ CH_3	0.82, d, *J* = 6.8;<br>0.58, d, *J* = 6.7	18.8<br>18.8	0.69, d, *J* = 6.3;<br>0.68-0.62, m	17.8<br>18.9
	CO	-	172.0		ND
Gly (a)	NH	8.17-8.13, m	-	8.13-8.10, m	-
	α CH_2	4.20-4.14, m;<br>3.64-3.58, m	40.7	4.11-4.06, m;<br>3.41, dd, *J* = 4.3, *J* = 16.3	41.4
	CO	-	170.9	-	168.7
Ala	NH	8.60, d, *J* = 3.0	-	8.24, d, *J* = 6.4	-
	α CH	4.02-3.97, m	50.2	4.37-4.32, m	48.3
	β CH_3	1.24, d, *J* = 7.1	16.0	1.23, d, *J* = 7.4	17.8
	CO	-	173.3	-	172.2
Gly (b)	NH	8.86, t, *J* = 6.1	-	8.44, t, *J* = 5.6	-

	α CH_2	4.00-3.85, m; 3.67-3.61, m	43.0	4.05, dd, *J* = 6.7, *J* = 16.4; 3.75, dd, *J* = 5.1, *J* = 16.4	42.4
	CO	-	168.2	-	167.8
3-Abz	NH	9.28, s	-	10.00, s	-
	Ar CH	7.95-7.92, m, (CH-2)	120.3	8.40, s, (CH-2)	118.6
		8.19, dd, *J* = 1.9, *J* = 7.8 (CH-4)	122.4	7.57, s, br, (CH-6)	121.5
		7.65, d, *J* = 7.8 (CH-6)	121.4	7.49, d, *J* = 7.6 (CH-4)	121.9
		7.44-7.39, m, (CH-5)	128.2	7.44-7.39, m, (CH-5)	128.2
	Ar C	-	133.5	-	135.1
		-	138.5	-	ND
	CO	-	166.0	-	166.8
Gly (c)	NH	8.72, t, *J* = 6.0	-	8.76-8.73, m	-
	α CH_2	4.23-4.17, m; 3.64-3.58, m	41.7	3.83-3.77, m; 3.70, dd, *J* = 5.8, *J* = 16.1	43.1
	CO	-	168.0	-	169.0
Phe	NH	8.09, d, *J* = 7.4	-	8.15-8.11, m	-
	α CH	4.56-4.45, m	52.0	4.68-4.60, m	ND
	β CH_2	2.88-2.82, m; 2.81-2.77, m	39.2	3.04, dd, *J* = 5.2, *J* = 13.8; 2.86-2.82, m	36.6
	Ar CH	7.29-6.57, m	128.0 /126.2/ 128.8	7.29-6.57, m	129.1 /126.2/ 127.8
	Ar C		136.9		136.9
	CO	-	169.7	-	ND
Pro	α CH	3.85, dd, *J* = 6.8, *J* = 5.6	59.1	4.31, dd, *J* = 3.1, *J* = 8.5	60.3
	β CH_2	1.70-1.64, m	30.5	2.07-1.95, m; 1.93-1.87, m	28.5
	γ CH_2	1.62-1.54, m; 1.47-1.39, m	22.0	1.92-1.86, m; 1.86-1.82, m	24.3
	δ CH_2	3.29-3.24, m; 3.24-3.18, m	46.0	3.61-3.57, m; 3.48-3.44, m	46.4
	CO	-	170.4	-	170.4

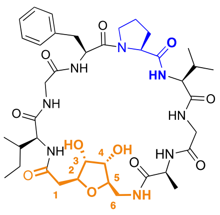

4; Cyclo(-VGaA-Rib-IG$_c$FP-) **2:1**

^{1}H NMR: (700 MHz, DMSO); HSQC and HMBC analysis (700 MHz, DMSO)

**Amino**	**Atom**	**^{1}H CS**	**^{13}C CS**	**^{1}H CS**	**^{13}C CS**

acid*					
Val	NH	7.83-7.78, m	-	7.78-7.73, m	-
	α CH	3.89-3.85, m	59.5	4.15-4.12, m	57.3
	β CH	1.95-1.85, m	29.7	2.07-2.01, m	30.2
	γ CH_3	0.88-0.74, m	19.1<br>18.3	0.88-0.74, m	19.1<br>18.3
	CO	-	170.8	-	171.8
Gly (a)	NH	7.70-7.65, m	-	8.31, t, *J* = 5.7	-
	α CH_2	3.89-3.85, m;<br>3.63-3.55, m	41.5	3.93, d, *J* = 6.9;<br>3.48-3.44, m	41.5
	CO	-	169.3	-	169.1
Ala	NH	8.10, d, *J* = 7.0	-	7.94, d, *J* = 7.5	-
	α CH	4.31-4.26, m	47.9	4.27-4.22, m	48.3
	β CH_3	1.23, d, *J* = 7.1	17.7	1.21, d, *J* = 7.1	17.7
	CO	-	172.3	-	172.3
Rib	NH	7.83-7.78, m	-	7.83-7.78, m	-
	2x OH	4.85-4,80, m	-	4.76-4.71, m	-
	CH_2(6)	3.55-3.44, m;<br>2.70-2.62, m	41.4	3.28-3.18, m;<br>3.07-3.01, m	40.9
	CH(5)	3.71-3.63, m	83.0	3.63-3.54, m	82.0
	CH(4)	3.71-3.63, m	71.7	3.63-3.54, m	71.4
	CH(3)	3.89-3.85, m	73.2	3.63-3.54, m	73.6
	CH(2)	3.84-3.76, m	79.2	3.84-3.76, m	79.2
	CH_2(1)	2.25, d, *J* = 5.6, *J* = 14.1; 2.55-2.51, m	38.1	2.55-2.46, m; 2.42-2.37, m	39.2
	CO	-	170.2	-	170.5
Ile	NH	7.61, d, *J* = 8.5	-	8.00, d, *J* = 6.7	-
	α CH	4.14-4.09, m	56.6	4.00, d, *J* = 7.0	57.8
	β CH	1.67-1.62, m	36.0	1.74-1.67, m	35.6
	γ CH_2	1.51-1.39, m;<br>1.32-1.05, m	24.4	1.51-1.39, m;<br>1.32-1.05, m	24.4
	γ CH_3	0.88-0.74, m	14.9	0.88-0.74, m	15.0
	δ CH_3	0.88-0.74, m	10.7	0.88-0.74, m	10.7
	CO	-	172.3	-	171.9
Gly (c)	NH	8.26, t, *J* = 5.8	-	8.53, t, *J* = 6.0	-
	α CH_2	3.85-3.78, m;<br>3.63-3.55, m	40.6	3.92-3.89, m;<br>3.35-3.30, m	42.0
	CO	-	169.5	-	169.2
Phe	NH	8.41, d, *J* = 5.5	-	7.91, d, *J* = 8.4	-
	α CH	4.49-4.43, m	52.2	4.67-4.62, m	52.2
	β CH_2	2.94-2.81, m	37.7	2.96, dd, *J* = 4.0, *J* = 13.9; 2.94-2.81, m	36.4
	Ar CH	7.32-7.15, m	127.3<br>128.8	7.32-7.15, m	126.7<br>128.4
	Ar C		129.5<br>136.5		129.3<br>138.0
	CO	-	170.4	-	ND
Pro	α CH	3.71-3.63, m	59.8	4.43-4.40, m	59.9
	β CH_2	1.88-1.82, m;<br>1.32-1.25, m	30.3	1.95-1.82, m	26.8

	γ CH_2	1.67-1.62, m; 1.48-1.36, m	21.4	2.03-1.95, m	24.6
	δ CH_2	3.34-3.31, m	45.9	3.63-3.55, m; 3.55-3.44, m	46.6
	CO	-	170.6	-	170.6

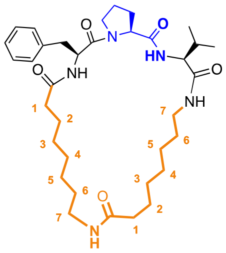

5; Cyclo(-V-8Aoc-8Aoc-FP-) **1.4:1**

^{1}H NMR: (700 MHz, DMSO); HSQC and HMBC analysis (700 MHz, DMSO)

**Amino acid***	**Atom**	**^{1}H CS**	**^{13}C CS**	**^{1}H CS**	**^{13}C CS**
Phe	NH	8.28, d, *J* = 8.1	-	8.19, d, *J* = 7.0	-
	α CH	4.68-4.65, m	52.0	4.54, q, *J* = 7.4	52.0
	β CH_2	3.02-3.00, m; 2.77, dd, *J* = 9.6, *J* = 13.9	36.3	2.90-2.83, m	37.5
	Ar CH	7.29-7.19, m	129.5 128.4 126.9 138.4	7.29-7.19, m	129.5 128.4 126.9 137.0
	Ar C				
	CO	-	170.9	-	170.9
Pro	α CH	4.38, dd, *J* = 3.6, *J* = 8.0	59.7	4.01-3.97, m	60.0
	β CH_2	1.97-1.92, m; 1.88-1.84, m	28.2	1.87-1.83, m; 1.36-1.32, m	30.2
	γ CH_2	1.92-1.82, m	24.1	1.69-1.63, m; 1.53-1.50, m	21.2
	δ CH_2	3.63-3.60, m; 3.58-3.55, m	46.5	3.39-3.30, m	45.8
	CO		171.2		171.2
Val	NH	7.63, d, *J* = 8.8	-	7.63, d, *J* = 8.8	-
	α CH	4.01-3.97, m	58.2	4.01-3.97, m	58.2
	β CH	2.02-2.01, m	29.7	2.02-2.01, m	29.7
	γ CH_3	0.83, dd, *J* = 1.6, *J* = 6.8	19.0 17.8	0.78, t, *J* = 7.3	19.0 17.8
	CO		170.4		170.4
8Aoc-8Aoc	2xNH	7.29-7.68, m	-		
	2xCH_2(7)	3.28-3.23, m; 2.90-2.83, m	37.5		
		3.10-3.06, m; 2.95-2.93, m	38.1		
		2.99-2.95, m	38.4		

		3.05-3.02, m	37.6		
	2xCH$_2$(6)	1.41-1.27, m	24.5		
	2xCH$_2$(5-4-3)	1.25-1.12, m	28.5/28.2/28.0		
	2xCH$_2$(2)	1.55-1.42, m	24.6		
	2xCH$_2$(1)	2.09-1.99, m	34.2/34.9		
	2xCO	-	172.6/172.3	-	

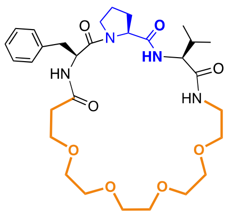

6; Cyclo(-V-(PEG)$_4$-FP-) **1.5:1**

^{1}H NMR: (700 MHz, DMSO); HSQC and HMBC analysis (700 MHz, DMSO)

**Amino acid***	**Atom**	**^{1}H CS**	**^{13}C CS**	**^{1}H CS**	**^{13}C CS**
Phe	NH	8.13, d, *J* = 7.8	-	8.29, d, *J* = 6.9	-
	α CH	4.68-4.63, m	52.3	4.52, q, *J* = 7.4	52.1
	β CH$_2$	3.08-3.03, m; 2.80, dd, *J* = 10.1, *J* = 14.0	36.4	2.91-2.83, m	37.6
	Ar CH	7.34-7.18, m	129.0/128.0/126.2	7.34-7.18, m	129.0/128.0/126.2
	Ar C		138.1		137.1
	CO	-	171.9	-	170.6
Pro	α CH	4.40, t, *J* = 6.0	60.1	3.89, d, *J* = 7.6	60.1
	β CH$_2$	2.00-1.96, m	27.8	1.87-1.83, m; 1.37-1.29, m	30.4
	γ CH$_2$	1.94-1.87, m	24.5	1.69-1.63, m; 1.53-1.45, m	21.2
	δ CH$_2$	3.69-3.65, m; 3.62-3.55, m	46.7	3.83-3.52, m; 3.34-3.31, m	45.7
	CO		171.2		171.0
Val	NH	7.53, d, *J* = 8.7	-	7.67-7.65, m	-
	α CH	4.04, dd, *J* = 5.9, *J* = 8.6	57.8	3.95, dd, *J* = 7.0, *J* = 7.9	58.5
	β CH	2.12-2.08, m	29.5	2.04-2.00, m	29.6
	γ CH$_3$	0.84, t, *J* = 6.9	19.0 17.4	0.80, d, *J* = 3.5, *J* = 6.8	19.0 18.1
	CO		170.8		170.8
PEG$_4$	NH	7.70-7.66, m	-	7.70-7.66, m	-
	CH$_2$(NH)	3.39-3.33, m; 3.10-3.03, m	38.4	3.47-3.36, m; 3.17, q, *J* = 6.2	38.4
	CH$_2$(CO)	2.39-2.30, m 2.17-2.12, m	35.5	2.39-2.30, m	35.5

	$CH_2(CH_2CO)$	3.62-3.57, m<br>3.48-3.44, m	66.4	3.56-6.51, m	66.1
	7x CH_2	3.62-3.33, m	70.0-68.3	3.62-3.33, m	70.0-68.3
	CO	-	171.0	-	170.6

7; Cyclo(-V-(PEG)$_4$-FThz-); **7a**:**7b**/**4:1**

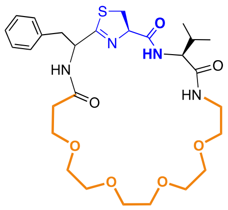

7a: ^{1}H NMR: (700 MHz, DMSO); HSQC and HMBC analysis (700 MHz, DMSO)*

**Amino acid***	**Atom**	**^{1}H CS**	**^{13}C CS**
Phe	NH	8.45, d, *J* = 8.0	-
	α CH	4.84-4.80, m	52.7
	β CH_2	3.08, dd, *J* = 5.7, *J* = 13.8<br>2.97, dd, *J* = 9.3, *J* = 13.7	38.1
	Ar CH	7.28-7.25, m<br>7.22-7.18, m	128.9/128.0<br>126.2
	Ar C	-	137.6
Thz	α CH	5.01, t, *J* = 8.8	77.9
	β CH_2	3.61-3.56, m;<br>3.32-3.28, m	35.5
	CN	-	175.7
	CO	-	170.3
Val	NH	7.48, d, *J* = 9.2	-
	α CH	4.11, dd, *J* = 9.0, *J* = 7.6	57.9
	β CH	2.02-1.92, m	30.3
	γ CH_3	0.85, d, *J* = 6.7<br>0.82, d, *J* = 6.7	19.0<br>17.9
	CO	-	170.6
PEG_4	NH	8.01, t, *J* = 5.6	-
	$CH_2(NH)$	3.37-3.31, m;<br>3.15-3.10, m	38.3
	$CH_2(CO)$	2.41-2.34, m;<br>2.22-2.15, m	35.7
	$CH_2(CH_2CO)$	3.64-3.58, m<br>3.55-3.50, m	66.1
	7x CH_2	3.52-3.40, m	70.3-68.2
	CO	-	170.8

*NMR data of the major compound **7a**

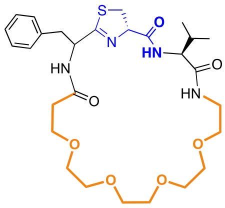

7b:^{1}H NMR: (700 MHz, DMSO); HSQC and HMBC analysis (700 MHz, DMSO)*

**Amino acid***	**Atom**	**^{1}H CS**	**^{13}C CS**
Phe	NH	8.31, d, *J* = 8.5	-
	α CH	4.81-4.76, m	52.1
	β CH_2	3.17, dd, *J* = 4.7, *J* = 13.9	38.0
		2.92, dd, *J* = 9.7, *J* = 13.9	
	Ar CH	7.29-7.24, m	127.8/129.0
		7.21-7.18, m	126.2
	Ar C		138.0
Thz	α CH	5.18, t, *J* = 8.8	77.7
	β CH_2	3.54-3.44, m	34.7
	CN	-	174.5
	CO	-	170.2
Val	NH	7.61, d, *J* = 9.1	-
	α CH	4.20, dd, *J* = 9.0, *J* = 6.9	57.3
	β CH	1.97-1.92, m	31.0
	γ CH_3	0.86, d, *J* = 6.9	19.0
		0.85, d, *J* = 6.9	17.9
	CO		170.8
PEG_4	NH	8.13, t, *J* = 5.5	-
	CH_2(NH)	3.38-3.33, m;	38.4
		3.10-3.04, m	
	CH_2(CO)	2.45-2.40, m;	35.7
		2.26-2.20, m	
	CH_2(CH_2CO)	3.58-3.52, m	66.3
		3.49-3.44, m	
	7x CH_2	3.52-3.39, m	68.0-70.1
	CO	-	170.7

*NMR data of the minor compound **7b**

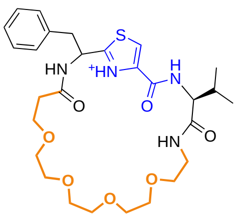

7c: ^{1}H NMR: (700 MHz, DMSO); HSQC and HMBC analysis (700 MHz, DMSO)*

**Amino acid***	**Atom**	**^{1}H CS**	**^{13}C CS**
Phe	NH	8.80, d, *J* = 8.5	-
	α CH	5.37-5.32, m	50.9
	β CH_2	3.43-3.38, m	37.9
		3.29-3.21, m	
	Ar CH	7.33, d, *J* = 7.8;	129.2
		7.25, t, *J* = 7.4;	127.9
		7.18, t, *J* = 7.3	126.2
	Ar C	-	138.3
Tzl	CH	8.19, d, *J* = 1.5	124.2
	^{4}C(C-CO)	-	148.3
	CN	-	170.9
	CO	-	159.9
Val	NH	7.94, d, *J* = 9.4	-
	α CH	4.40-4.36, m	57.3
	β CH	2.05-1.98, m	31.2
	γ CH_3	0.90, d, *J* = 5.5	18.9
		0.91, d, *J* = 5.5	18.1
	CO	-	170.9
PEG_4	NH	8.34, t, *J* = 5.2	-
	CH_2(NH)	3.57-3.52, m;	38.5
		3.01-2.95, m	
	CH_2(CO)	2.44-2.37, m;	35.7
		2..09, dt, *J* = 4.1, *J* = 14.4	
	CH_2(CH_2CO)	3.59-3.54, m	66.7
		3.50-3.45, m	
	7x CH_2	3.53-3.16, m	70-68.4
		3.08-3.03, m	
	CO	-	170.9

VIII. NMR spectra of final cyclic peptides

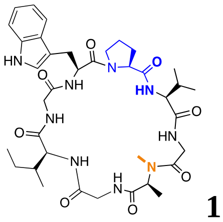

1; Cyclo(-VG$_a$(*N*-Me)AG$_b$IG$_c$WP-); DMSO-d6; 700 MHz

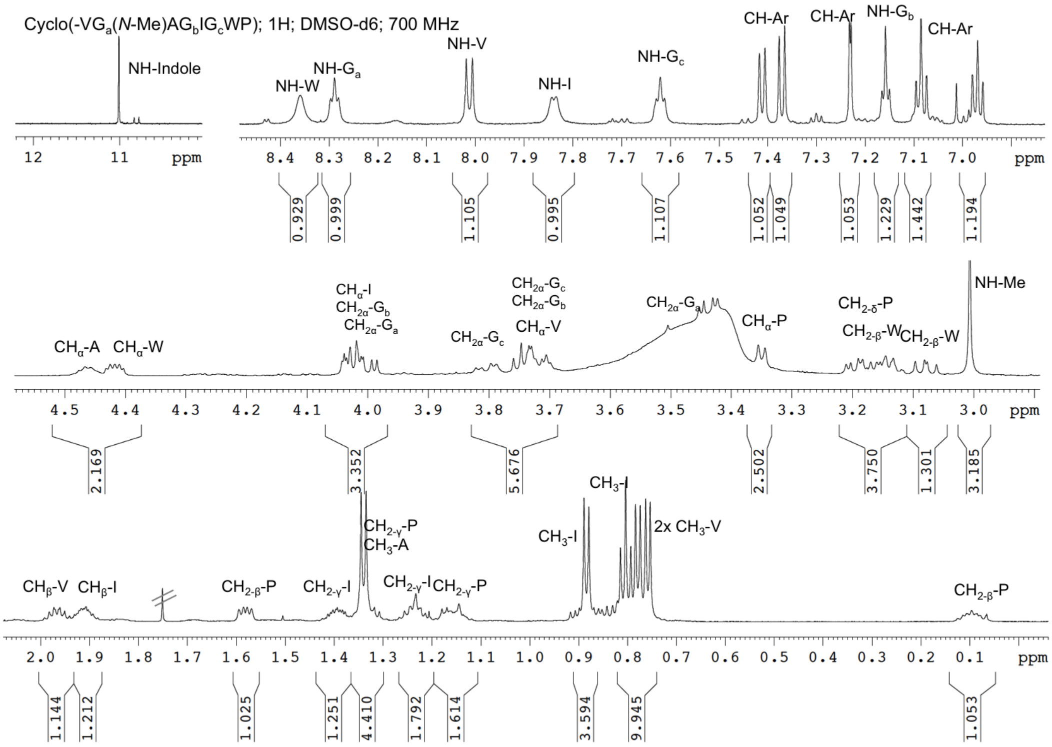

1; Cyclo(-VG$_a$(*N*-Me)AG$_b$IG$_c$WP-); HSQC (green/blue) and HMBC (red) spectra

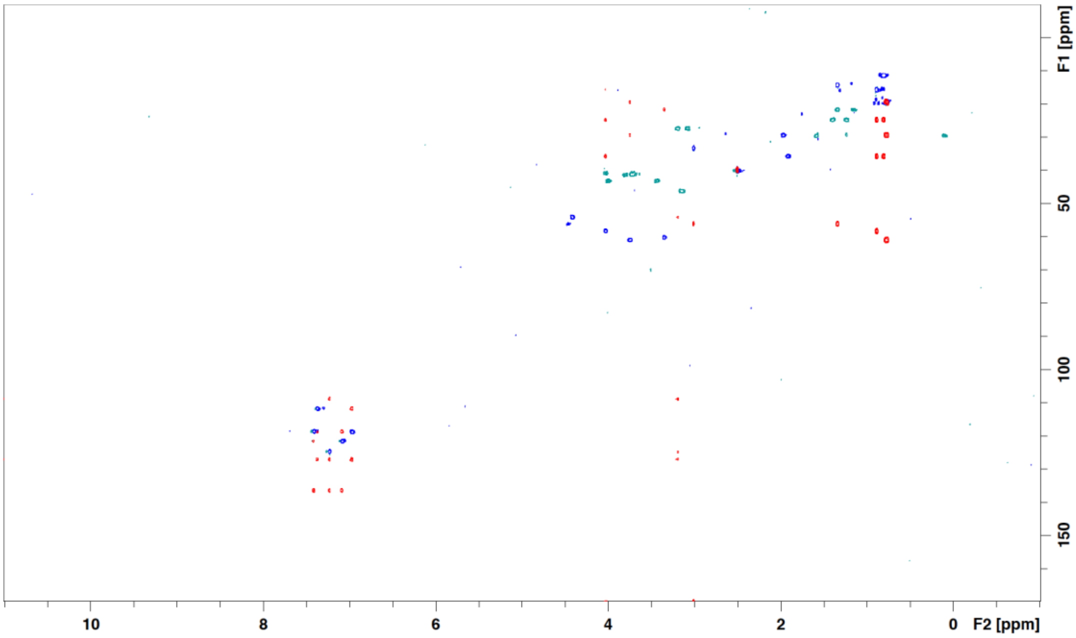

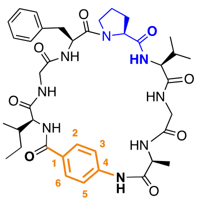

2; Cyclo(-VG$_a$A-4-Abz-IG$_c$FP-); DMSO-d6; 700 MHz

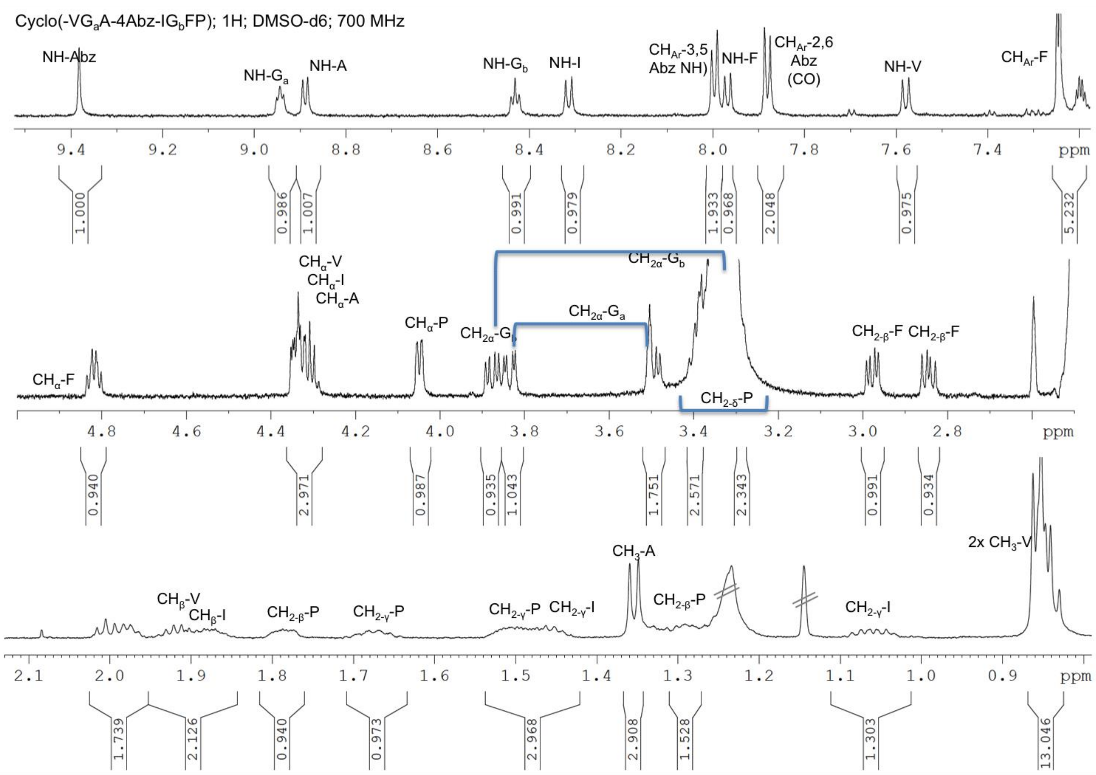

2; Cyclo(-VG$_a$A-4-Abz-IG$_c$FP-); HSQC (green/blue) and HMBC (red) spectra

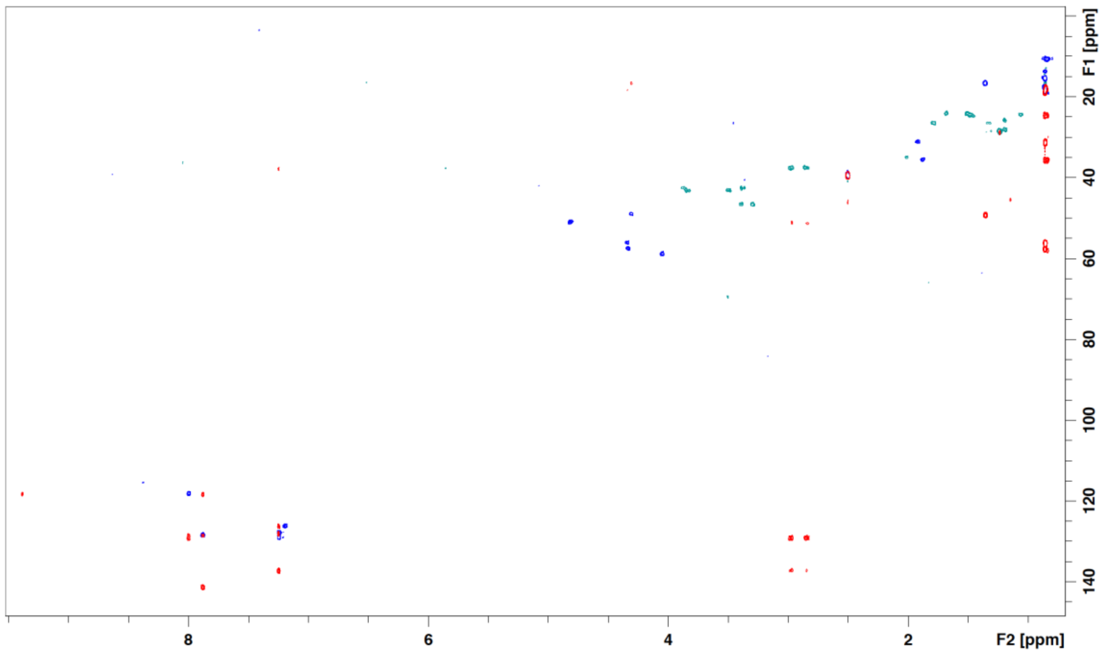

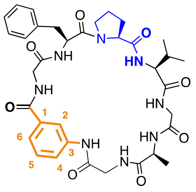

3; Cyclo(-VG$_a$AG$_b$-3-Abz-G$_c$FP-) 2:1; DMSO-d6; 700 MHz

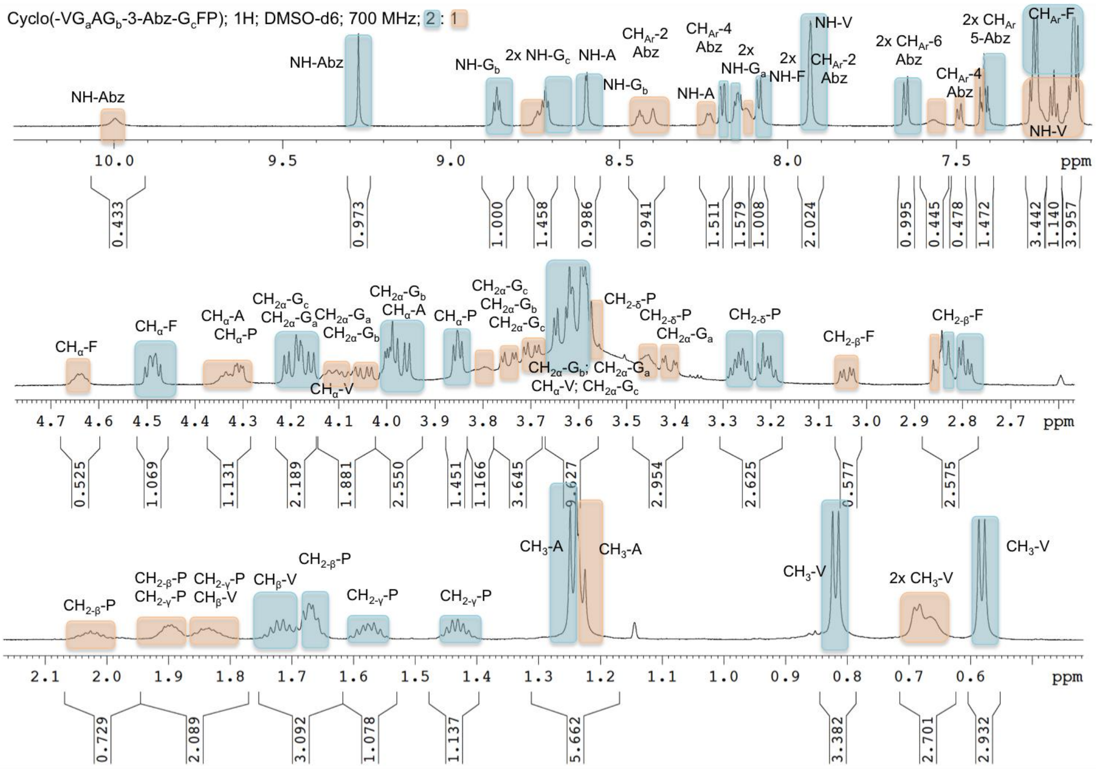

3; Cyclo(-VG$_a$AG$_b$-3-Abz-G$_c$FP-) 2:1; HSQC (green/blue) and HMBC (red) spectra

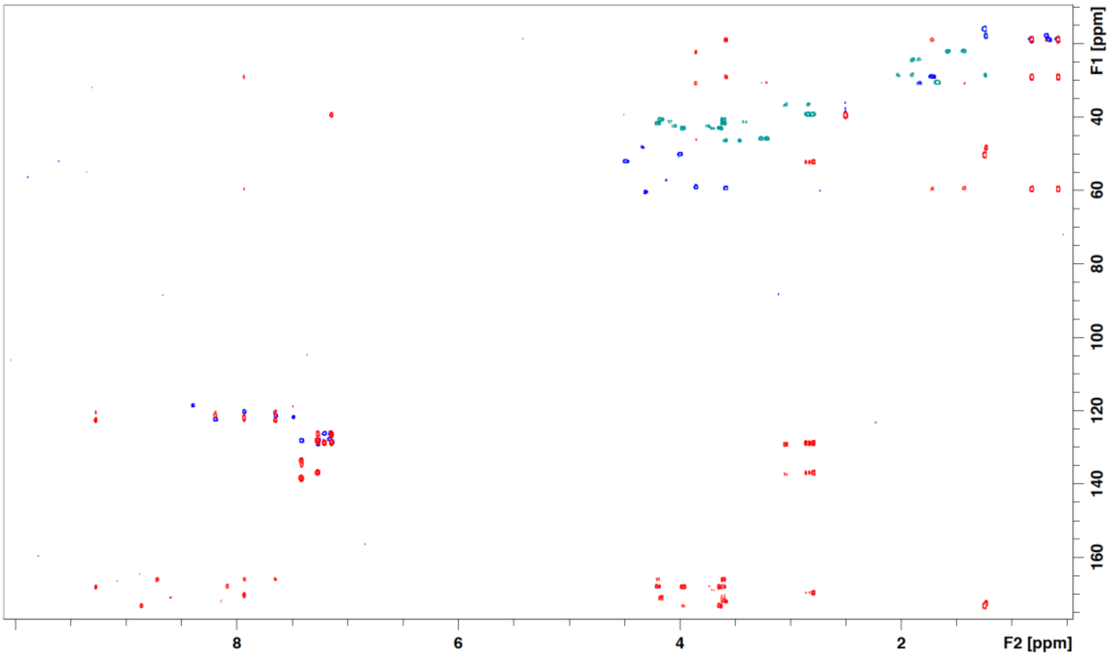

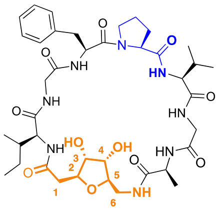

4; Cyclo(-VG$_a$A-Rib-IG$_c$FP-) 2:1; DMSO-d6; 700 MHz

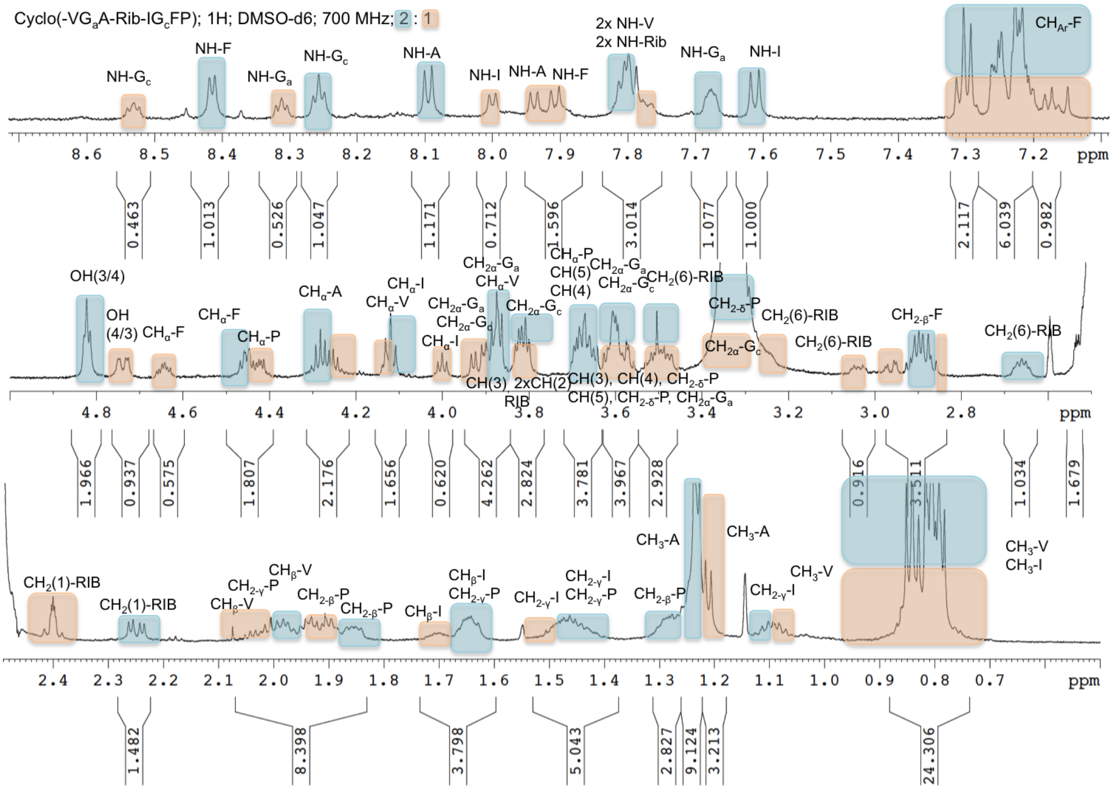

4; Cyclo(-VG$_a$A-Rib-IG$_c$FP -) 2:1; HSQC (green/blue) and HMBC (red) spectra

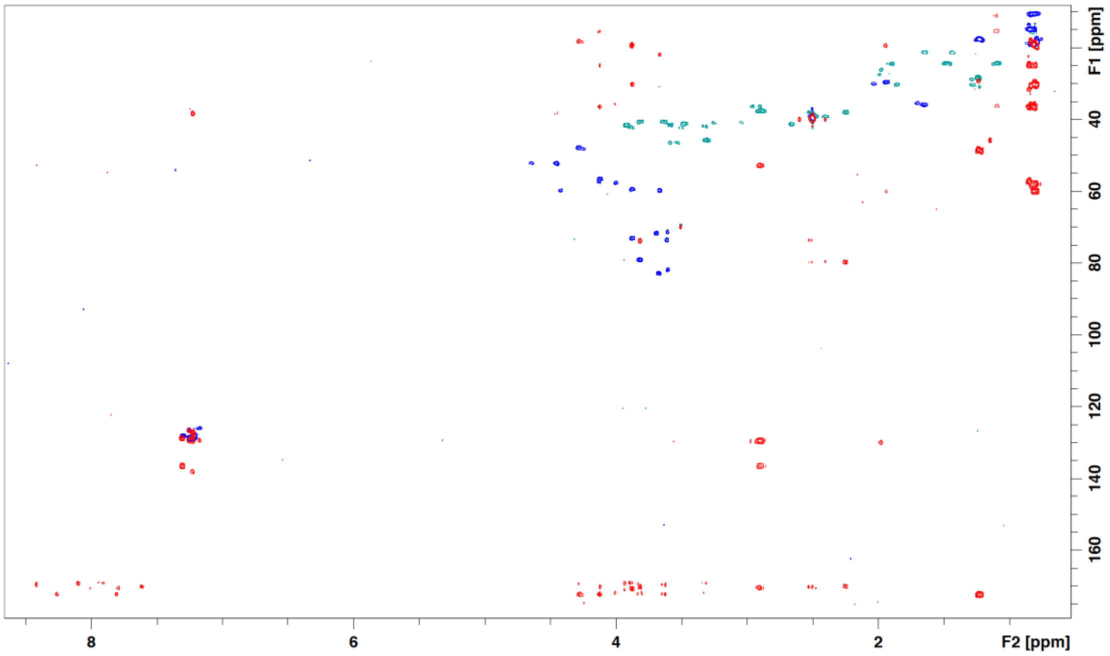

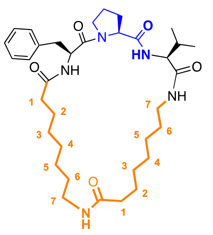

5; Cyclo(-V-8Aoc-8Aoc-FP-) 1.4:1; DMSO-d6; 700 MHz

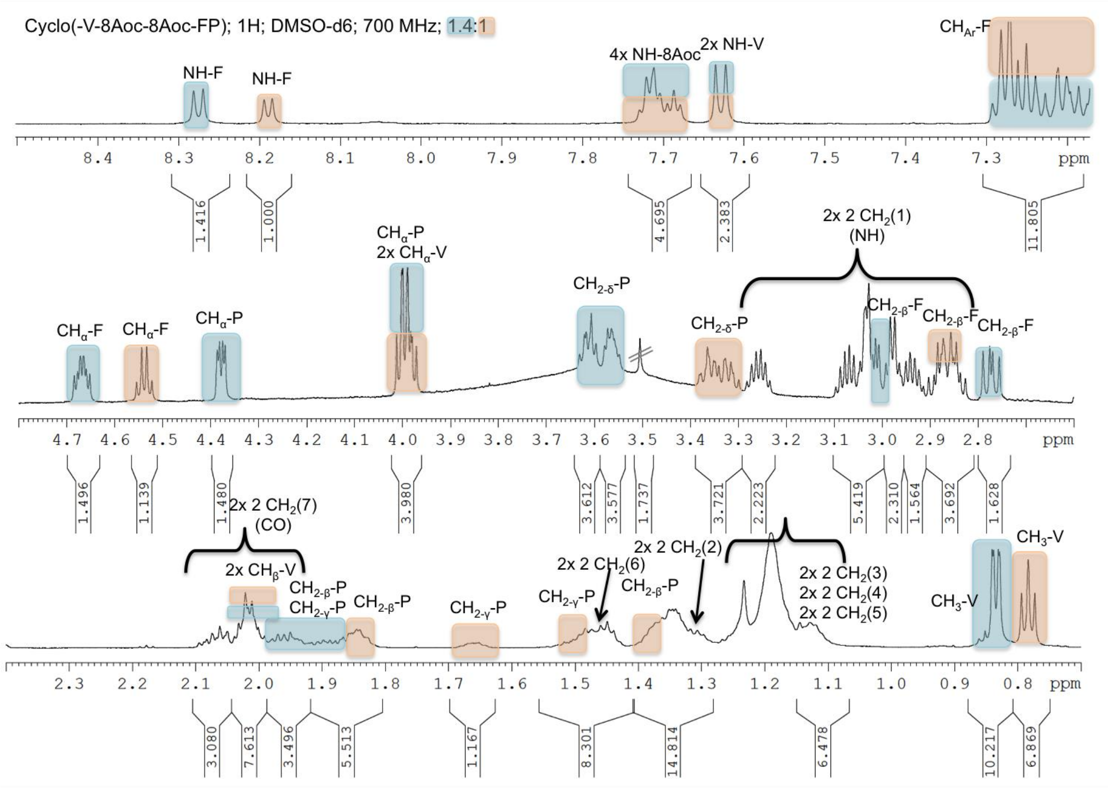

5; Cyclo(-V-8Aoc-8Aoc-FP-) 1.4:1; HSQC (green/blue) and HMBC (red) spectra

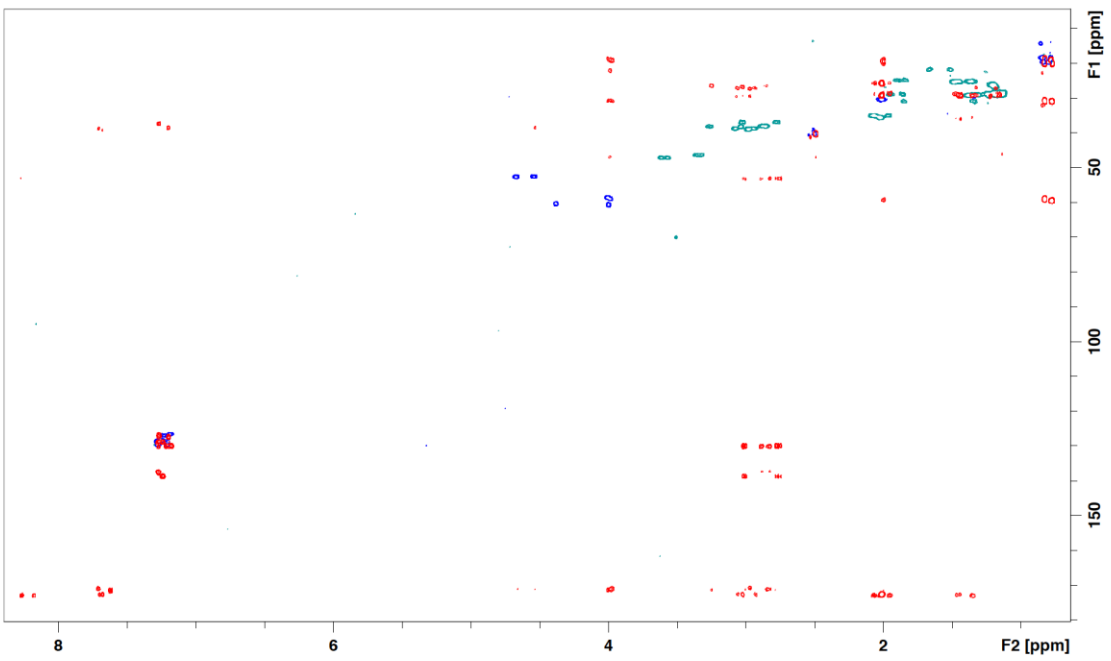

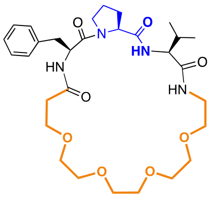

6; Cyclo(-V(PEG)$_4$FP-) 1.5:1; DMSO-d6; 700 MHz

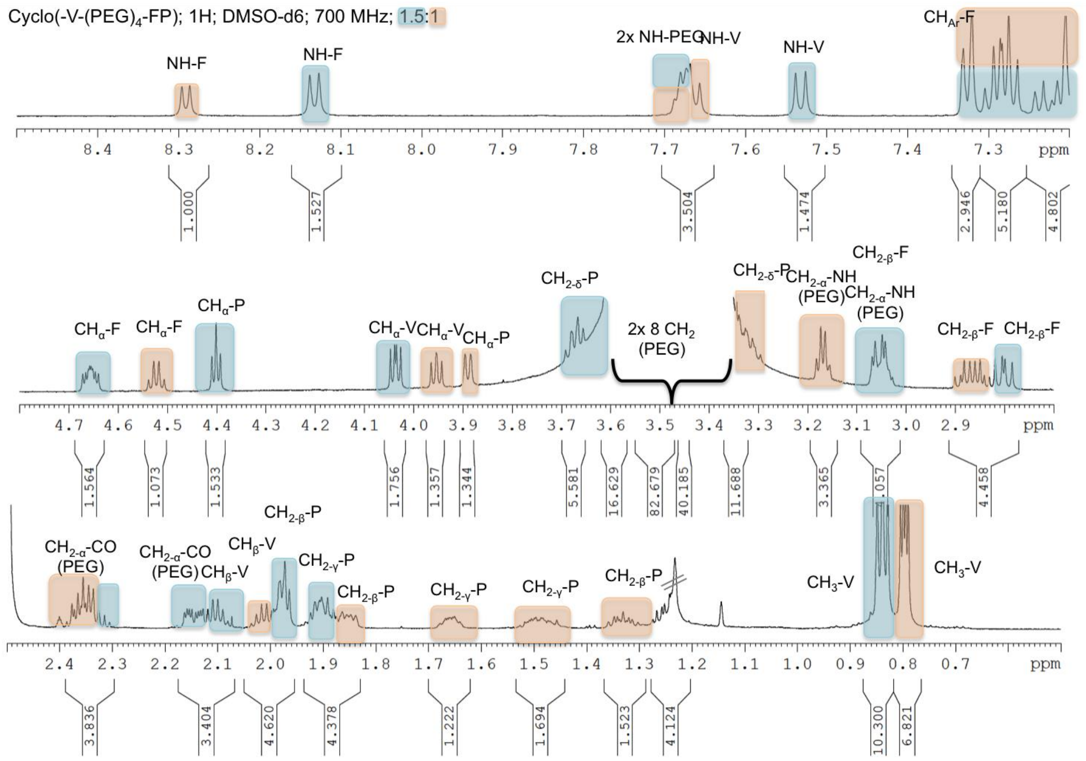

6; Cyclo(-V(PEG)$_4$FP-) 1.5:1; HSQC (green/blue) and HMBC (red) spectra

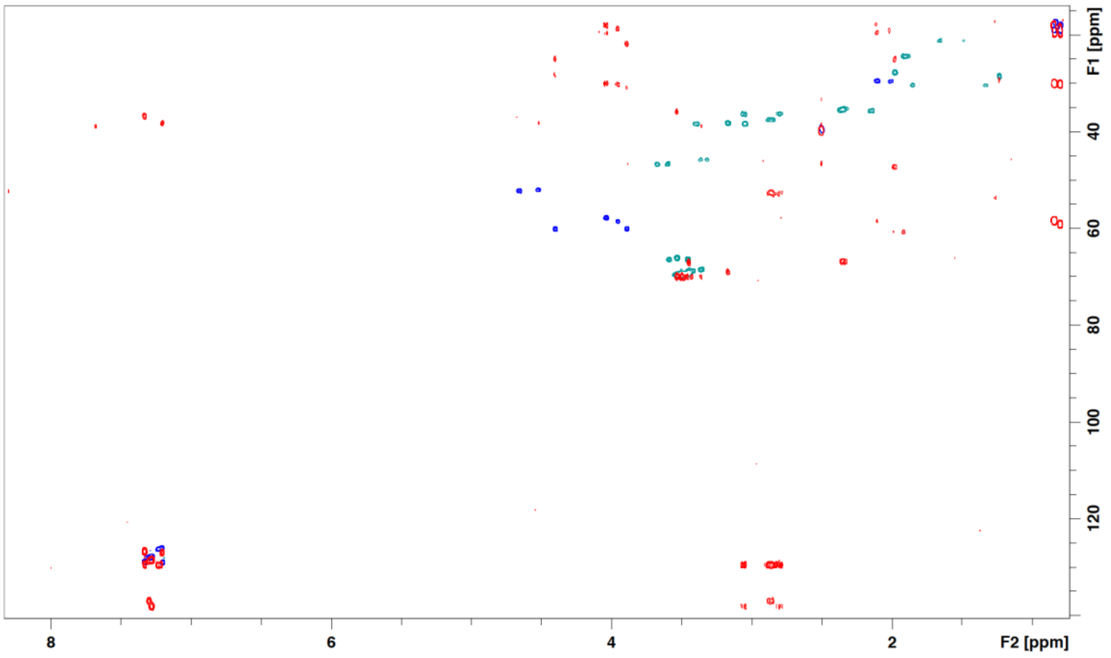

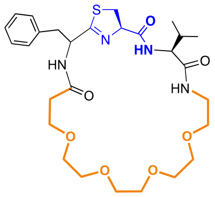

7a; Cyclo(-V(PEG)$_4$FThz-); DMSO-d6; 700 MHz (<5% **6b**)

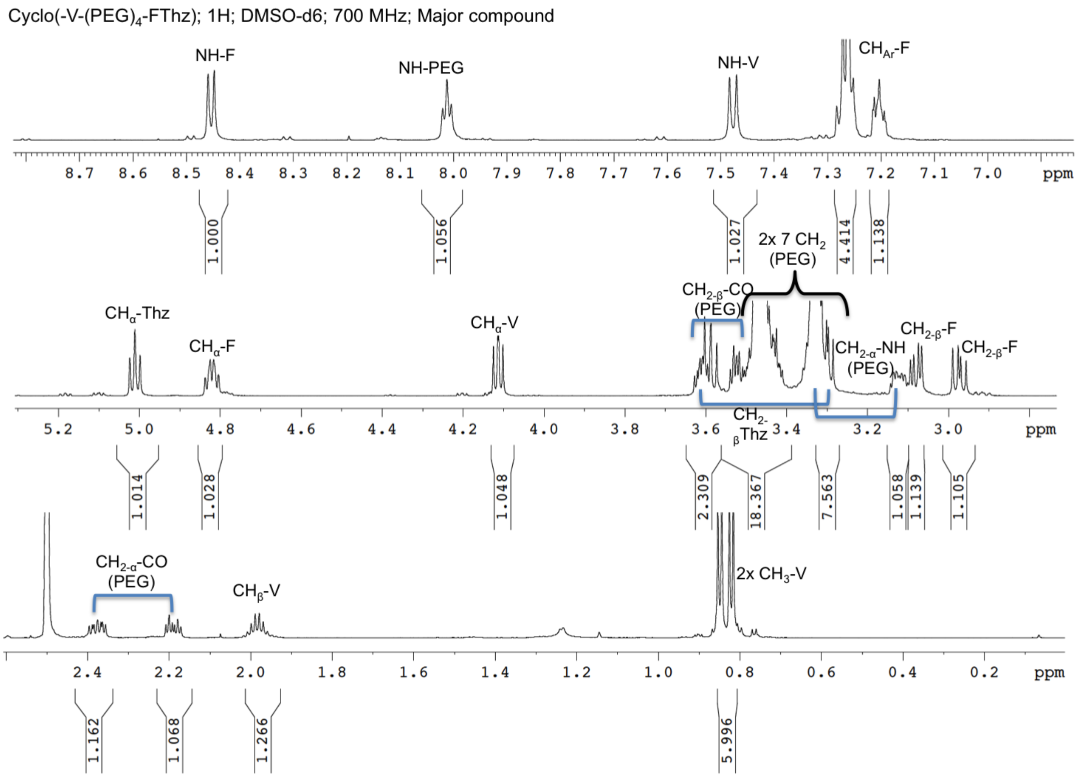

7a; Cyclo(-V(PEG)$_4$FThz-); HSQC (green/blue) and HMBC (red) spectra

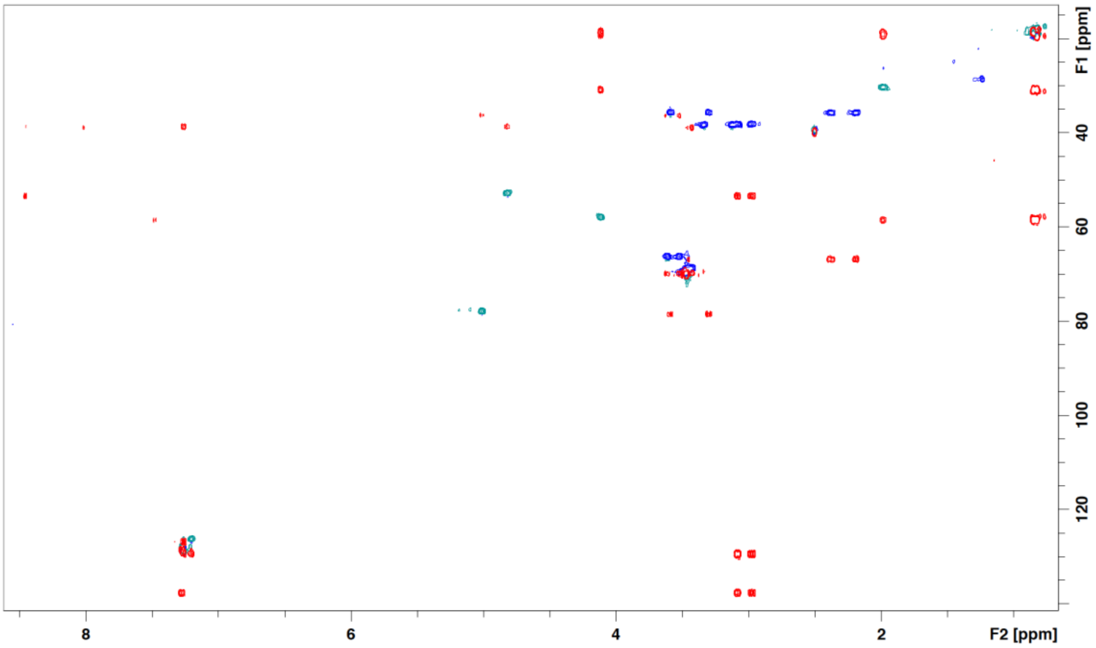

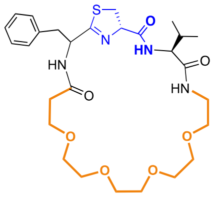

7b; Cyclo(-V(PEG)$_4$FThz-); DMSO-d6; 700 MHz; (20% of **6a**)

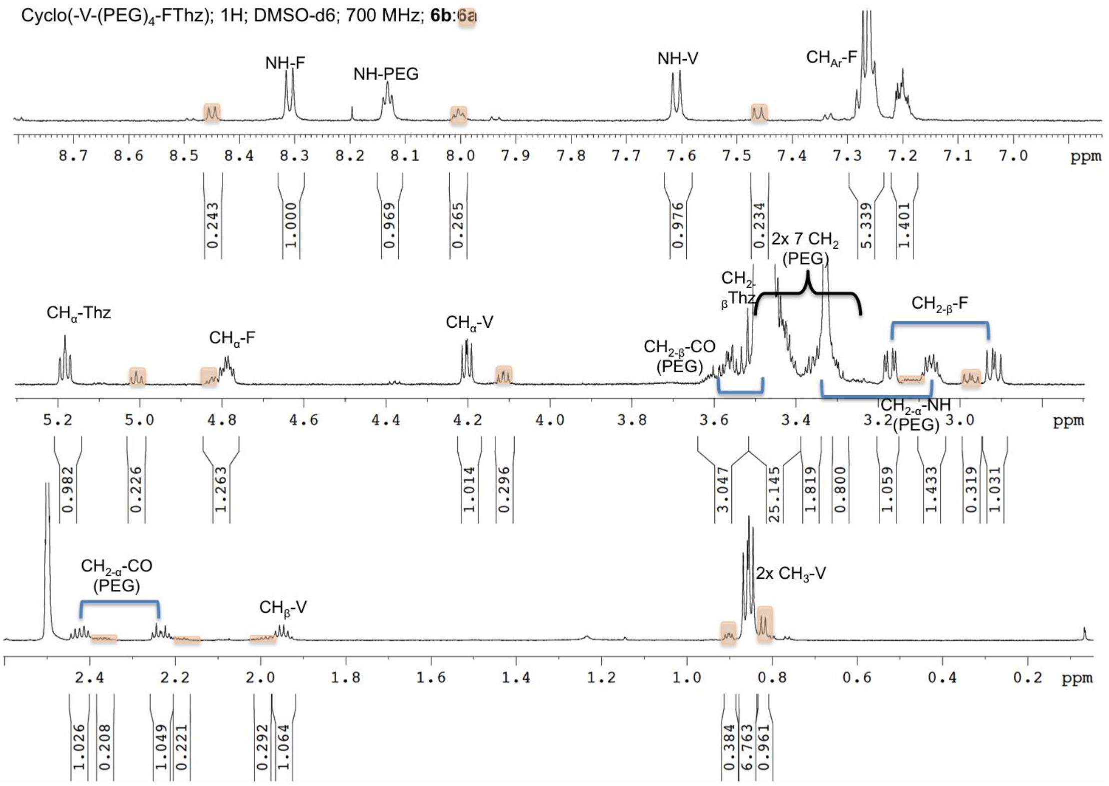

7b; Cyclo(-V(PEG)$_4$FThz-); HSQC (green/blue) and HMBC (red) spectra

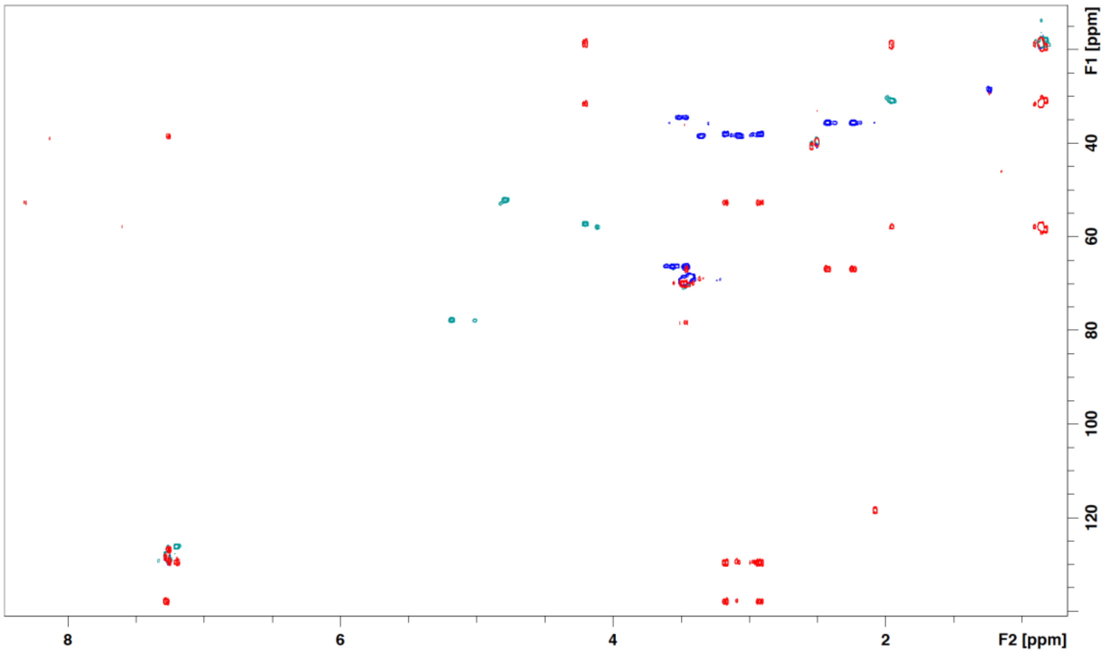

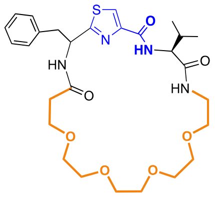

7c; Cyclo(-V(PEG)$_4$FTzl-); DMSO-d6; 700 MHz

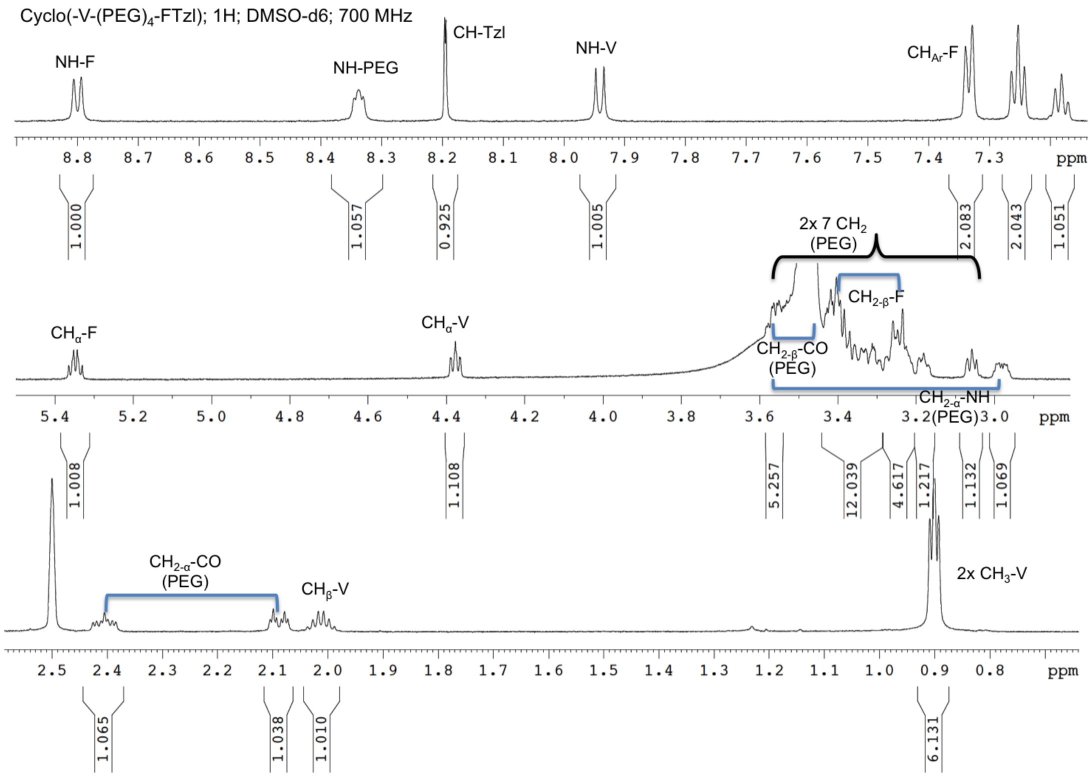

7c; Cyclo(-V(PEG)$_4$FTzl-); HSQC (green/blue) and HMBC (red) spectra

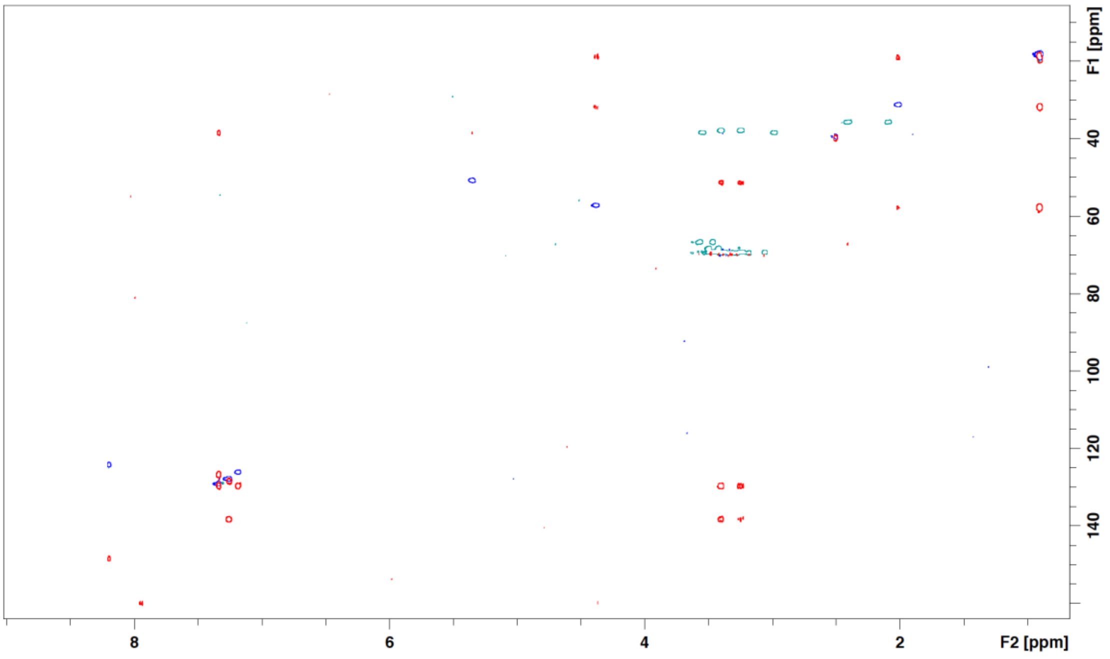

IX. EXSY NMR of final cyclic peptides

3; Cyclo(-VG$_a$AG$_b$-3-Abz-G$_c$FP-) 2:1; EXSY

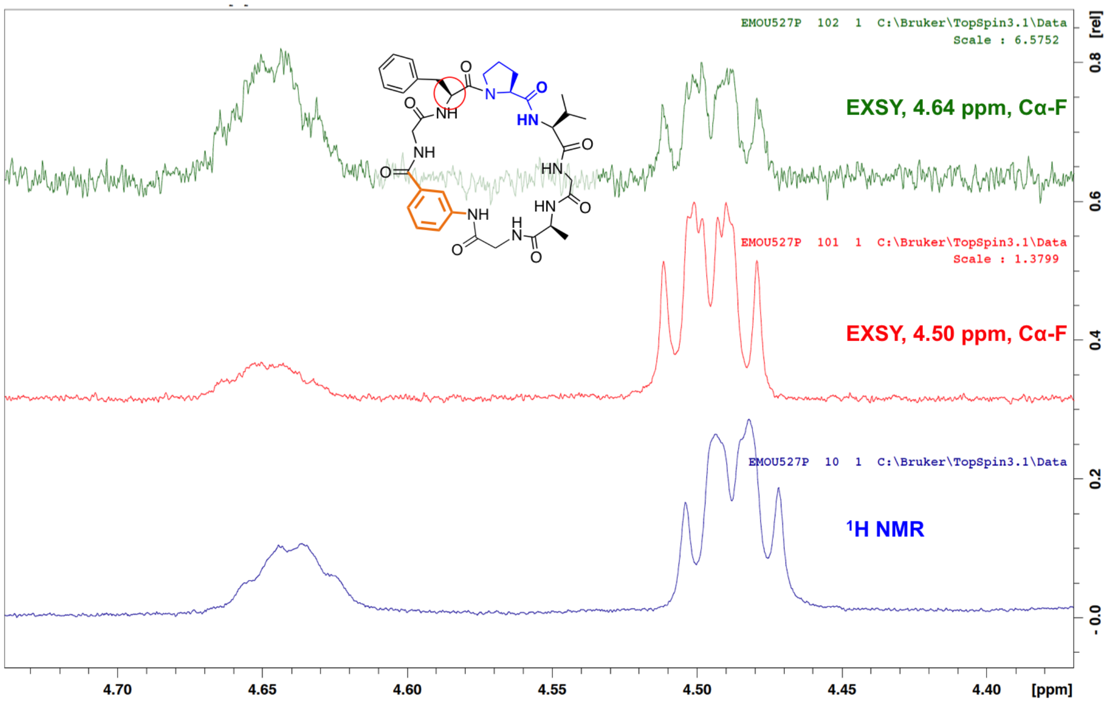

4; Cyclo(-VG$_a$A-Rib-IG$_c$FP -) 2:1; EXSY

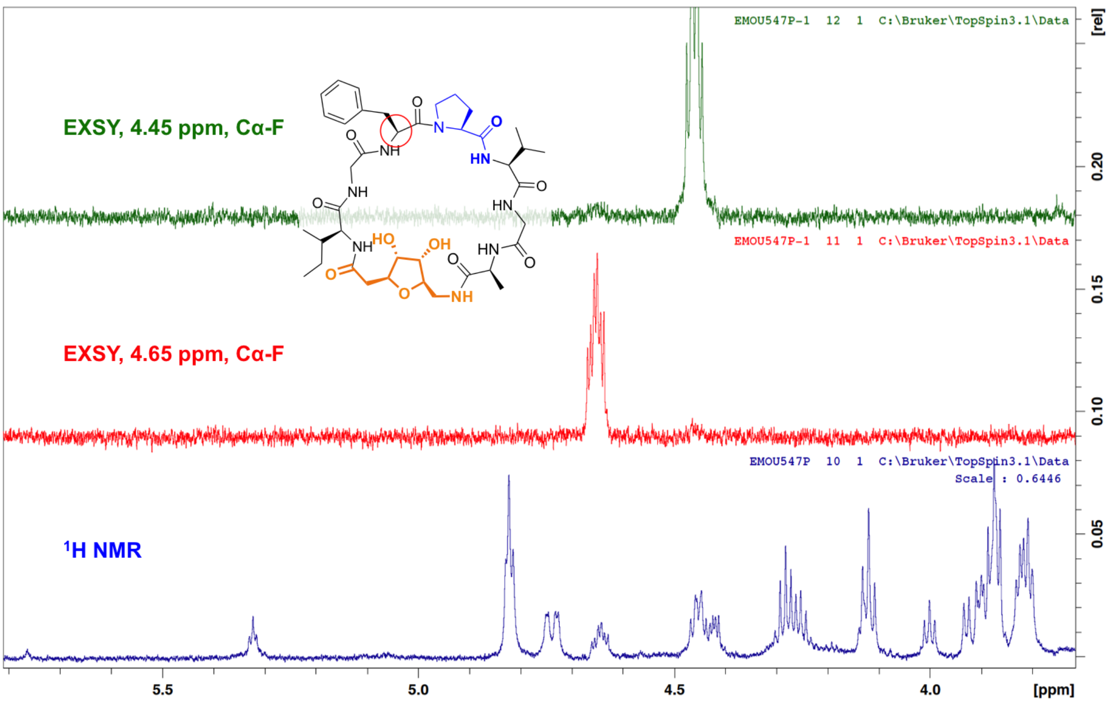

5; Cyclo(-V-8Aoc-8Aoc-FP-) 1.4:1; EXSY

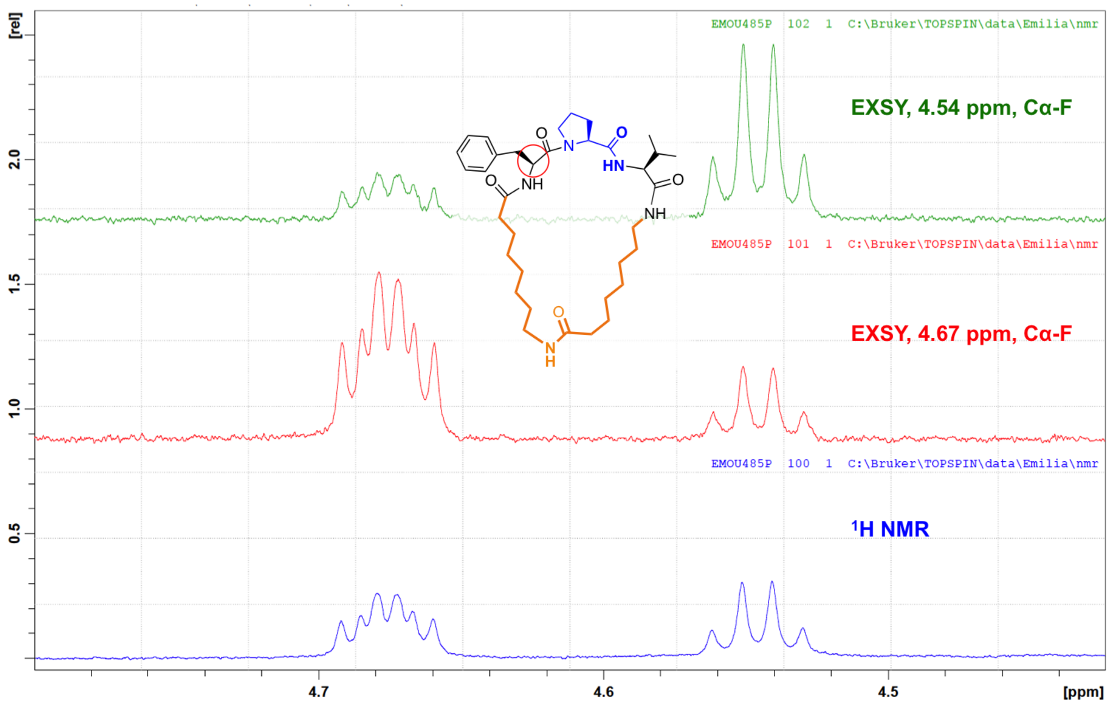

6; Cyclo(-V(PEG)$_4$FP-) 1.5:1; EXSY

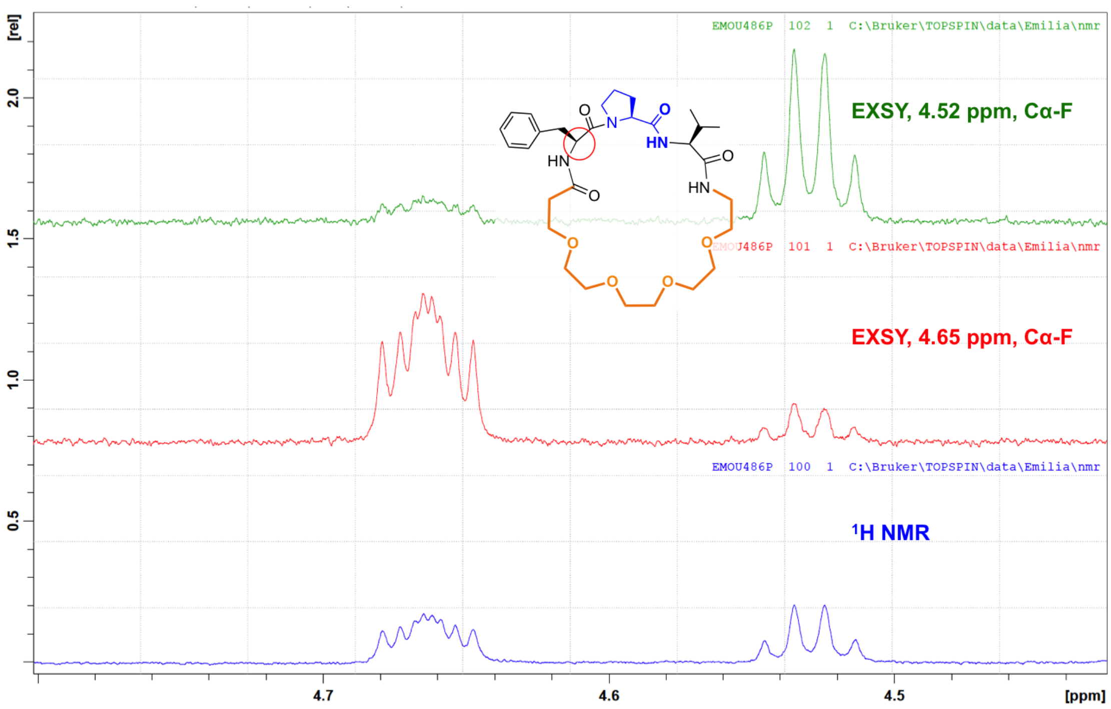

7b; Cyclo(-V(PEG)$_4$FThz-); EXSY

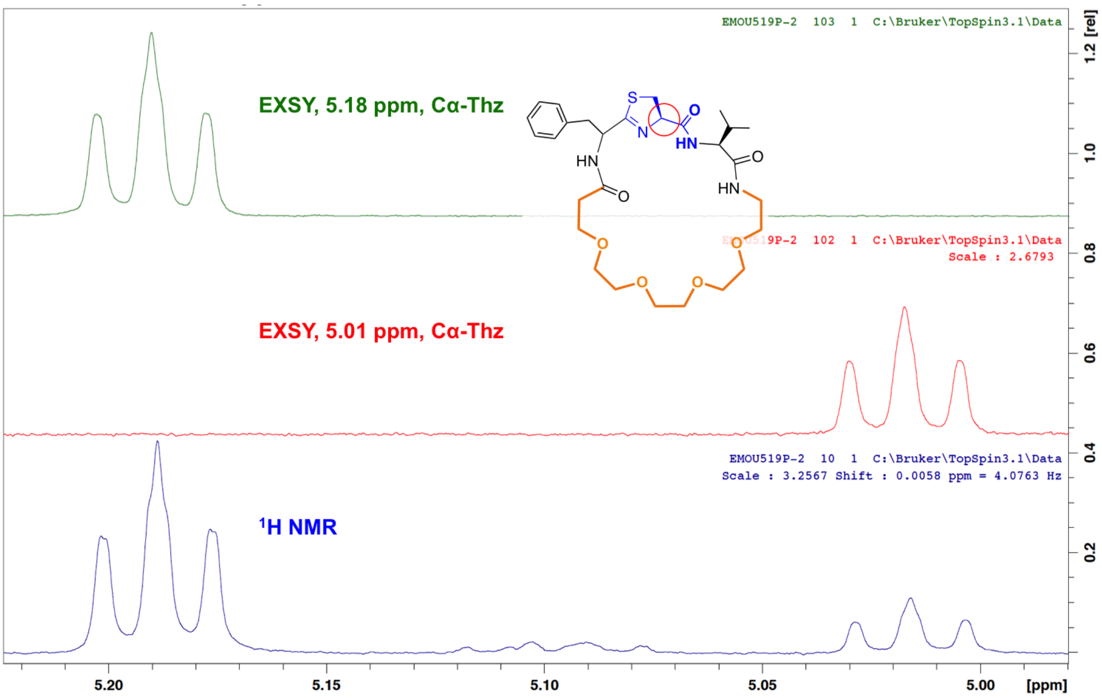

X. NMR spectra of sugar analogues intermediates

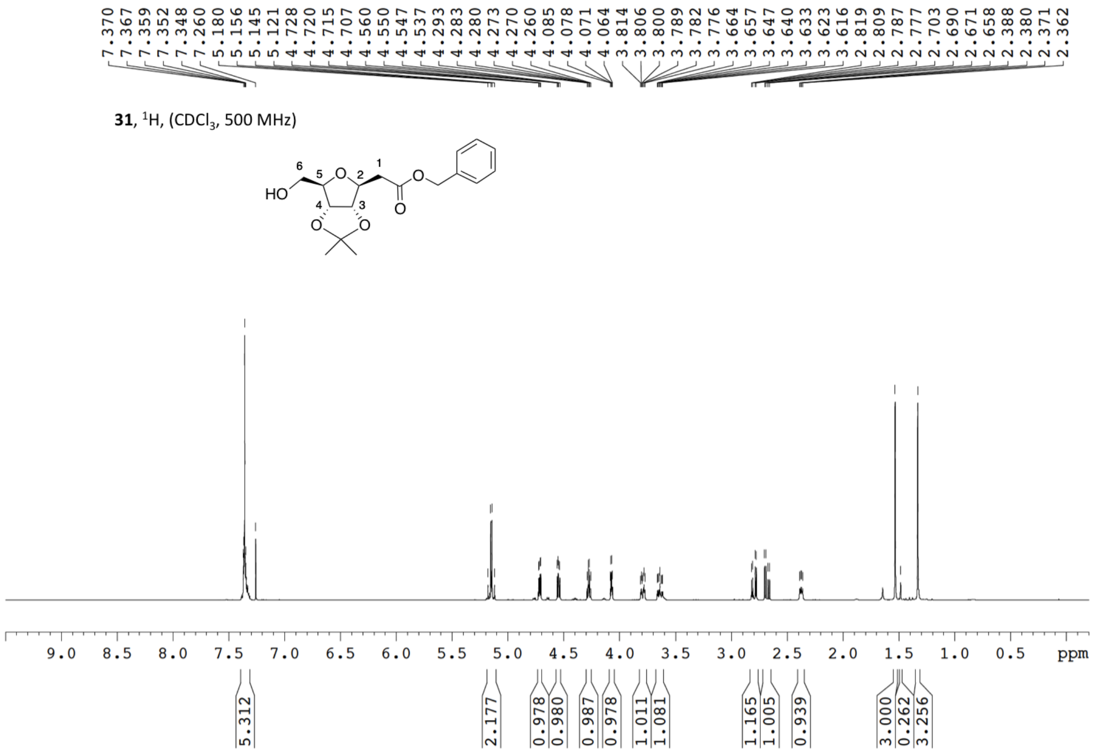

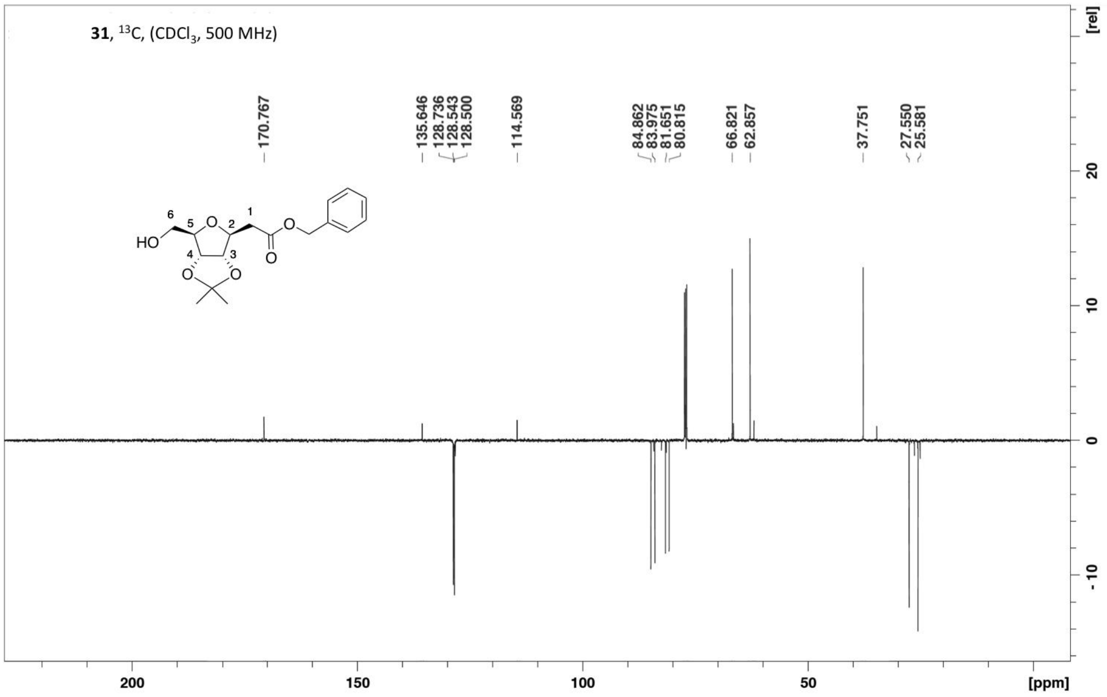

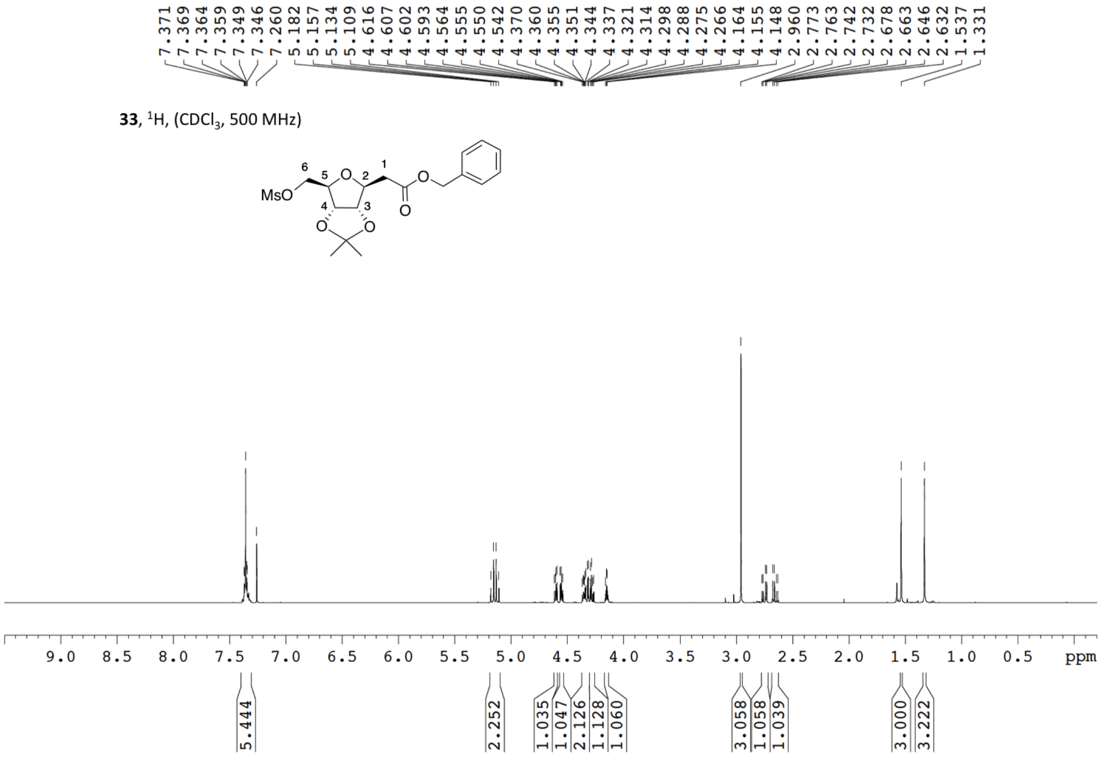
7.371
7.369
7.364
7.359
7.349
7.346
7.260
5.182
5.157
5.134
5.109
4.616
4.607
4.602
4.593
4.564
4.555
4.550
4.542
4.370
4.360
4.355
4.351
4.344
4.337
4.321
4.314
4.298
4.288
4.275
4.266
4.164
4.155
4.148
2.960
2.773
2.763
2.742
2.732
2.678
2.663
2.646
2.632
1.537
1.331
33, 1H, (CDCl3, 500 MHz)
MsO
6
5
4
3
2
1
O
ppm
5.444
2.252
1.035
1.047
2.126
1.128
1.060
3.058
1.058
1.039
3.000
3.222

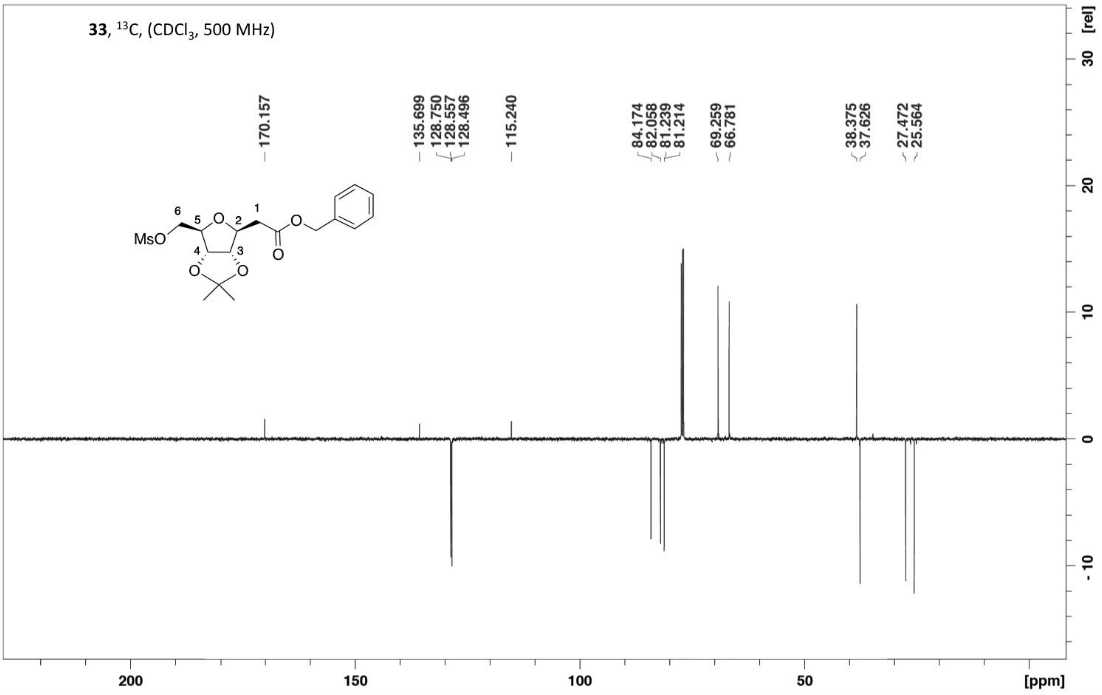
33, 13C, (CDCl3, 500 MHz)
170.157
135.699
128.750
128.557
128.496
115.240
84.174
82.058
81.239
81.214
69.259
66.781
38.375
37.626
27.472
25.564
MsO
6
5
4
3
2
1
O
[rel]
[ppm]

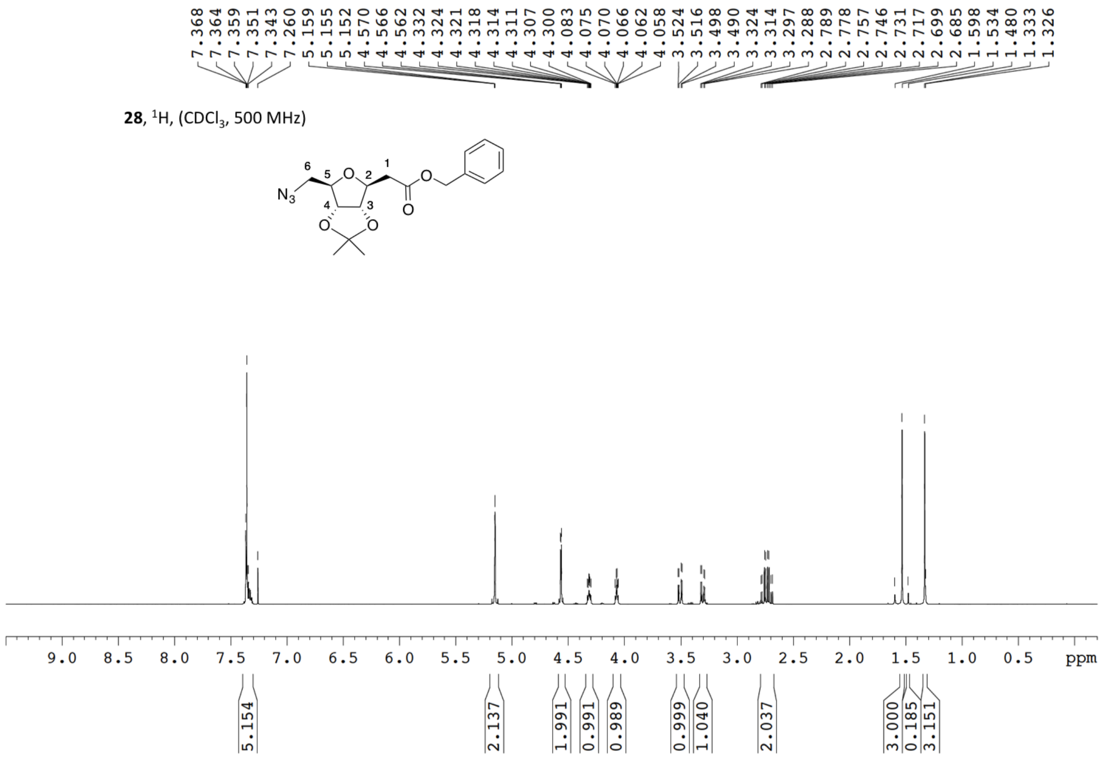
7.368
7.364
7.359
7.351
7.343
7.260
5.159
5.155
5.152
4.570
4.566
4.562
4.332
4.324
4.321
4.318
4.314
4.311
4.307
4.300
4.083
4.075
4.070
4.066
4.062
4.058
3.524
3.516
3.498
3.490
3.324
3.314
3.297
3.288
2.789
2.778
2.757
2.746
2.731
2.717
2.699
2.685
1.598
1.534
1.480
1.333
1.326
28, ¹H, (CDCl₃, 500 MHz)
ppm
5.154
2.137
1.991
0.991
0.989
0.999
1.040
2.037
3.000
0.185
3.151

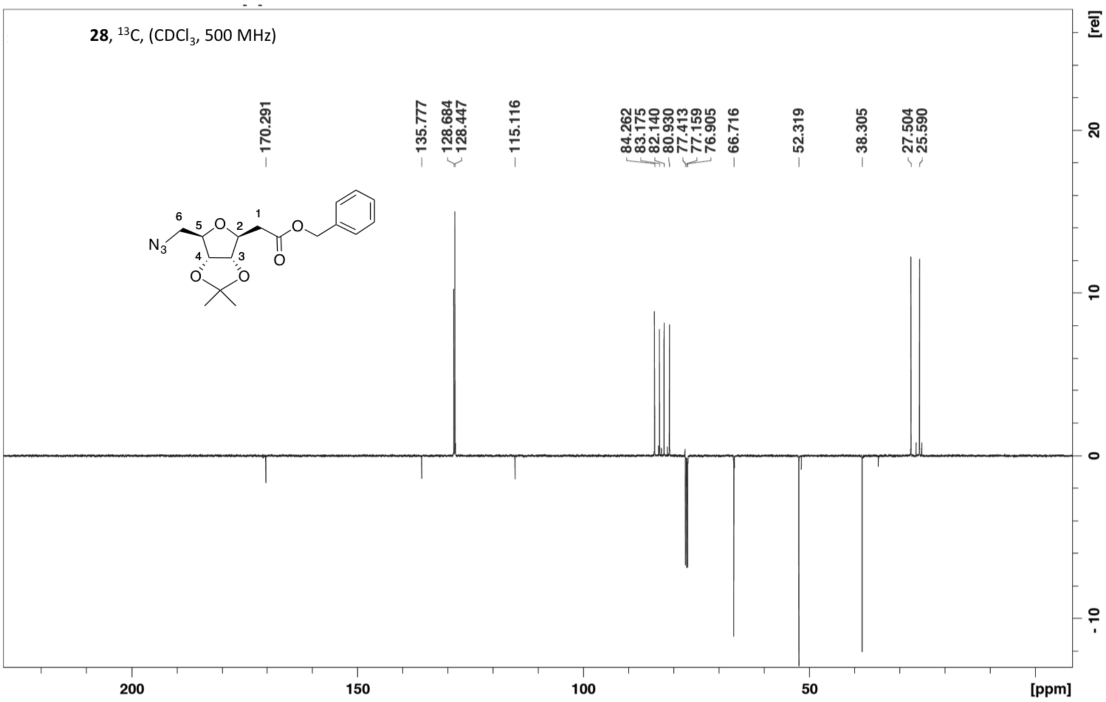
28, ¹³C, (CDCl₃, 500 MHz)
170.291
135.777
128.684
128.447
115.116
84.262
83.175
82.140
80.930
77.413
77.159
76.905
66.716
52.319
38.305
27.504
25.590
[rel]
[ppm]

29, ^{1}H, (CDCl$_3$, 500 MHz)

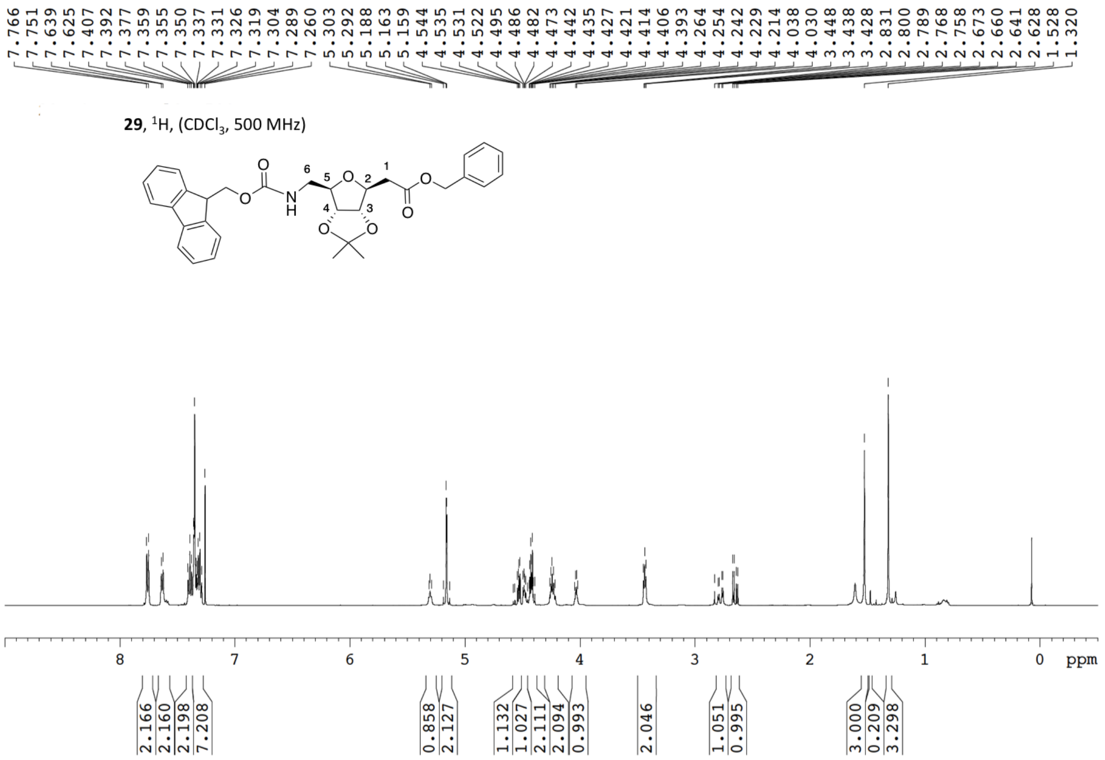

29, ^{13}C, (CDCl$_3$, 500 MHz)

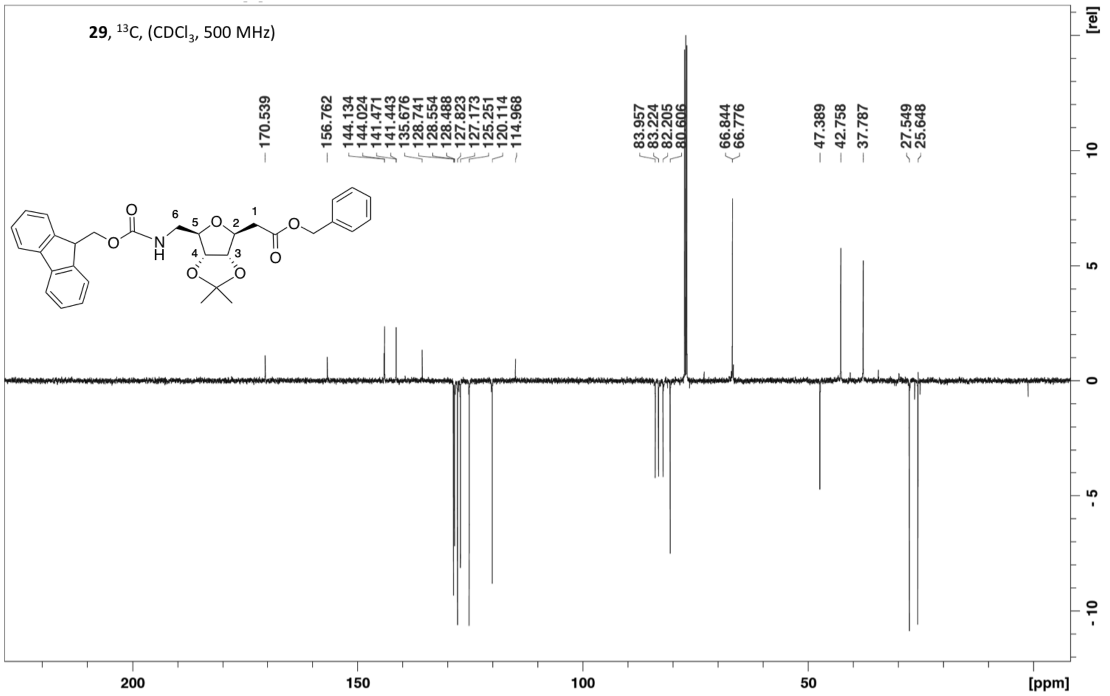

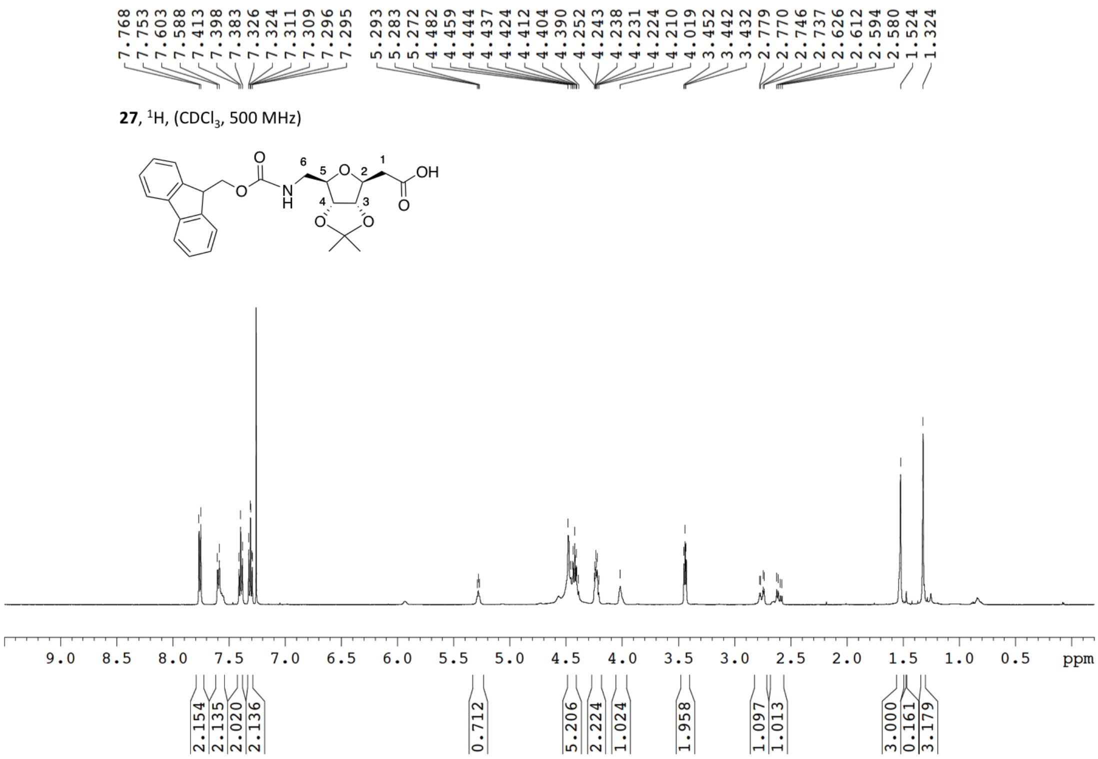
27, ¹H, (CDCl₃, 500 MHz)
7.768 7.753 7.603 7.588 7.413 7.398 7.383 7.326 7.324 7.311 7.309 7.296 7.295
5.293 5.283 5.272 4.482 4.459 4.444 4.437 4.424 4.412 4.404 4.390 4.252 4.243 4.238 4.231 4.224 4.210 4.019 3.452 3.442 3.432 2.779 2.770 2.746 2.737 2.626 2.612 2.594 2.580 1.524 1.324
9.0 8.5 8.0 7.5 7.0 6.5 6.0 5.5 5.0 4.5 4.0 3.5 3.0 2.5 2.0 1.5 1.0 0.5 ppm
2.154 2.135 2.020 2.136 0.712 5.206 2.224 1.024 1.958 1.097 1.013 3.000 0.161 3.179

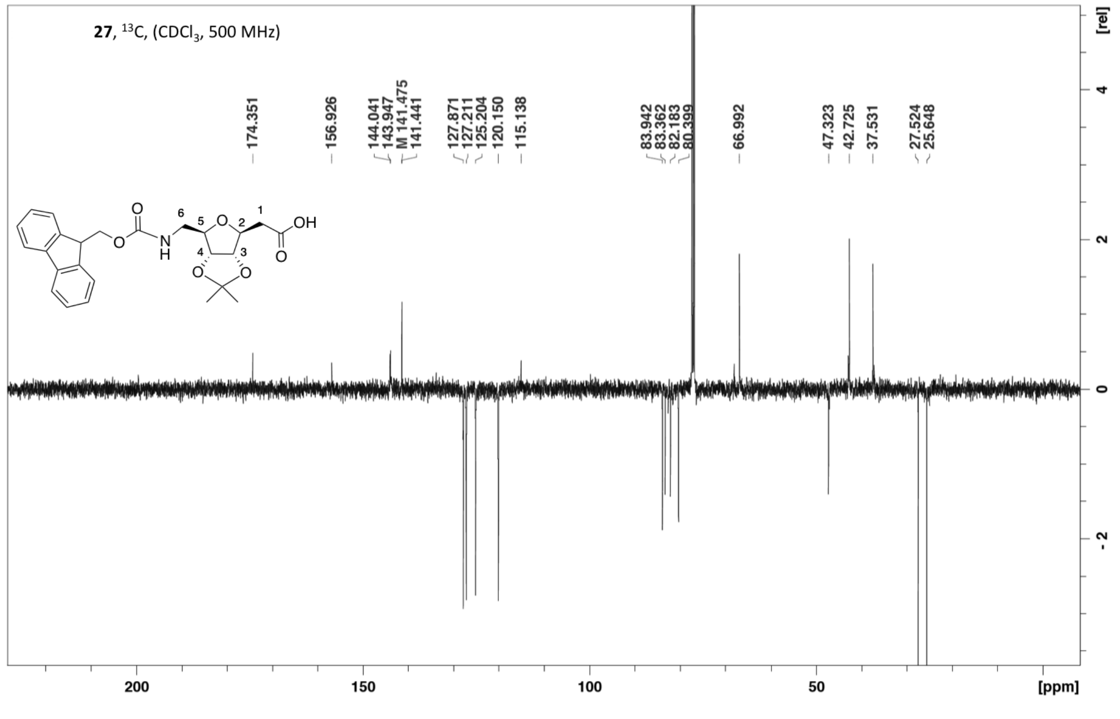
27, ¹³C, (CDCl₃, 500 MHz)
174.351 156.926 144.041 143.947 M 141.475 141.441 127.871 127.211 125.204 120.150 115.138 83.942 83.362 82.183 80.399 66.992 47.323 42.725 37.531 27.524 25.648
[rel]
200 150 100 50 [ppm]

XI. MS-MS data of enzymatic reaction products of peptides 8-26

MS-MS fragmentation data of PatGmac products were acquired on the expected mass. The corresponding fragments and their theoretical masses are shown.

Cyclic peptides fragmentation pattern: The fragmentation can start at any point of the macrocycle Fragments containing both the *N*-terminal Val and the *C*-terminal Pro can only exist in the fragmentation pattern of cyclic peptide. CO loss is more common in cyclic peptides.

Linear peptides fragmentation pattern: Fragmentation can only start at the *N*- or *C*-terminal of the peptide. Fragments with both the *N*-terminal Val and the *C*-terminal Pro won't exist. One or two H_2O molecules loss is more common in linear peptides.

PatGmac reaction product of 8: Cyclo(-VGA-**β-Ala**-IGFP-)

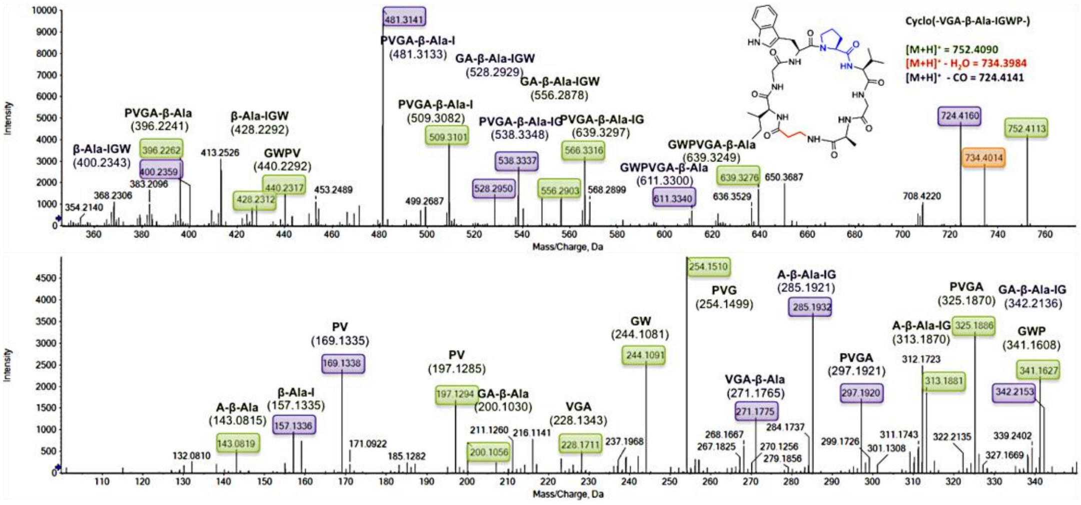

PatGmac reaction product of 9: Cyclo(-VGA-**GABA**-IGFP-)

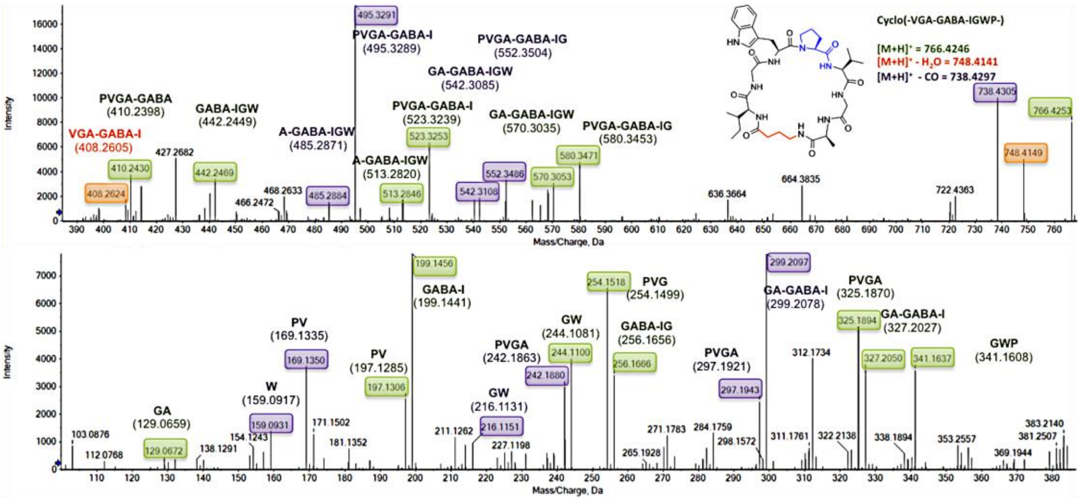

PatGmac reaction product of 10: Cyclo(-VGA-**Doc**-IGFP-)

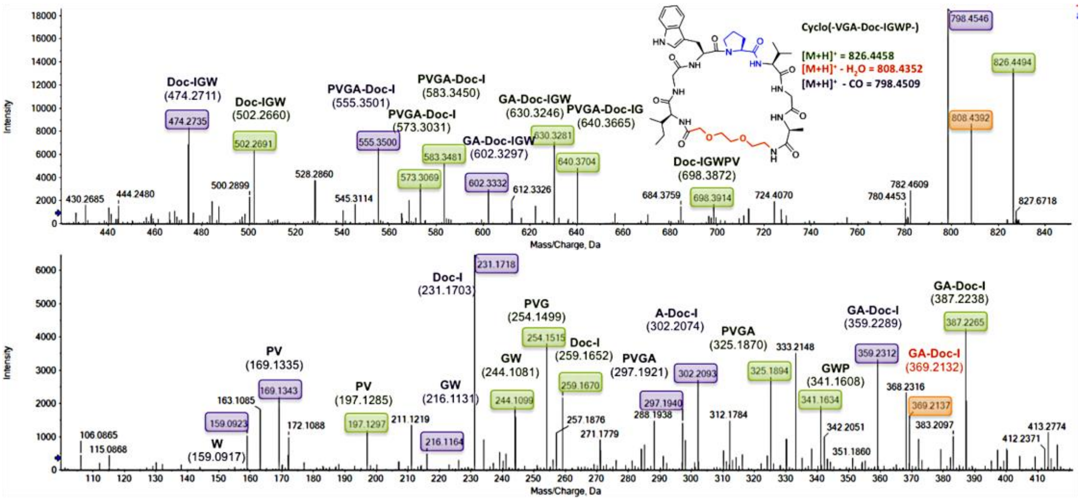

PatGmac reaction product of 11: Cyclo(-VG$_a$(*N*-Me)AG$_b$IG$_c$WP-)

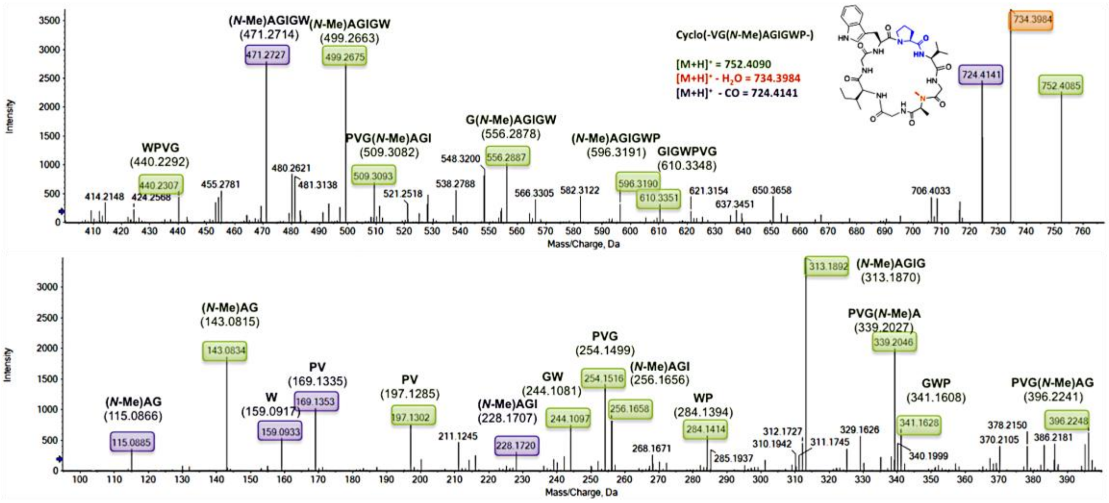

PatGmac reaction product of 12: Cyclo(-VGA-2-Abz-IGFP-)

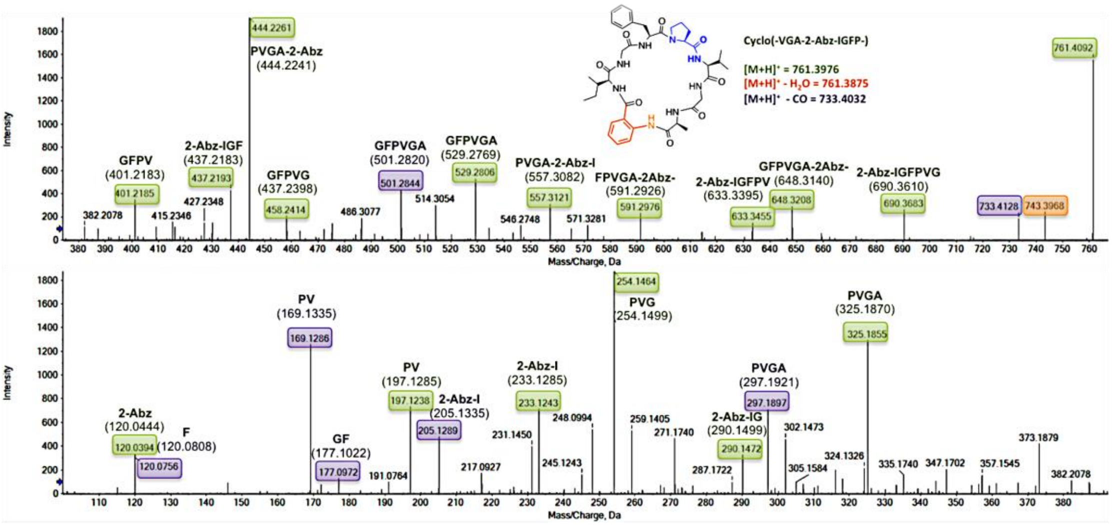

PatGmac reaction product of 13: Cyclo(-VGA-3-Abz-IGFP-)

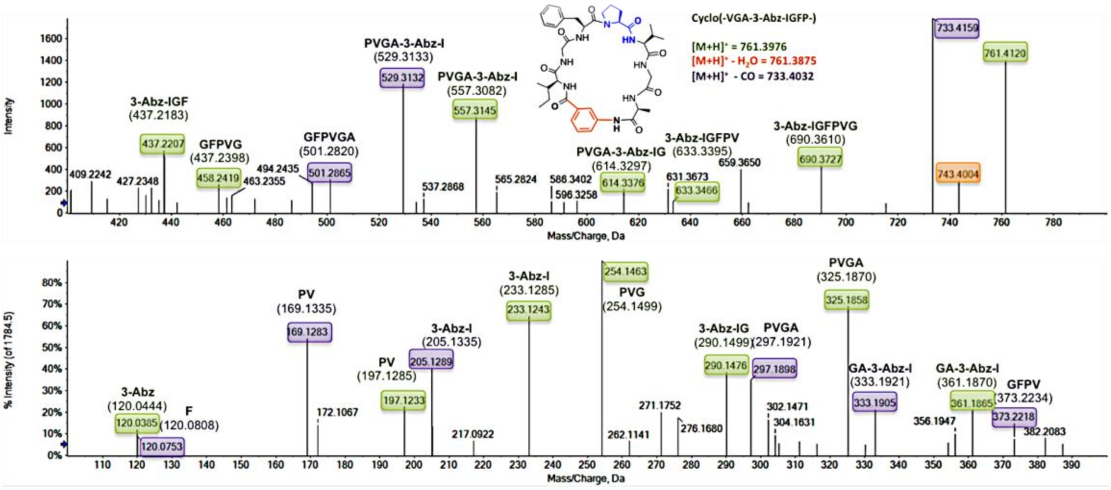

PatGmac reaction product of 14: Cyclo(-VGA-4-Abz-IGFP-); (**1**)

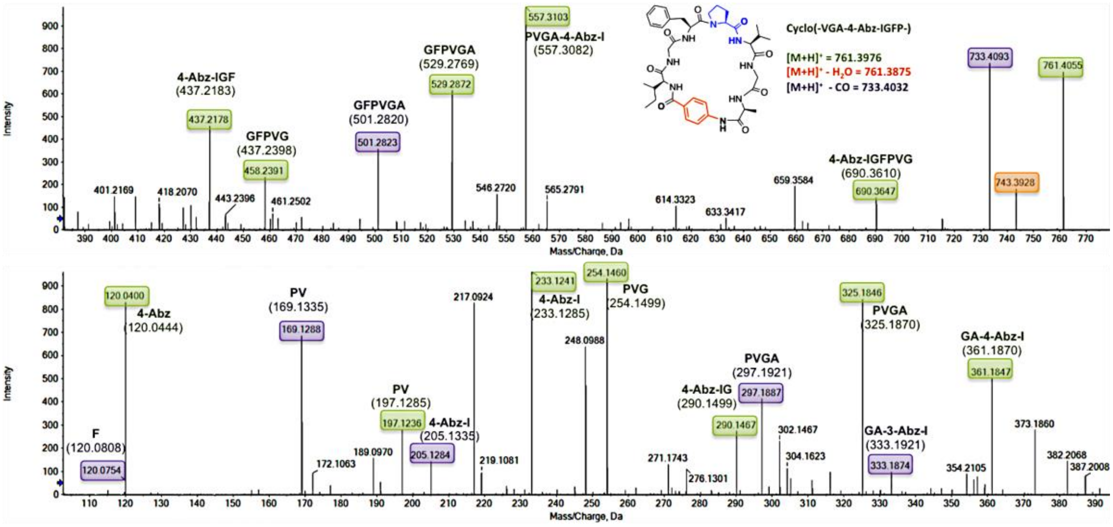

PatGmac reaction product of 15: Cyclo(-VGAG-2-Abz-GFP-)

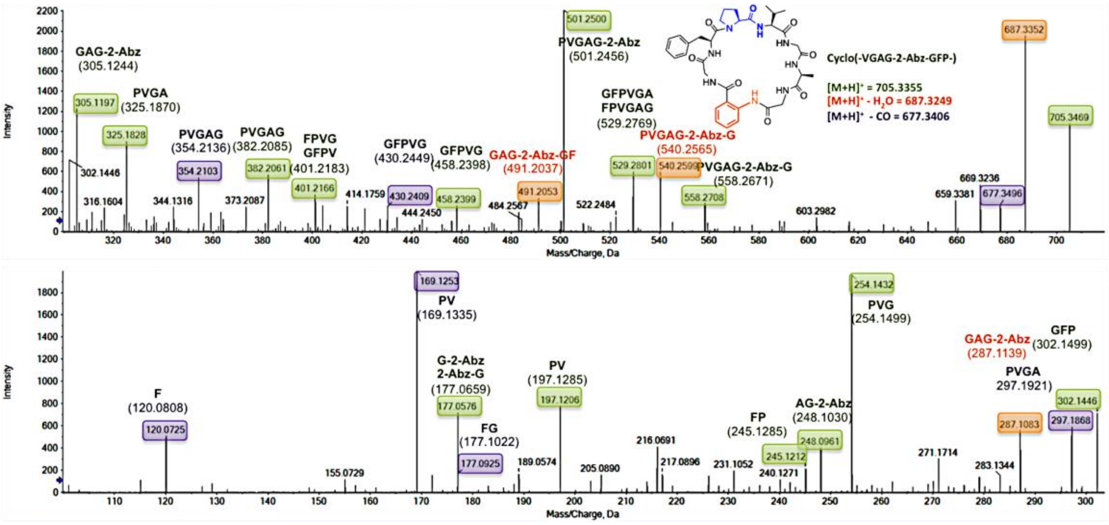

PatGmac reaction product of 16: Cyclo(-VGAG-3-Abz-GFP-); (**2**)

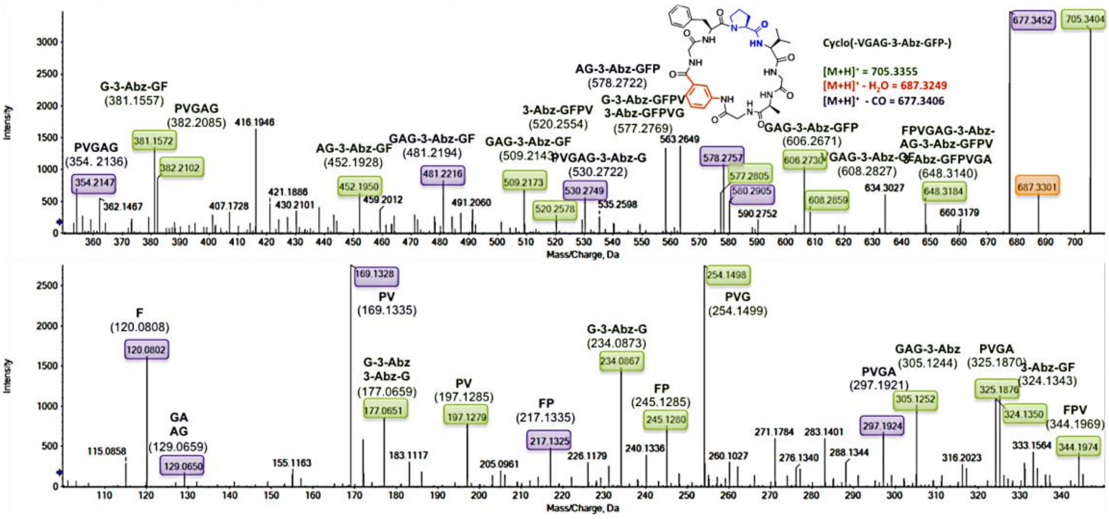

PatGmac reaction product of 17: Cyclo(-VGAG-4-Abz-GFP-)

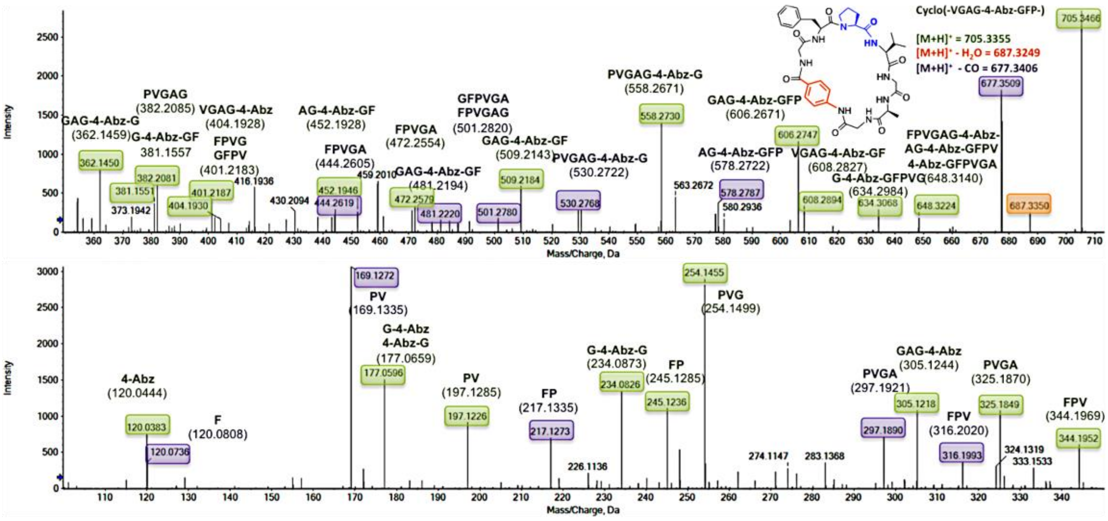

PatGmac reaction product of 18: Cyclo(-VGA-Rib-IGFP-); (**3**)

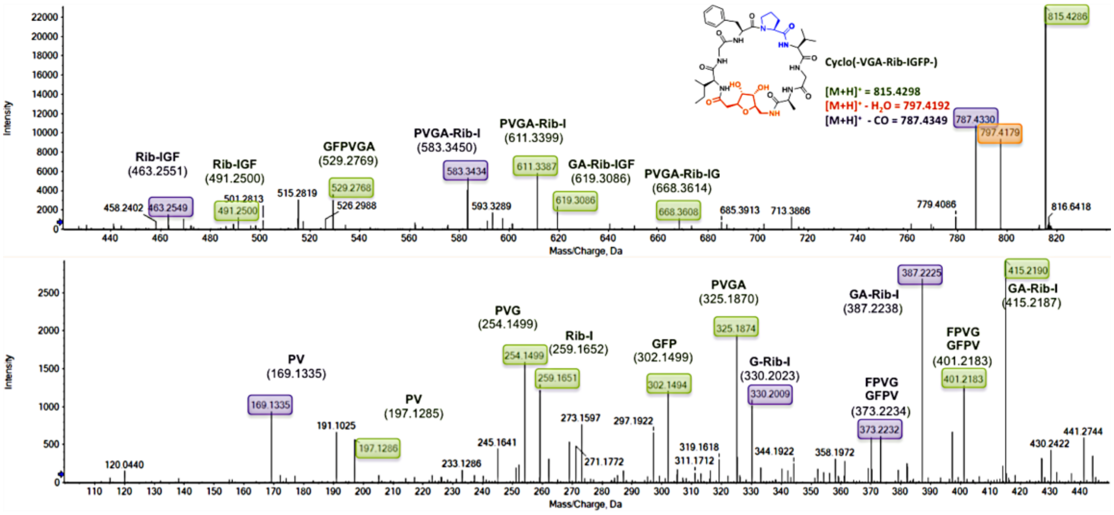

PatGmac reaction product of 19: 8Aoc-AGIGFP (Linear)

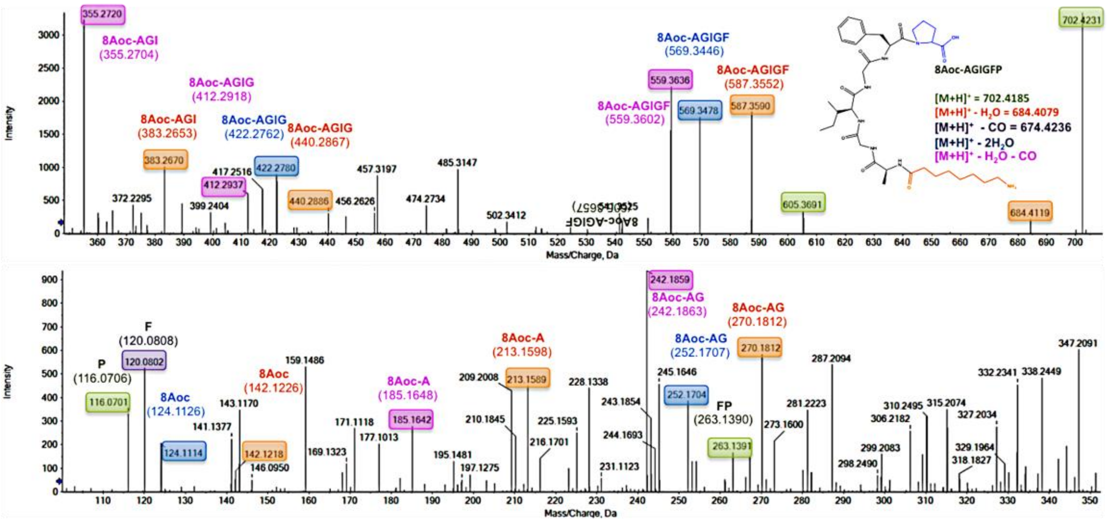

PatGmac reaction product of 20: Cyclo(-V-7Ahp-GAGFP-)

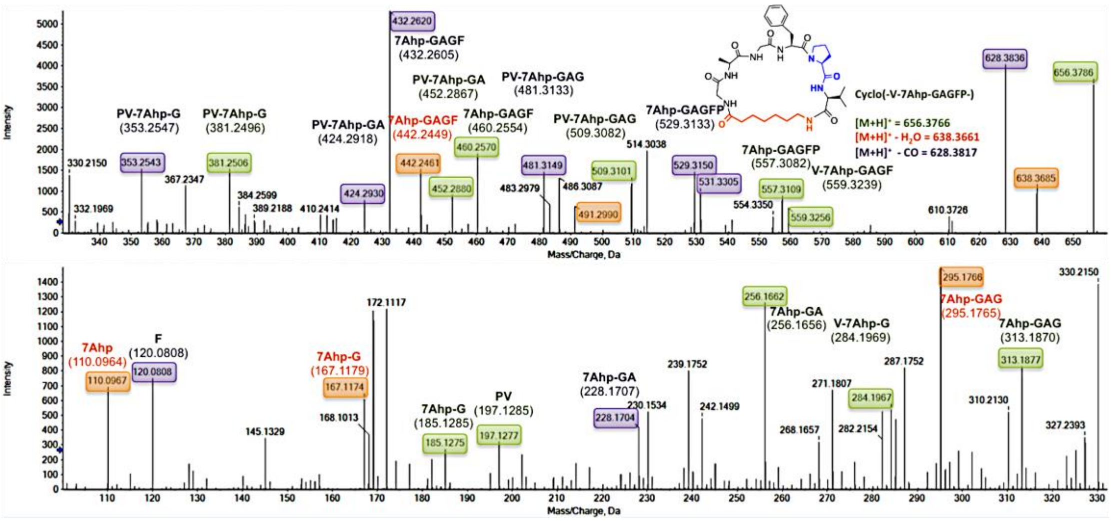

PatGmac reaction product of 22: Cyclo(-VGAG-8Aoc-FP-)

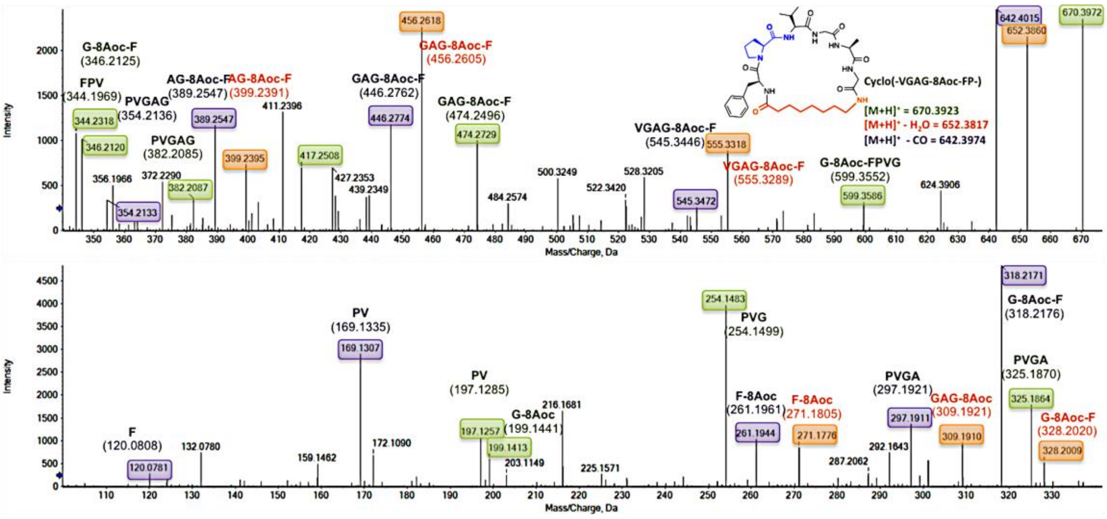

PatGmac reaction product of 23: V-8Aoc-FP (Linear)

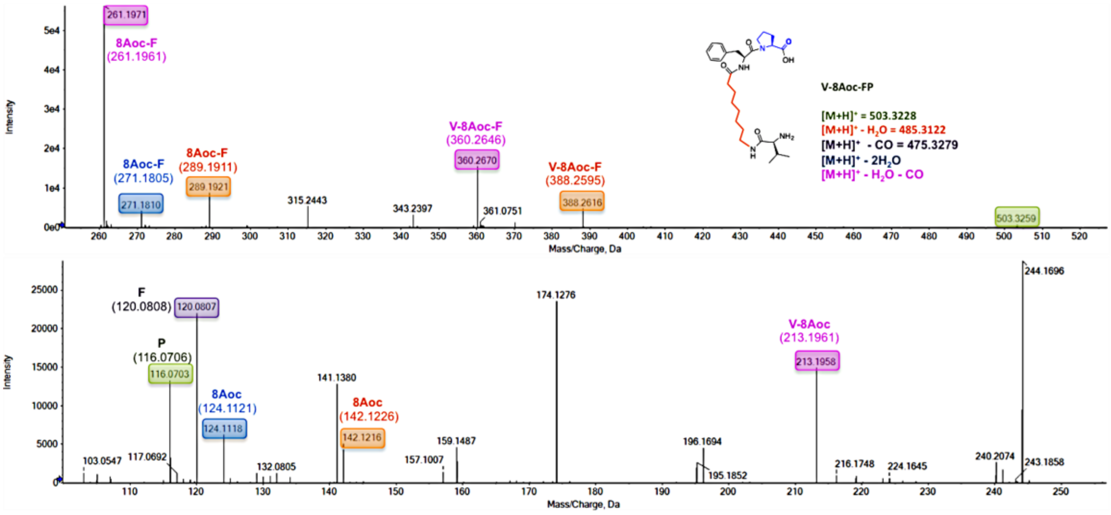

PatGmac reaction product of 24: Cyclo(-V-8Aoc-8Aoc-FP -); (**4**)

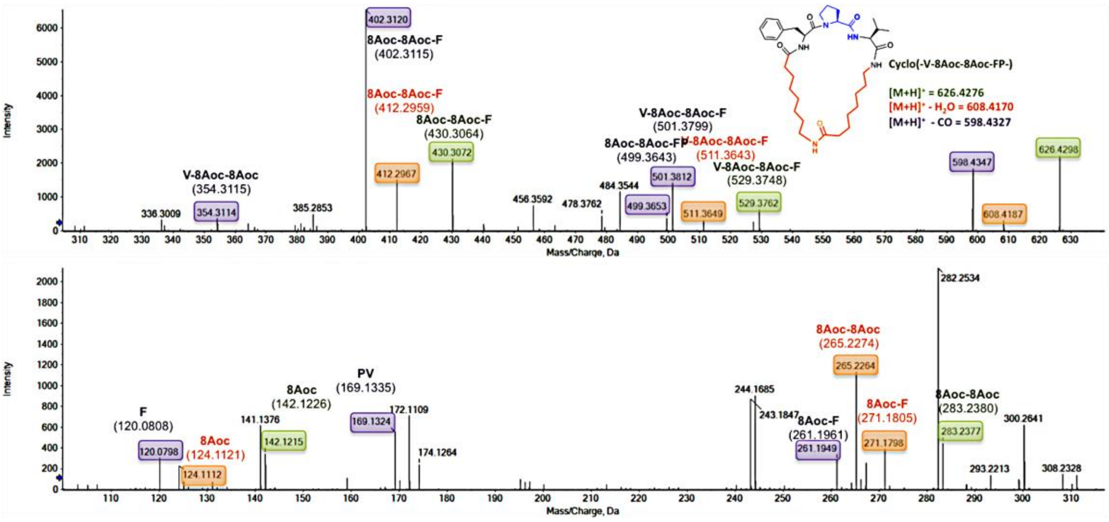

PatGmac reaction product of 25: Cyclo(-V(PEG)$_4$FP-); (**5**)

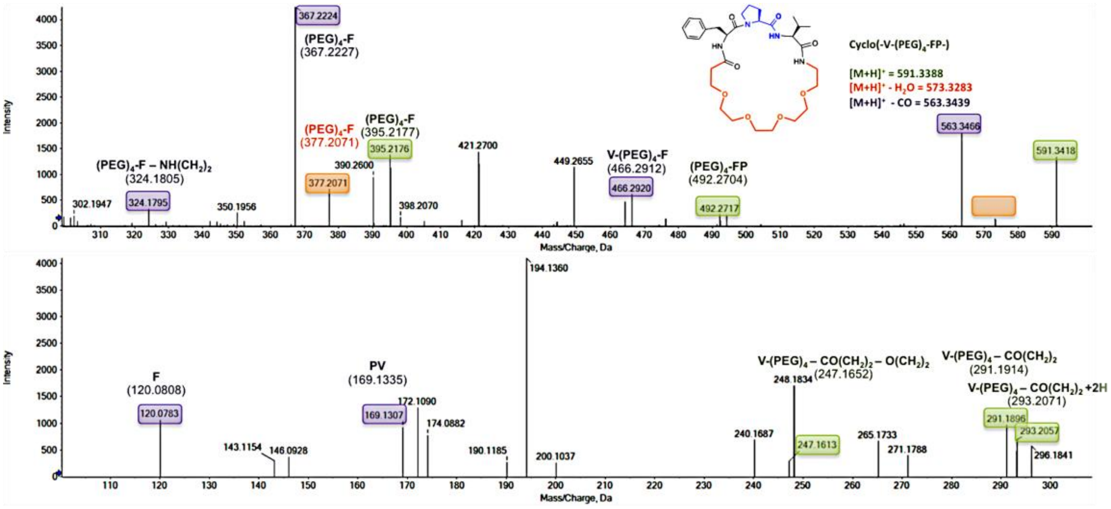

Fused LynD reaction product of 26; V(PEG)$_4$FThzAYD-NH$_2$

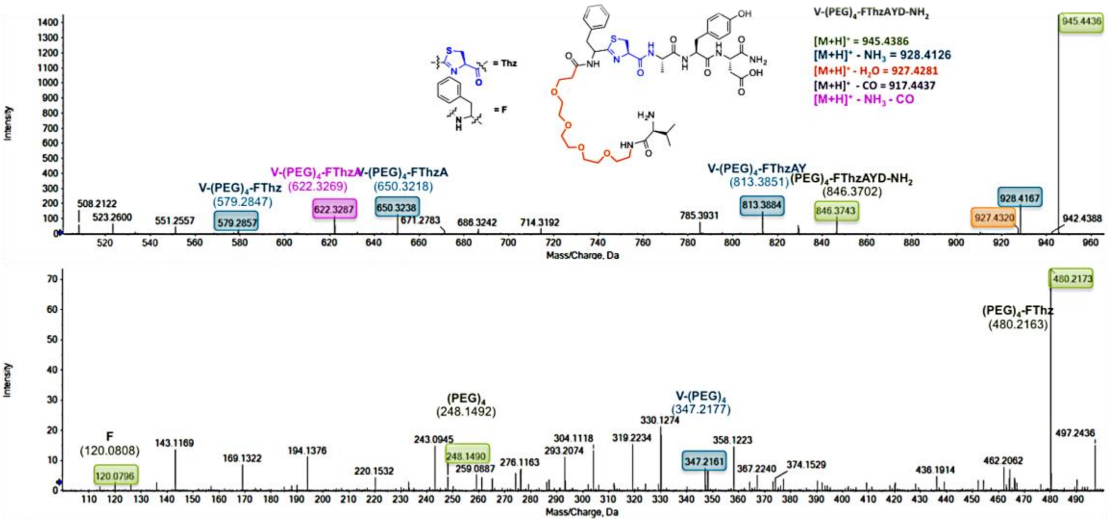

PatGmac reaction product of 26 (after LynD reaction); Cyclo(-V(PEG)4FThz-); (**7a, 7b**)

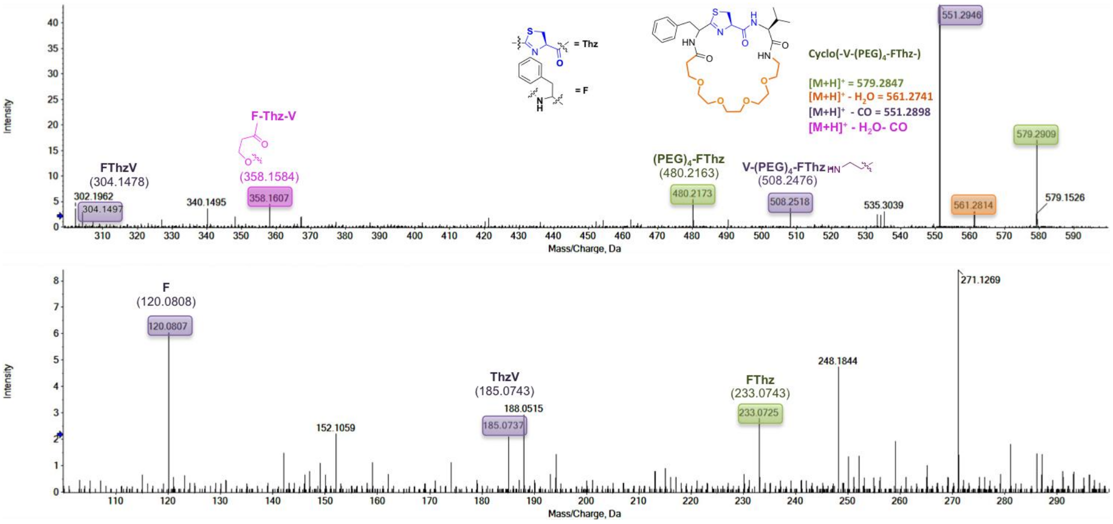

ArtGox reaction product of 7a; Cyclo(-V(PEG)4FTzl-); (**7c**)

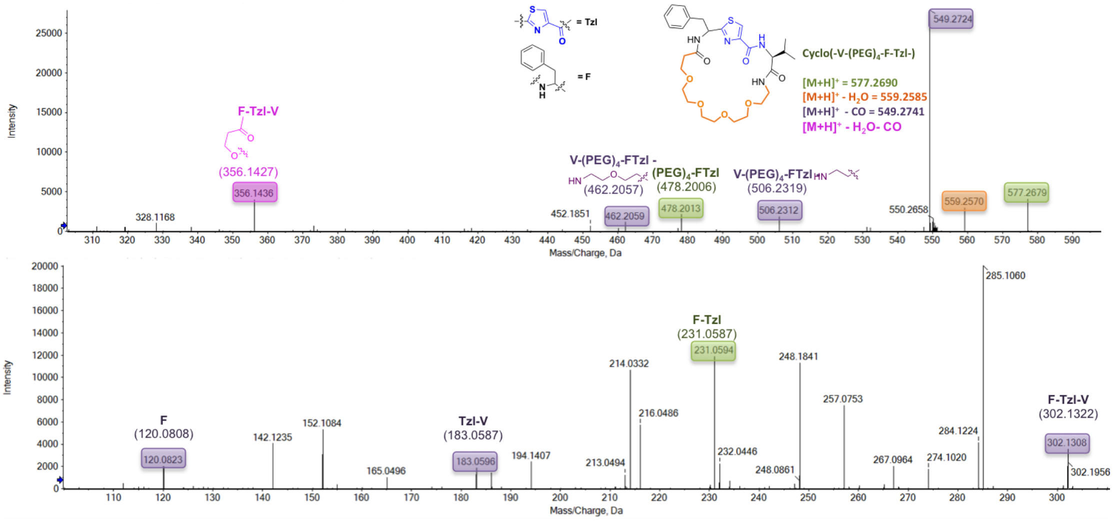

XII. Maldi-MS traces of the reactions not processed by PatGmac with starting hybrid peptides 21, 36-38

Table S3: MS data, retention time and HPLC purities of starting hybrid peptides 21, 36-38

	Peptide[a]	MS $[M+H]^+$	Rt[b]	Purity[c]
**21**	VGAG-**7Ahp**-PAYD	875.5	14.95	99%
**36**	V-**7Ahp-7Ahp**-PAYD	817.5	16.76	98%
**37**	**8Aoc-8Aoc**-PAYD	746.4	17.04	98%
**38**	V-**(PEG)$_4$**-PAYD	810.4	14.94	98%

[a] For structures of the non-natural amino acidic scaffolds, refer to section I.
[b] Retention time as observed by analytical HPLC following system A.
[c] Purity assessed by analytical HPLC at 220 nm UV absorption.

PatGmac reaction of 21 (VGAG-**7Ahp**-PAYD**)**

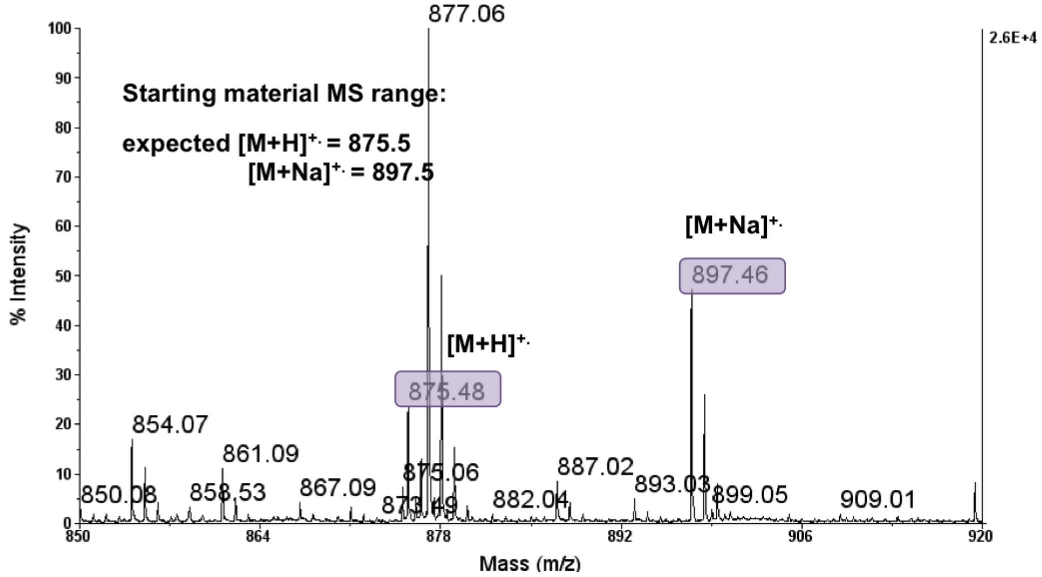

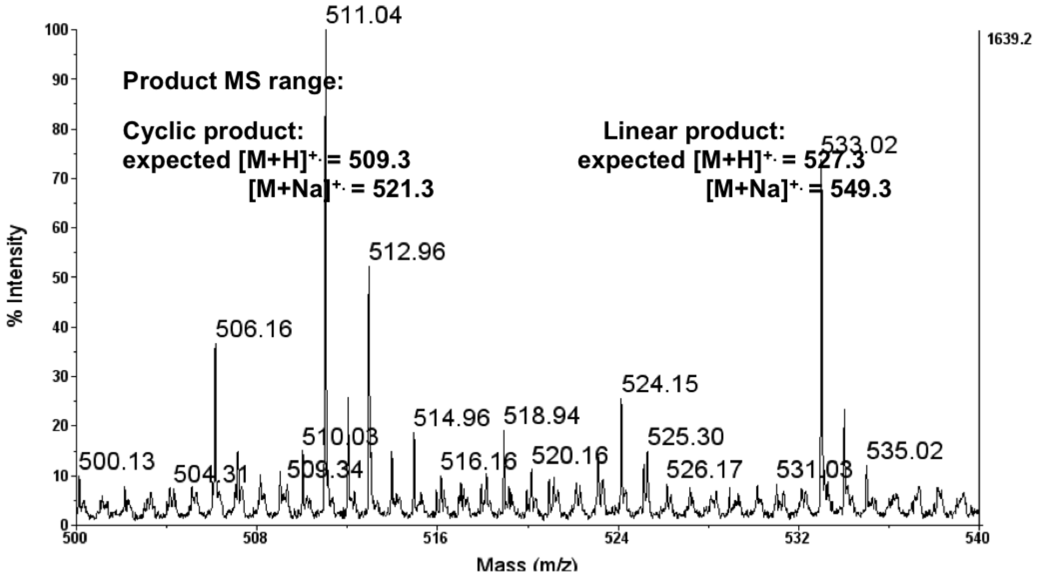

PatGmac reaction of 36 (V-7Ahp -7Ahp-PAYD)

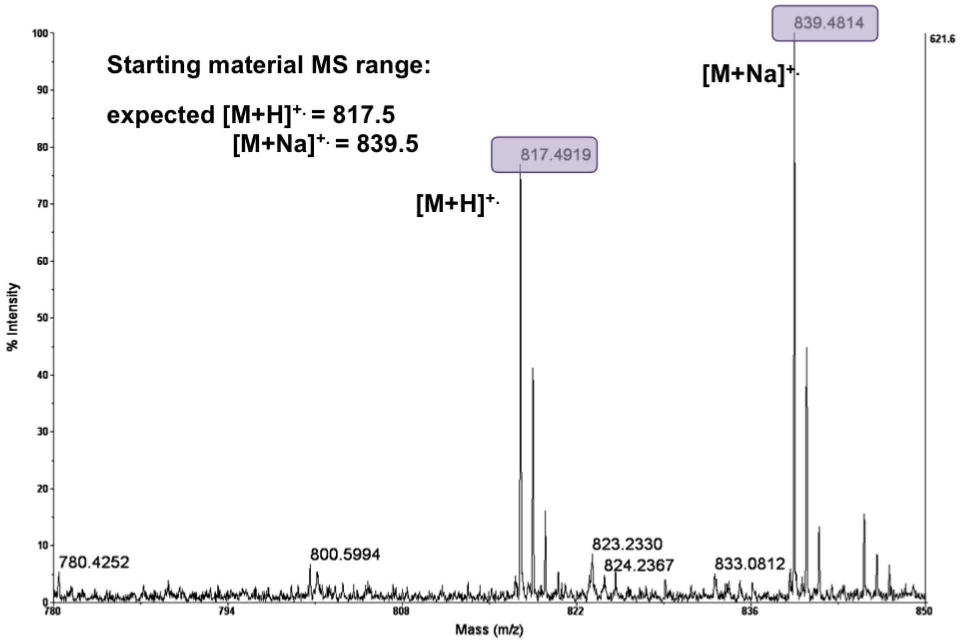

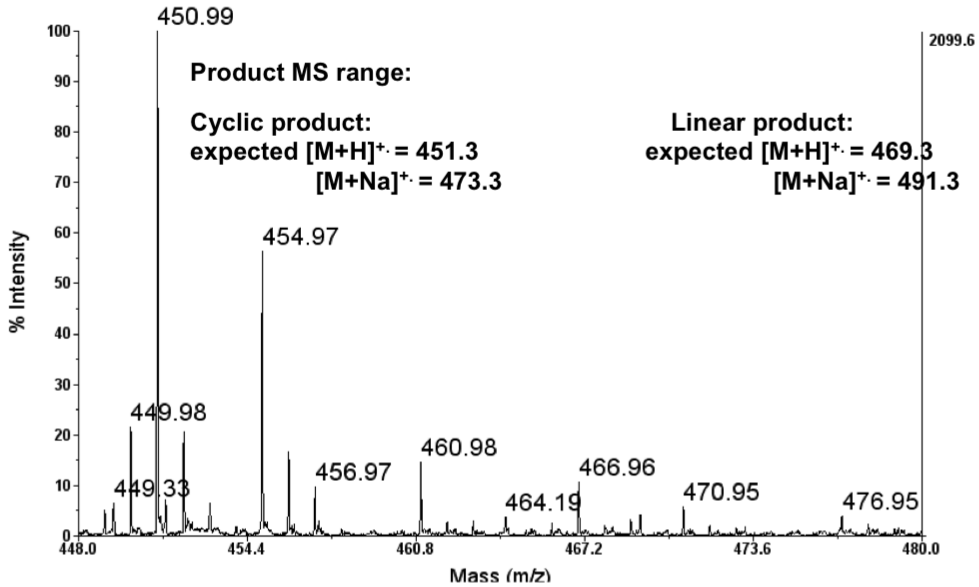

PatGmac reaction of 37 (8Aoc-8Aoc -PAYD)

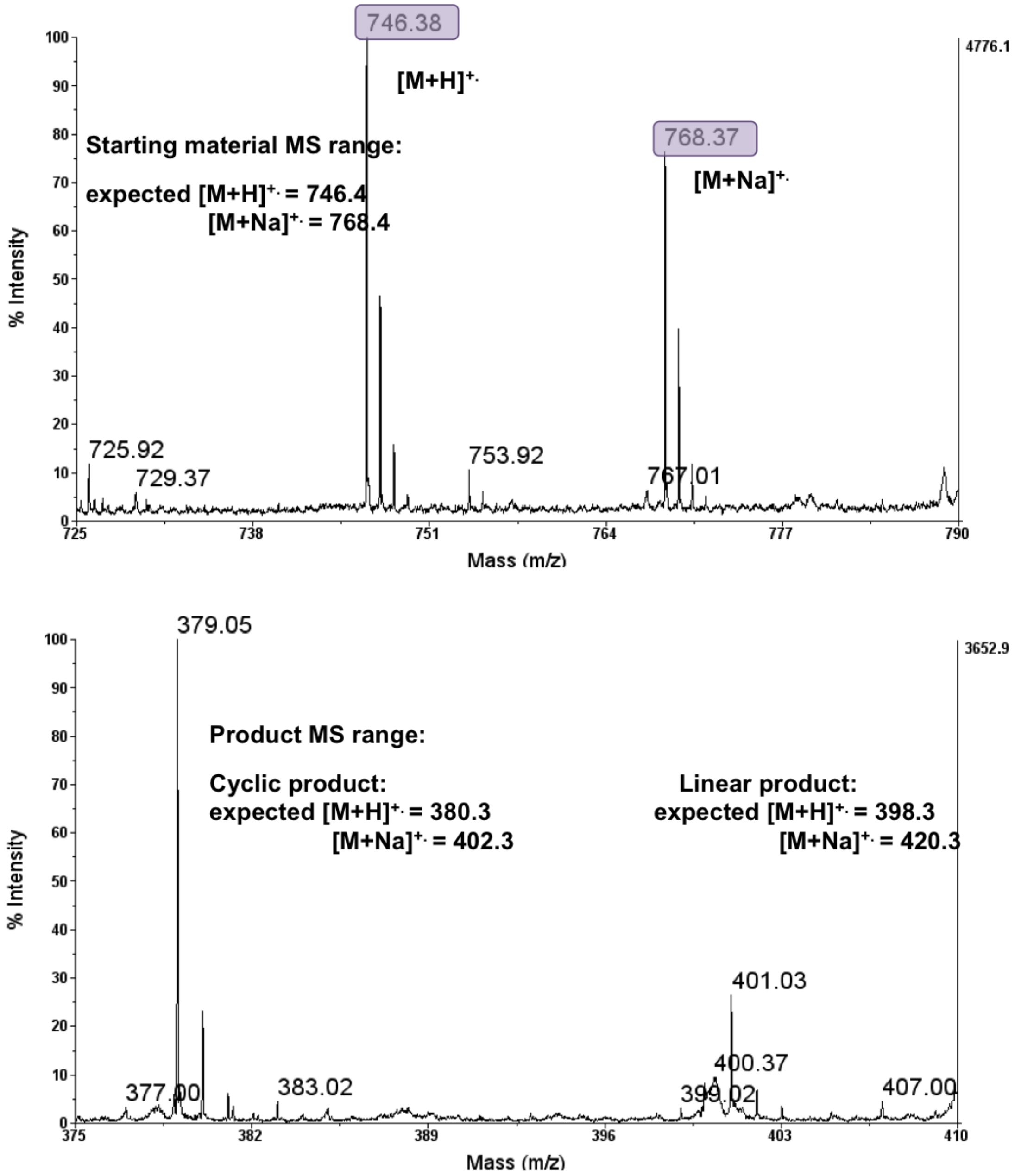

PatGmac reaction of 38 (V-(PEG)$_4$-PAYD)

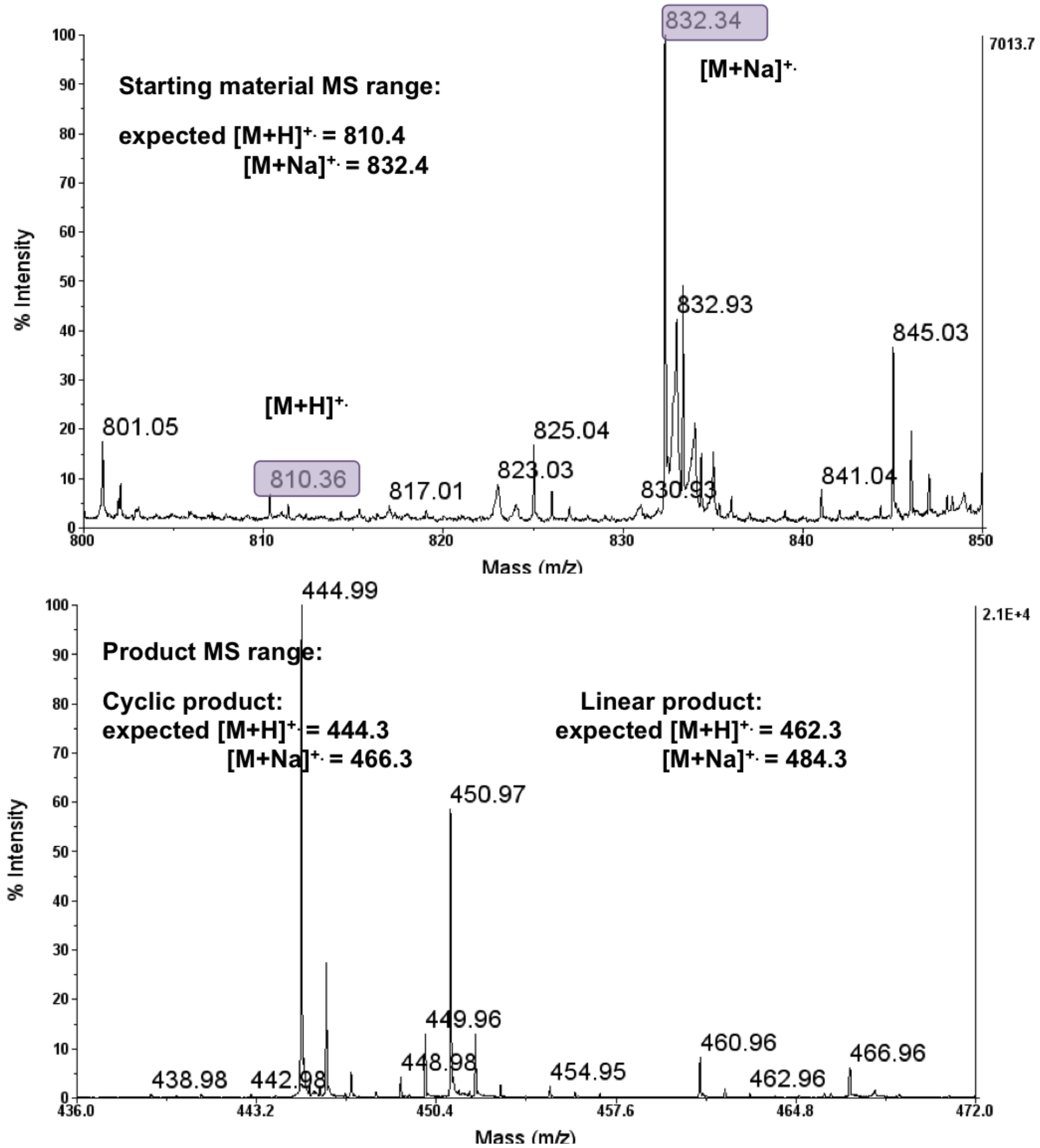

XIII. LC-MS traces of final cyclic peptides

For each of the cyclic hybrid peptides **1-7**, the UV trace at 220 nm obtained by HPLC is complemented by its corresponding LCMS trace at the desired molecular weight (Single Ion Monitoring SIM mode) The HPLC methods used are described in section II.

Cyclic peptide **1**; Cyclo(VG(***N*-Me**)AGIGFP-), system A1

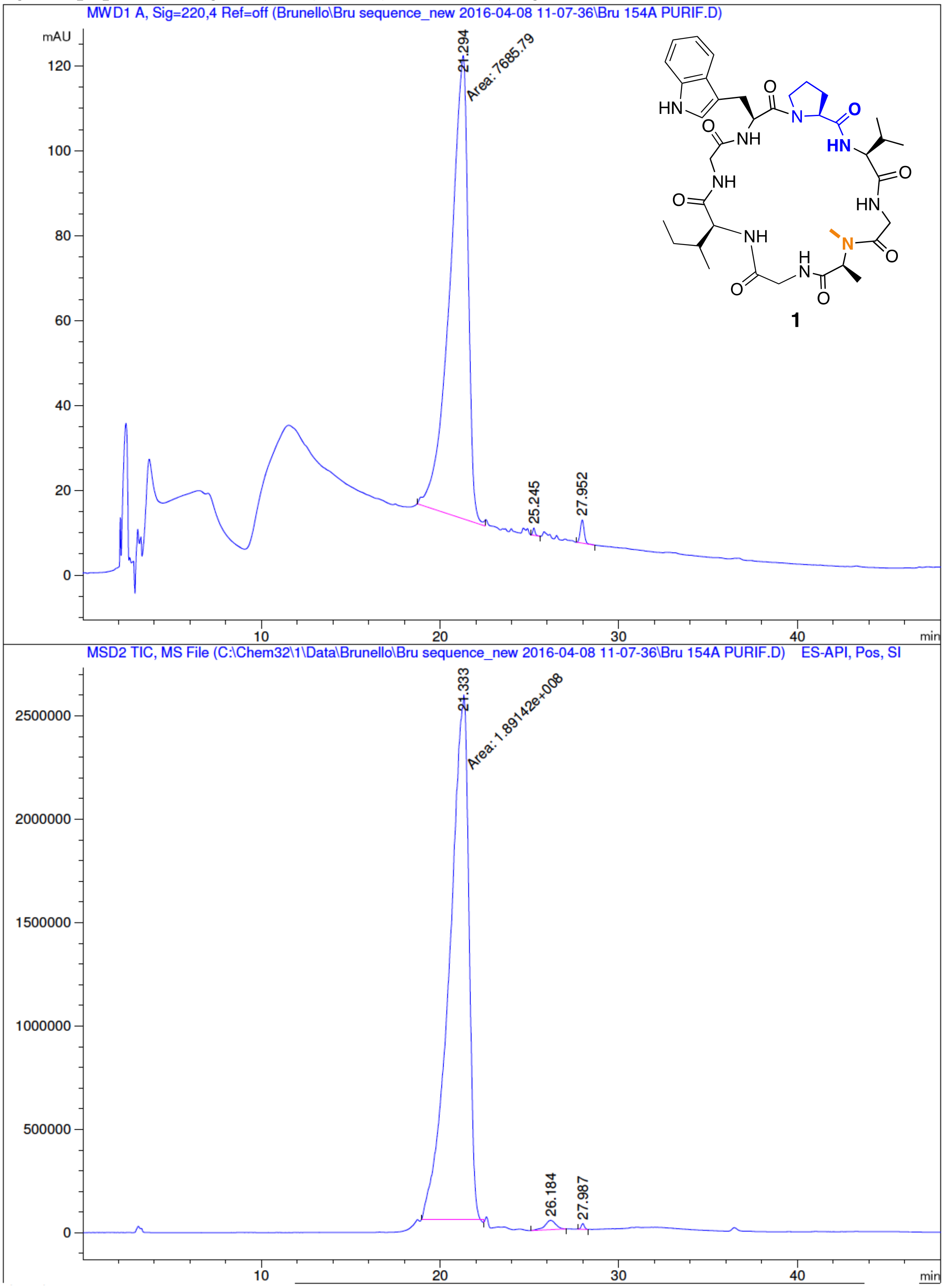

Cyclic peptide **2**; Cyclo(VGAG-**3-Abz**-GFP-), system A1

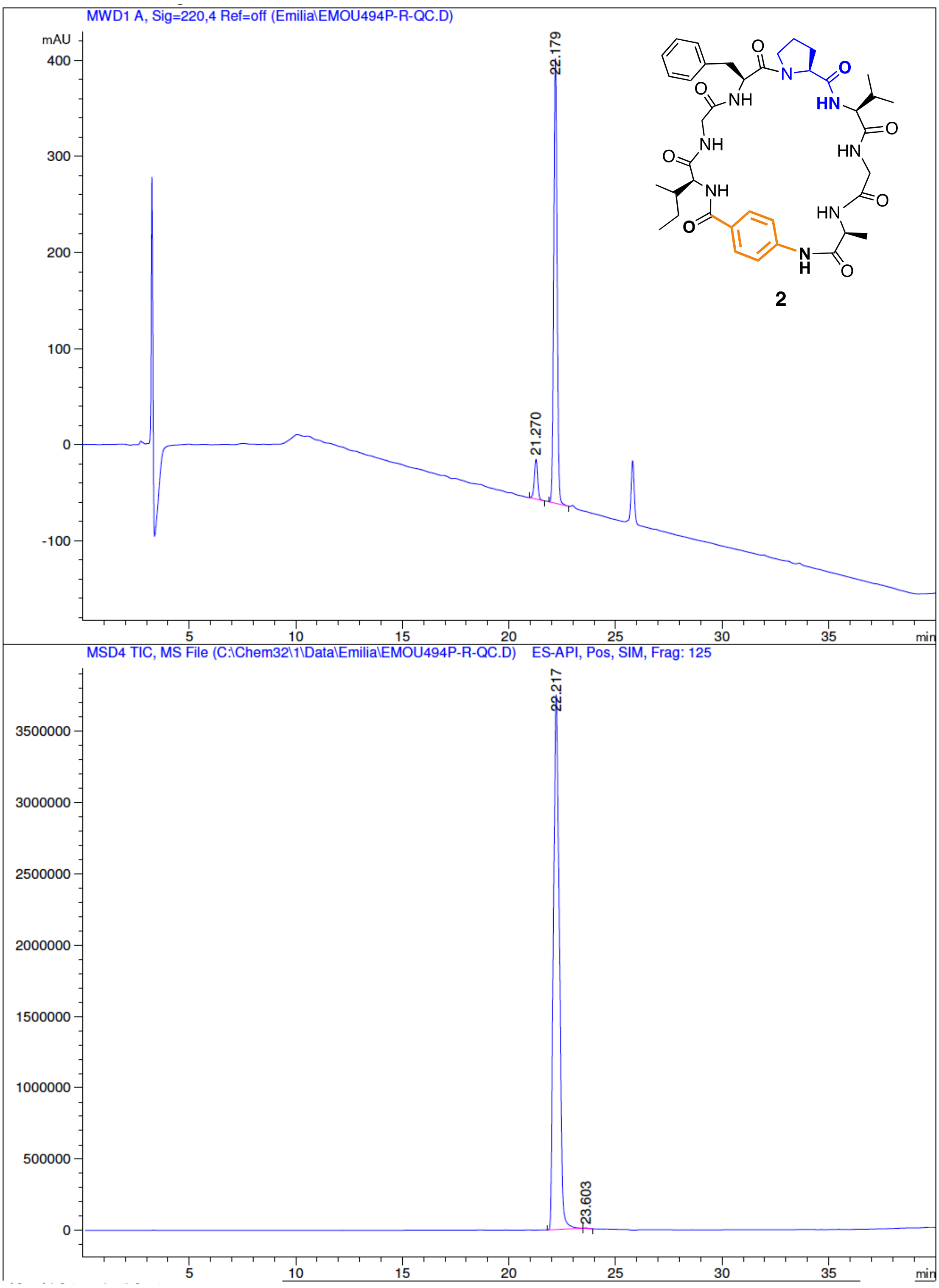

Cyclic peptide **3**; Cyclo(VGA-**4-Abz**-IGFP-), system A1

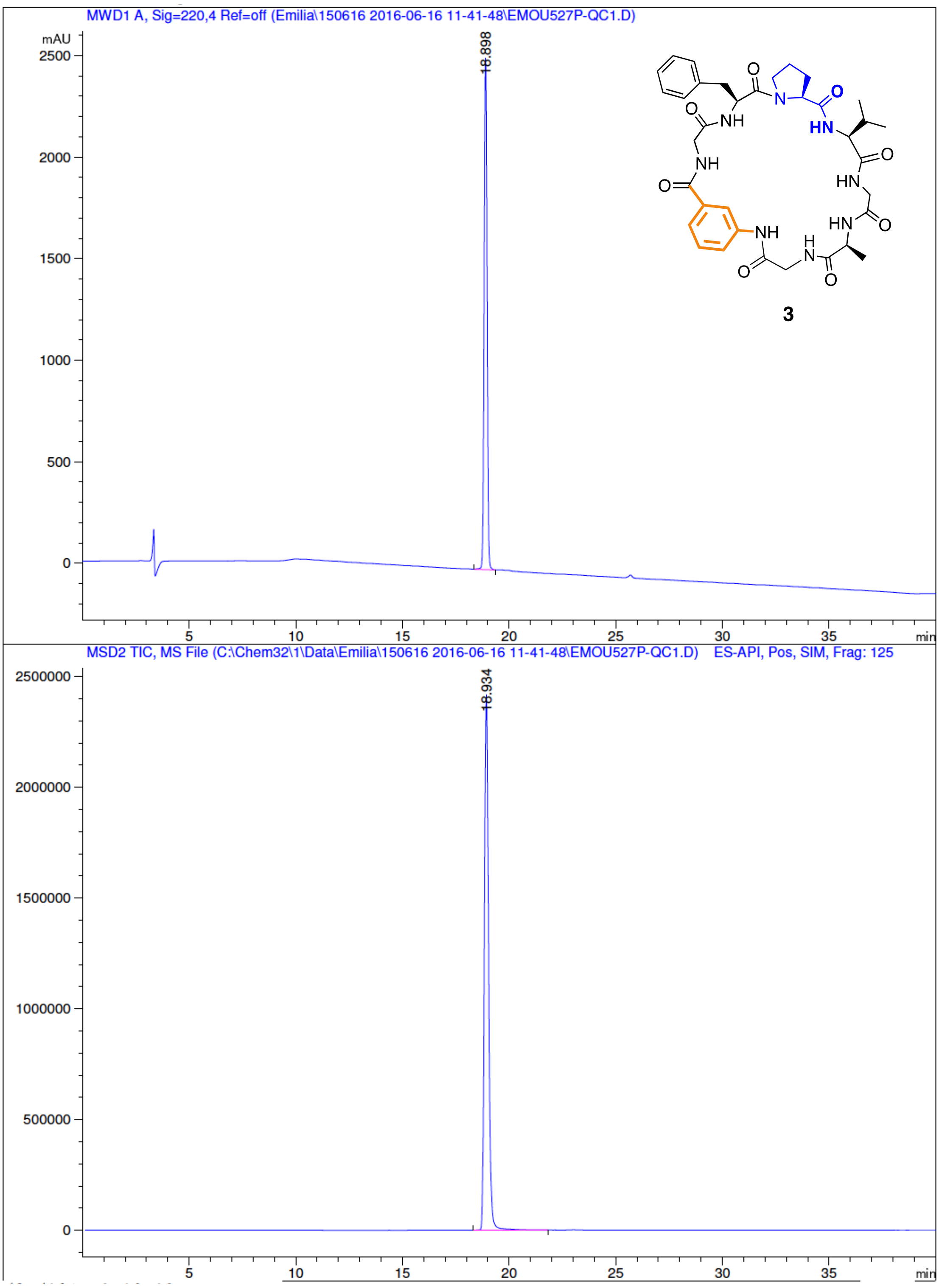

Cyclic peptide **4**; Cyclo(VGA-**Rib** -IGFP-), system A1

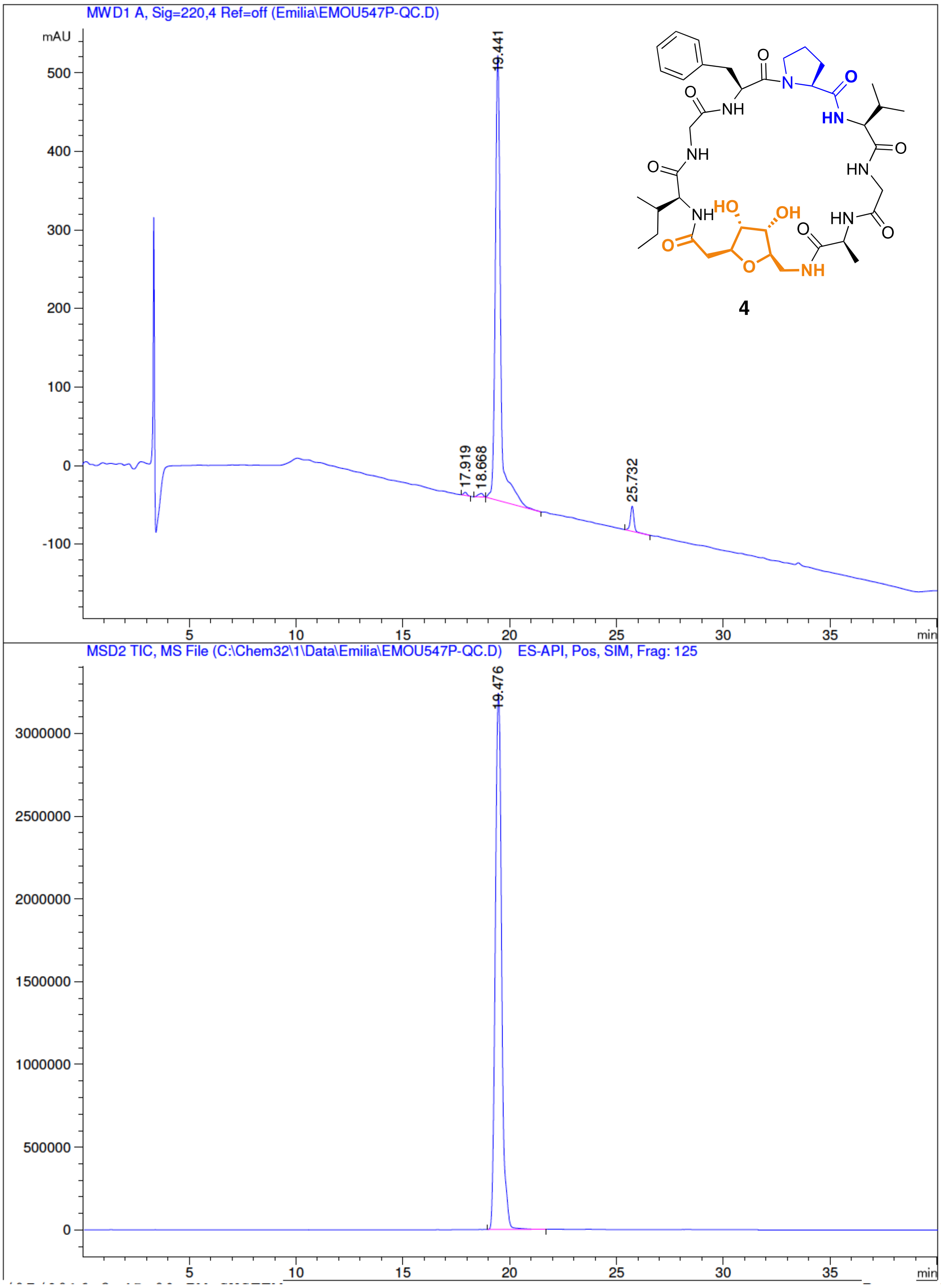

Cyclic peptide **5**; Cyclo(V-**8Aoc-8Aoc**-FP-), system A1

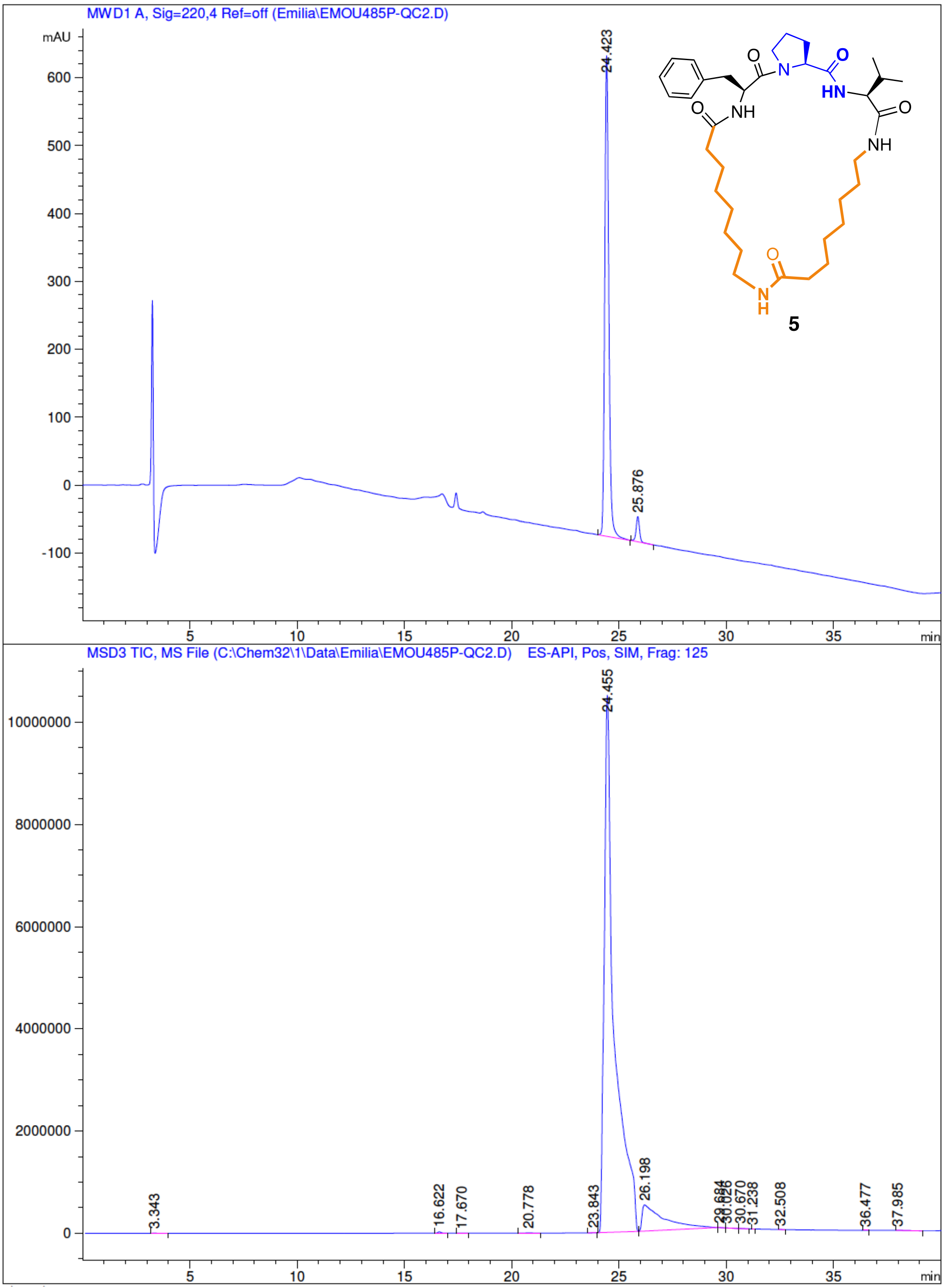

Cyclic peptide **6**; Cyclo(V-(PEG)$_4$-FP-), system A1

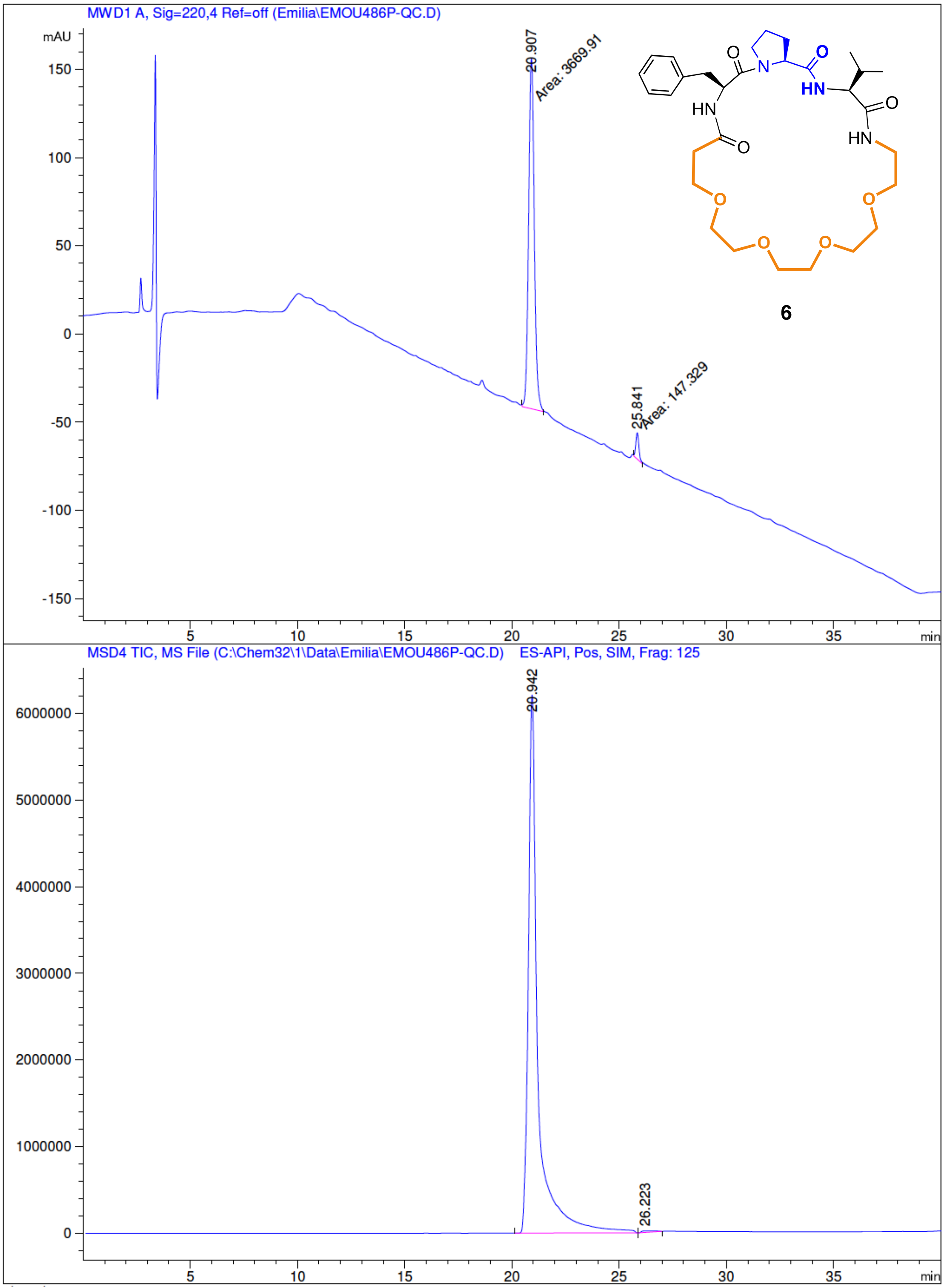

Cyclic peptide **7a**; Cyclo(V-**(PEG)$_4$**-FThz-), system B

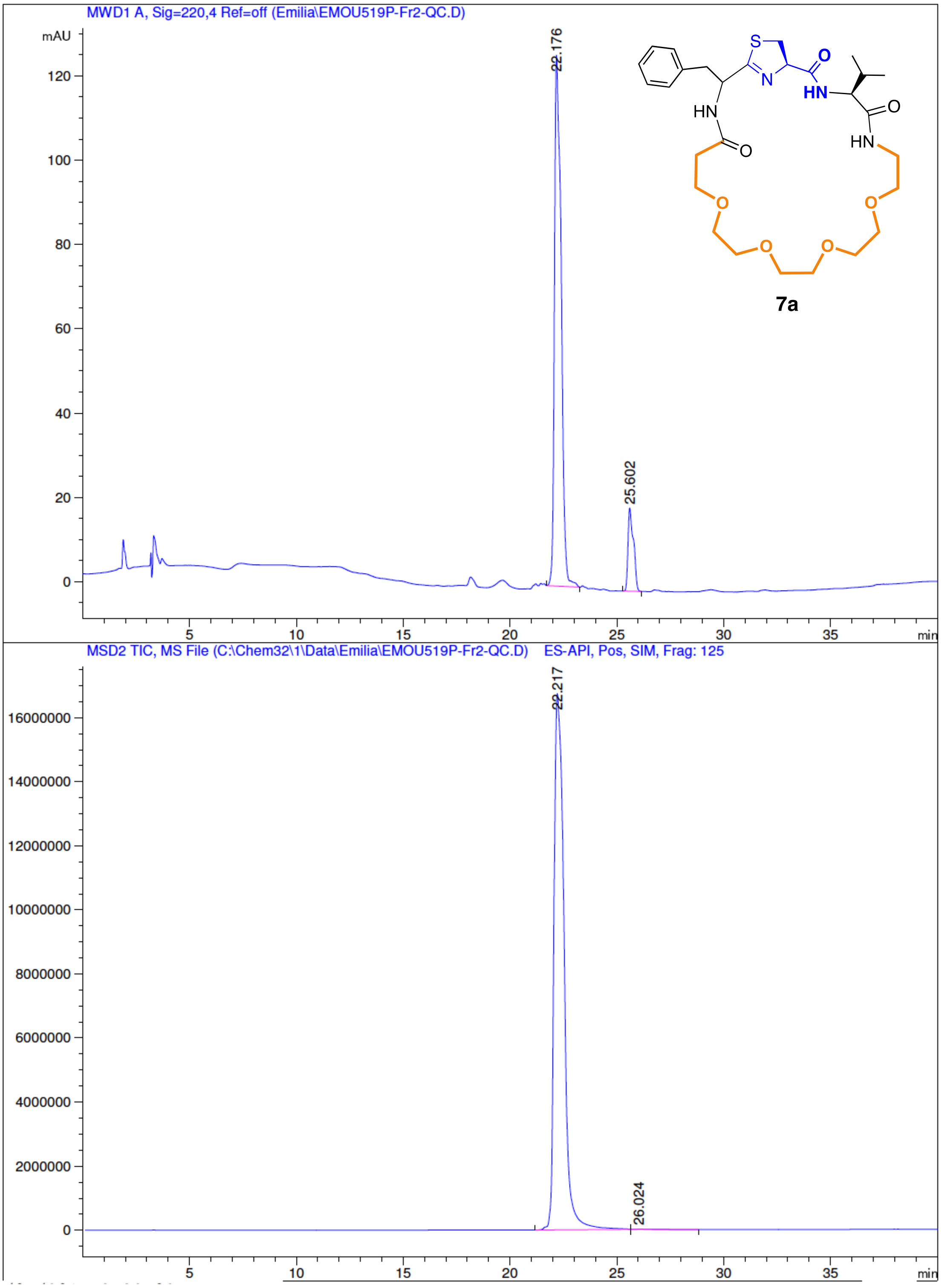

Cyclic peptide **7b**; Cyclo(V-**(PEG)$_4$**-FThz-), system B

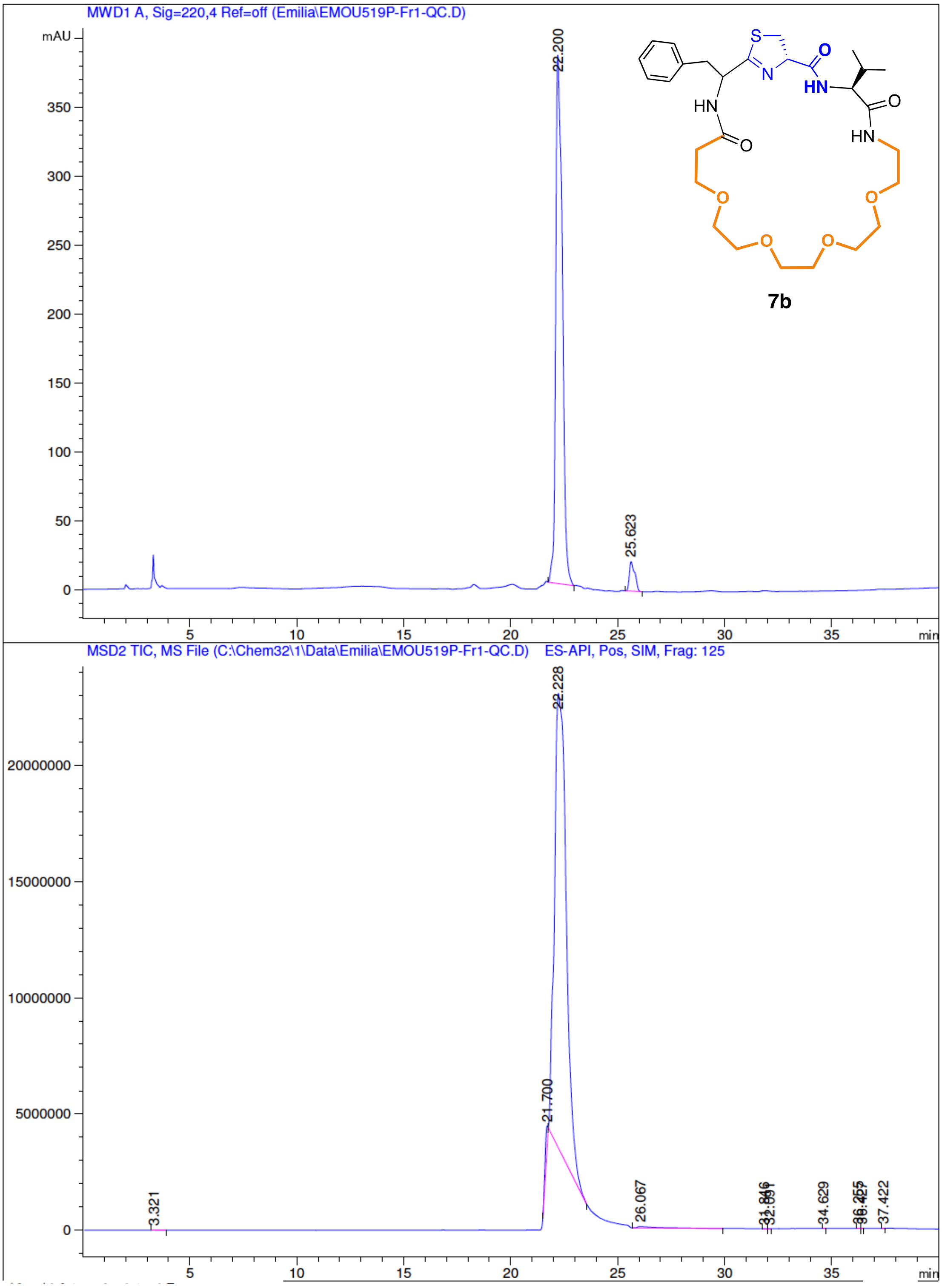

Cyclic peptide **7c**; Cyclo(V-**(PEG)$_4$**-FThl-), system A

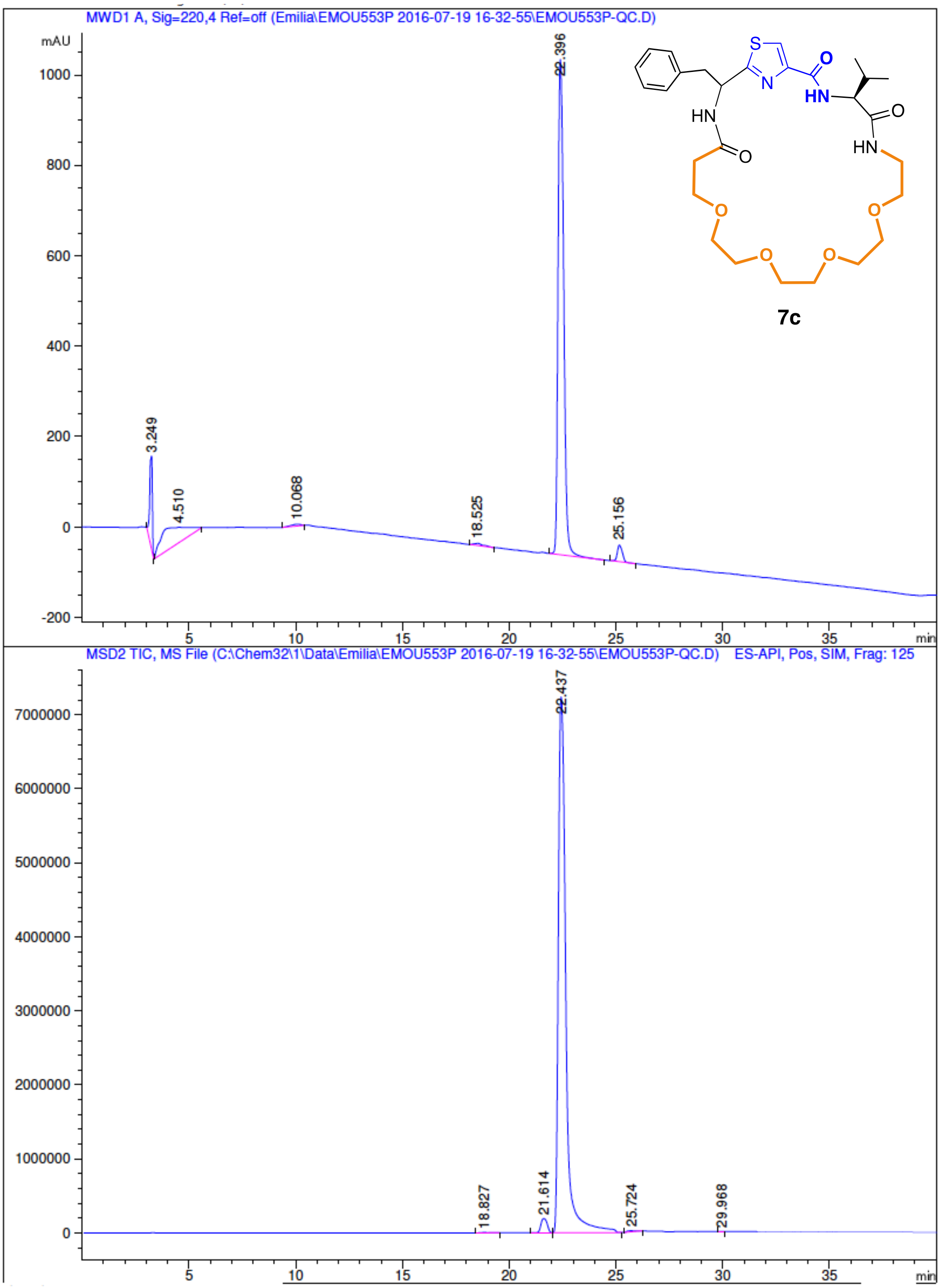

XIV. LC traces and MS spectra of the crude resulting peptides after cleavage from the resin of peptides 8-11

The resulting cleaved peptides **8-11** were analyzed by LCMS before the PatGmac macrocyclization. The UV trace is recorded at 280 nm. The HPLC methods used are described in section II.

Peptide **8** analogue; VGA-β-Ala-IGWPAYD-Doc, system C

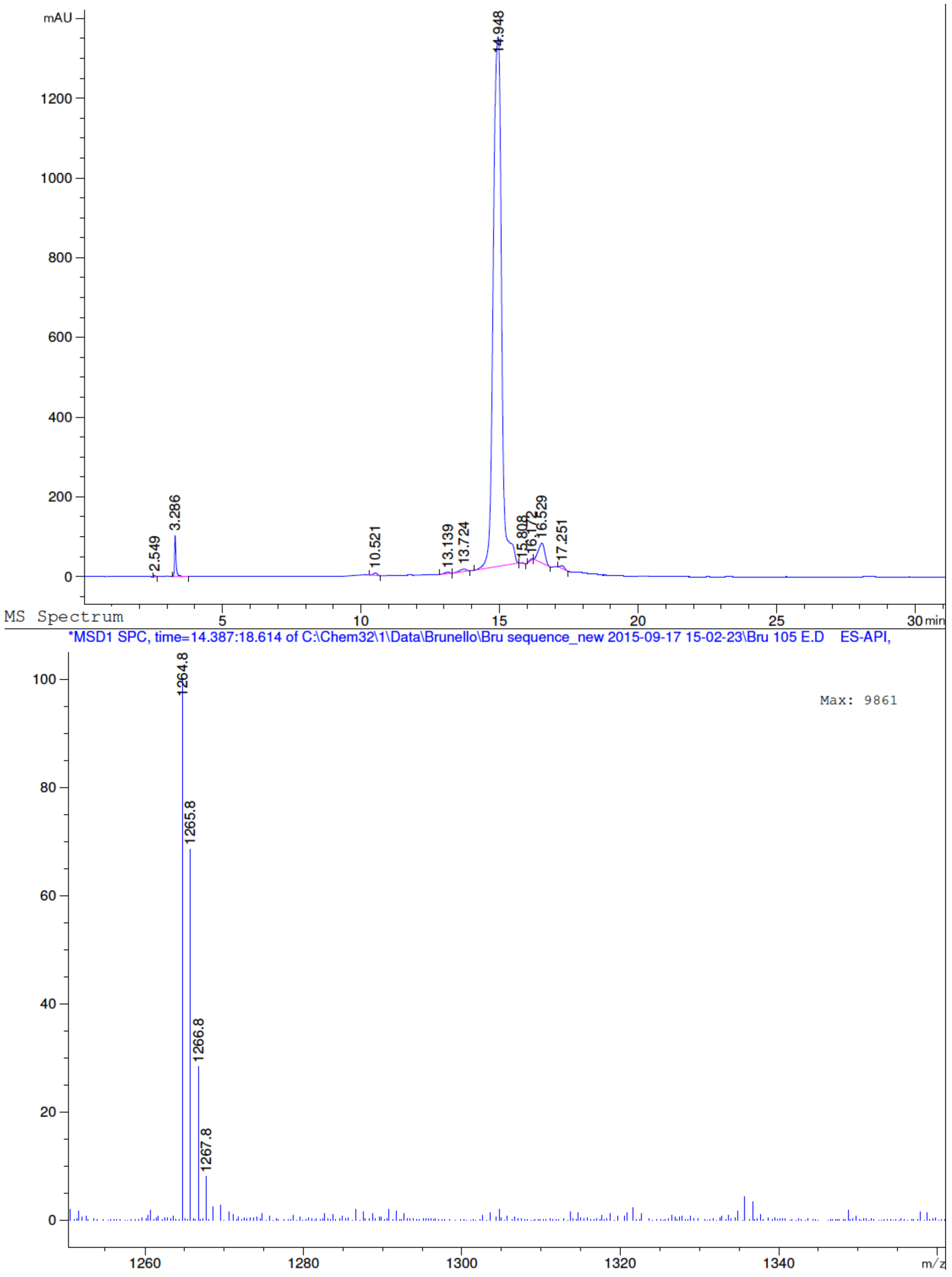

Peptide **9** analogue; VGA-GABA-IGWPAYD-Doc, system C

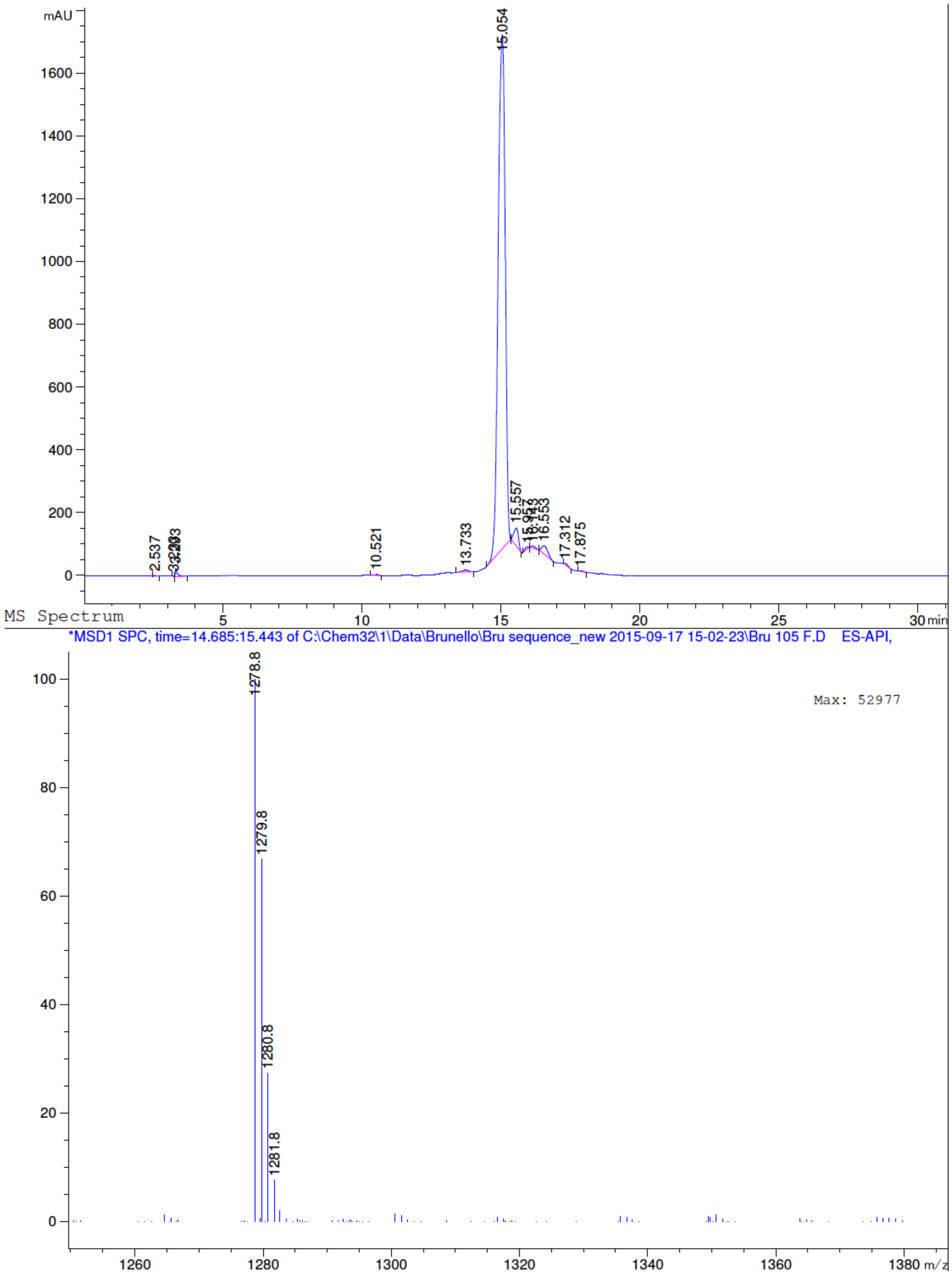

Peptide **10** analogue; VGA-Doc-IGWPAYD-Doc, system C

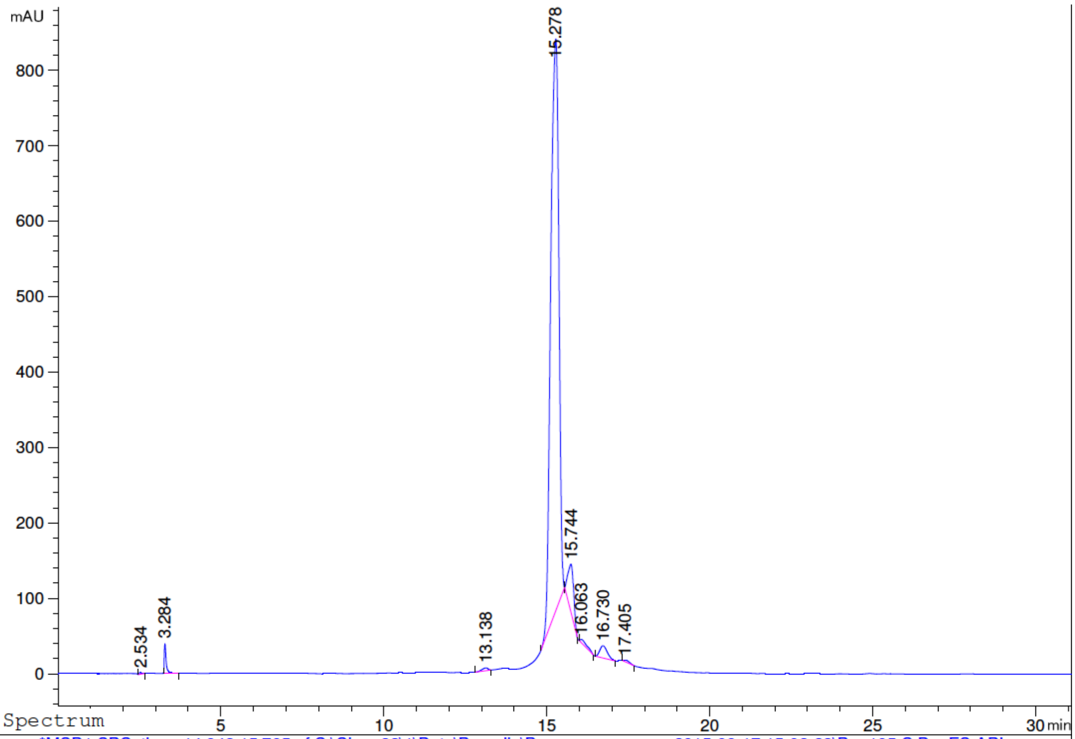

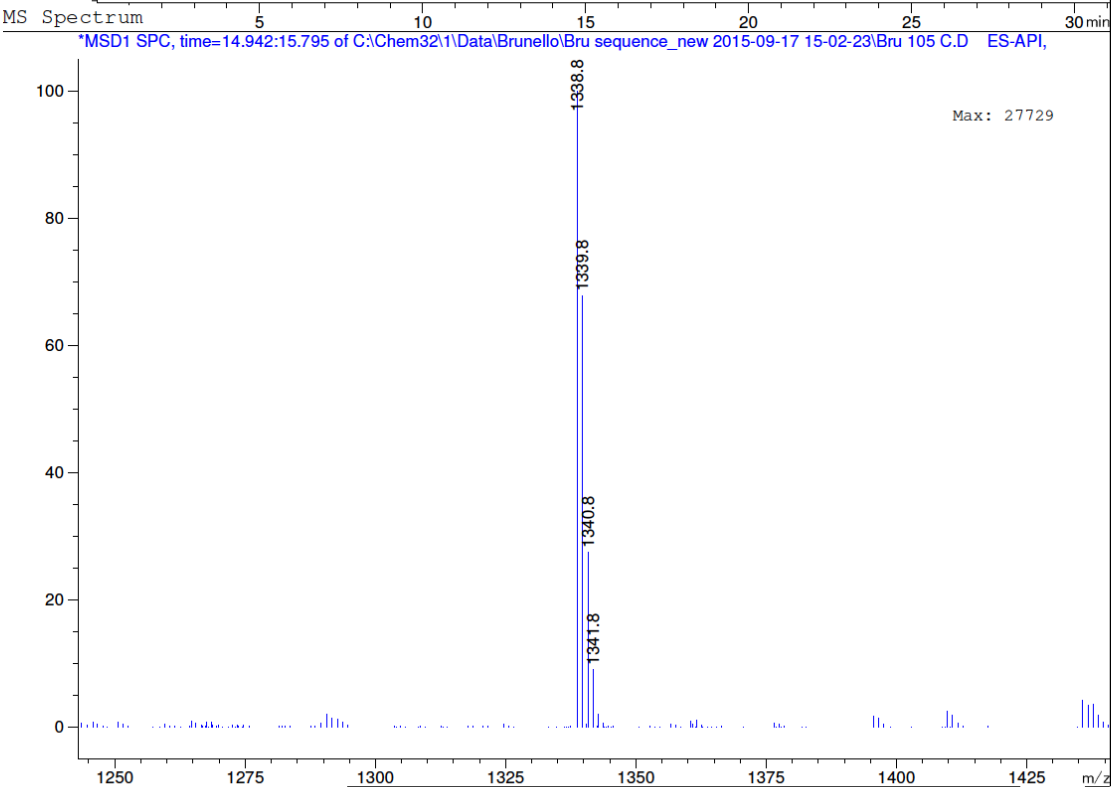

Peptide **11** analogue; VG(***N*-Me**)AGIGWPAYD-Doc, system C

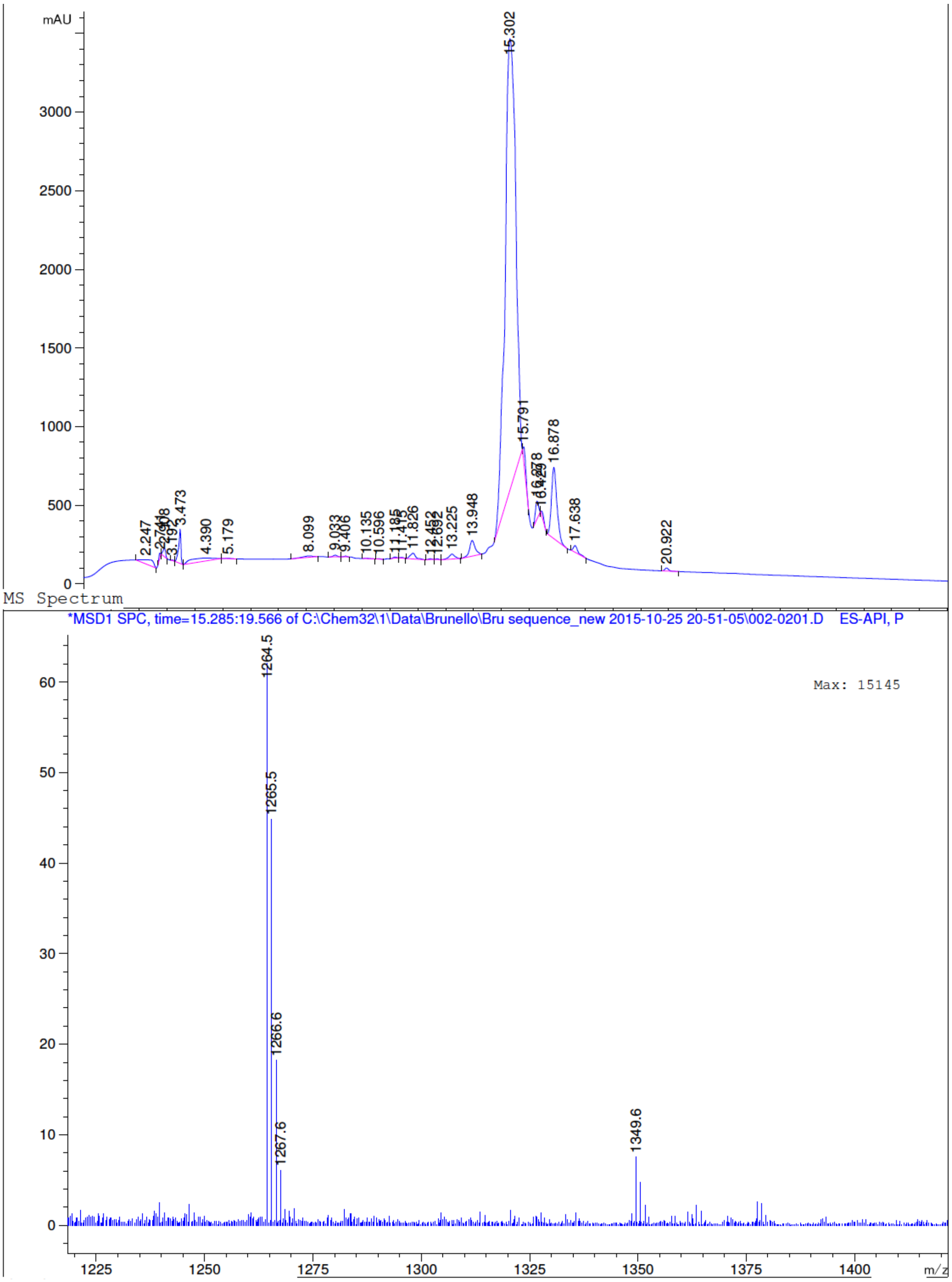

XV. LC-MS traces of starting hybrid peptides 12-26, 36-38

For each of the precursor hybrid peptides **12-26,** the UV trace at 220 nm obtained by HPLC is complemented by its corresponding LCMS trace at the desired molecular weight (Single Ion Monitoring SIM mode). The HPLC methods used are described in section II.

Peptide **12**; VGA-**2-Abz**-IGFPAYD-NH_2, system A1

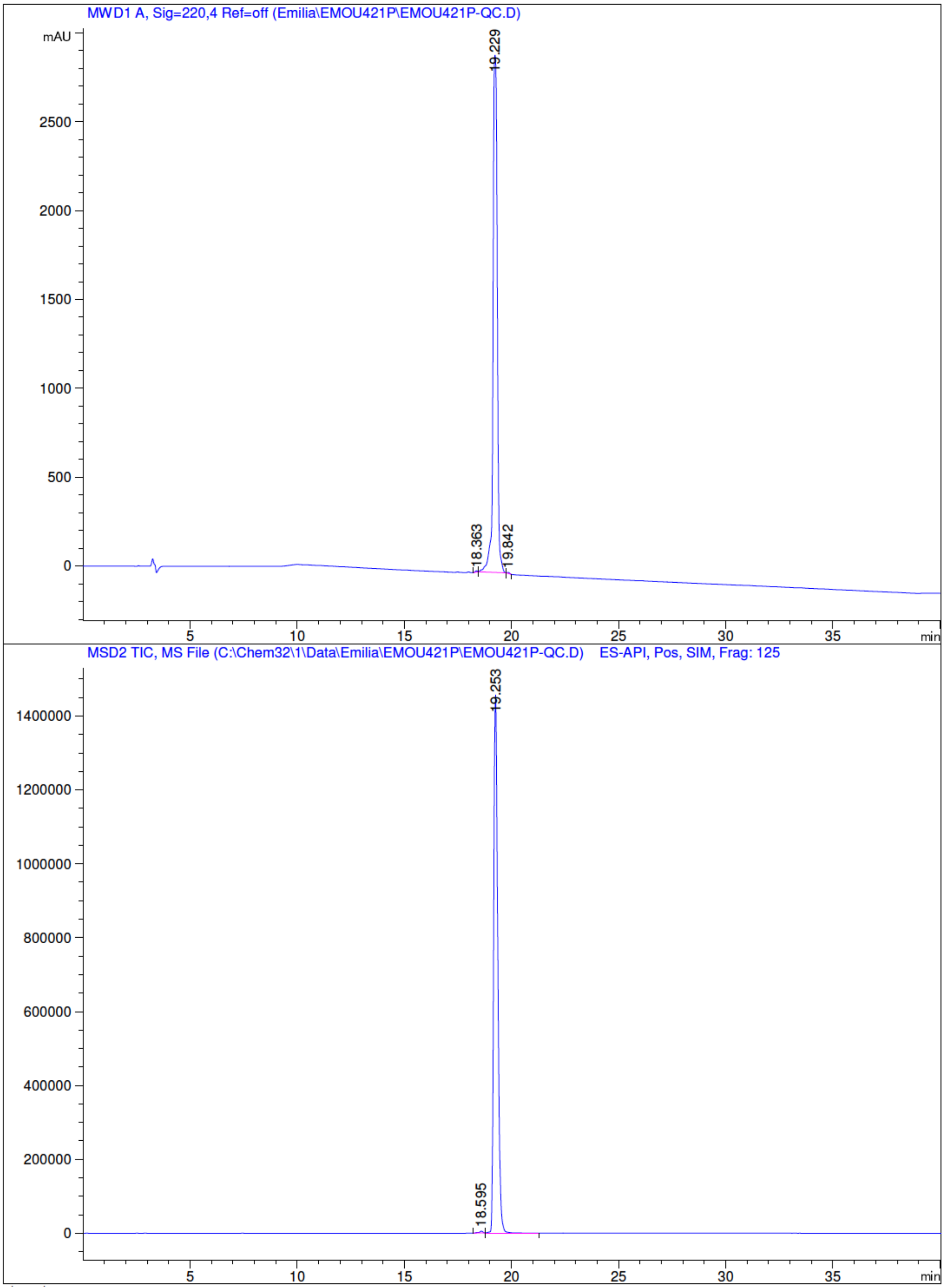

Peptide **13**; VGA-**3-Abz**-IGFPAYD-NH_2, system A1

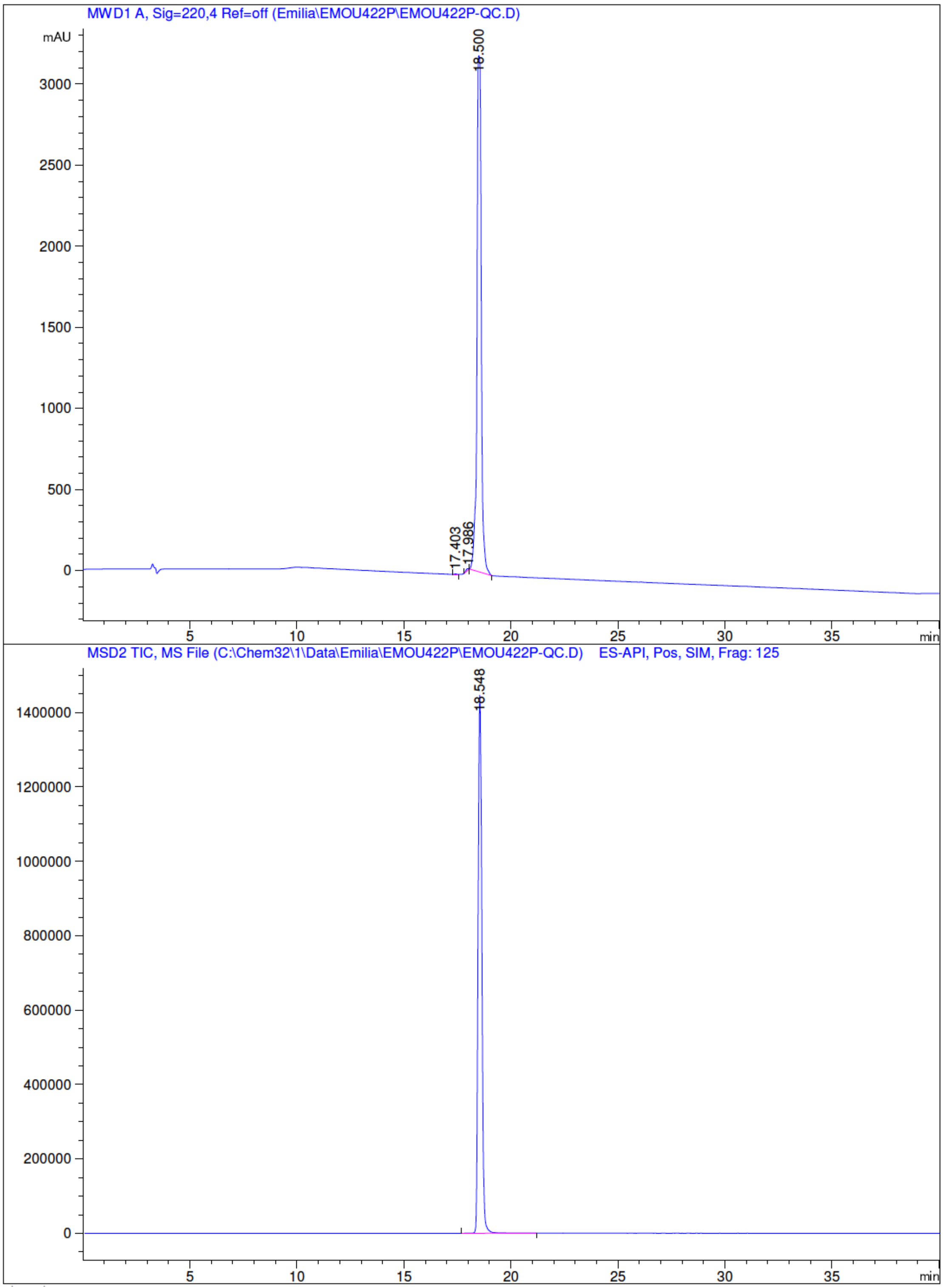

Peptide **14**; VGAG-**4-Abz**-IGFPAYD-NH_2, system A1

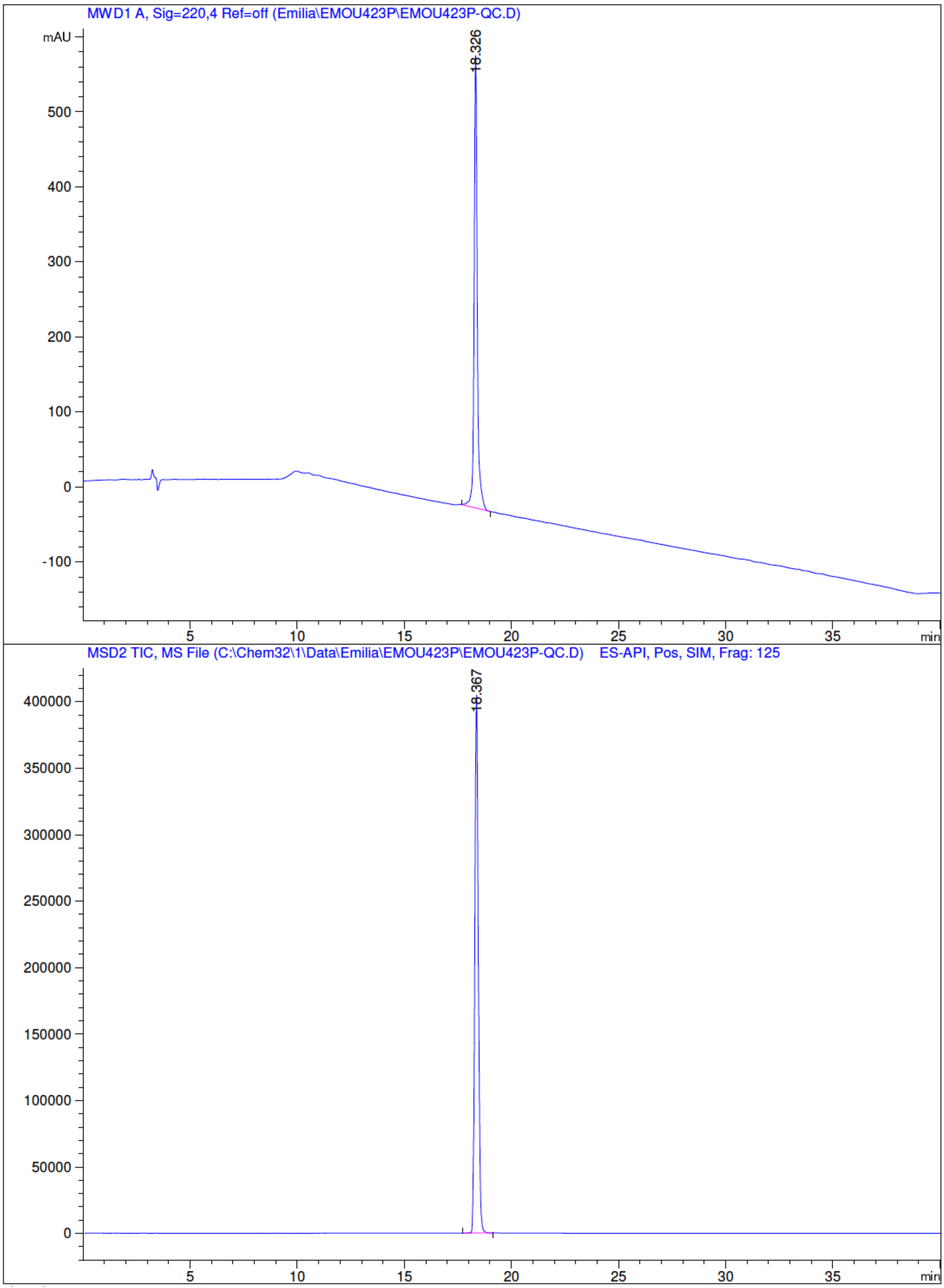

Peptide **15**; VGAG-**2-Abz**-GFPAYD-NH_2, system A1

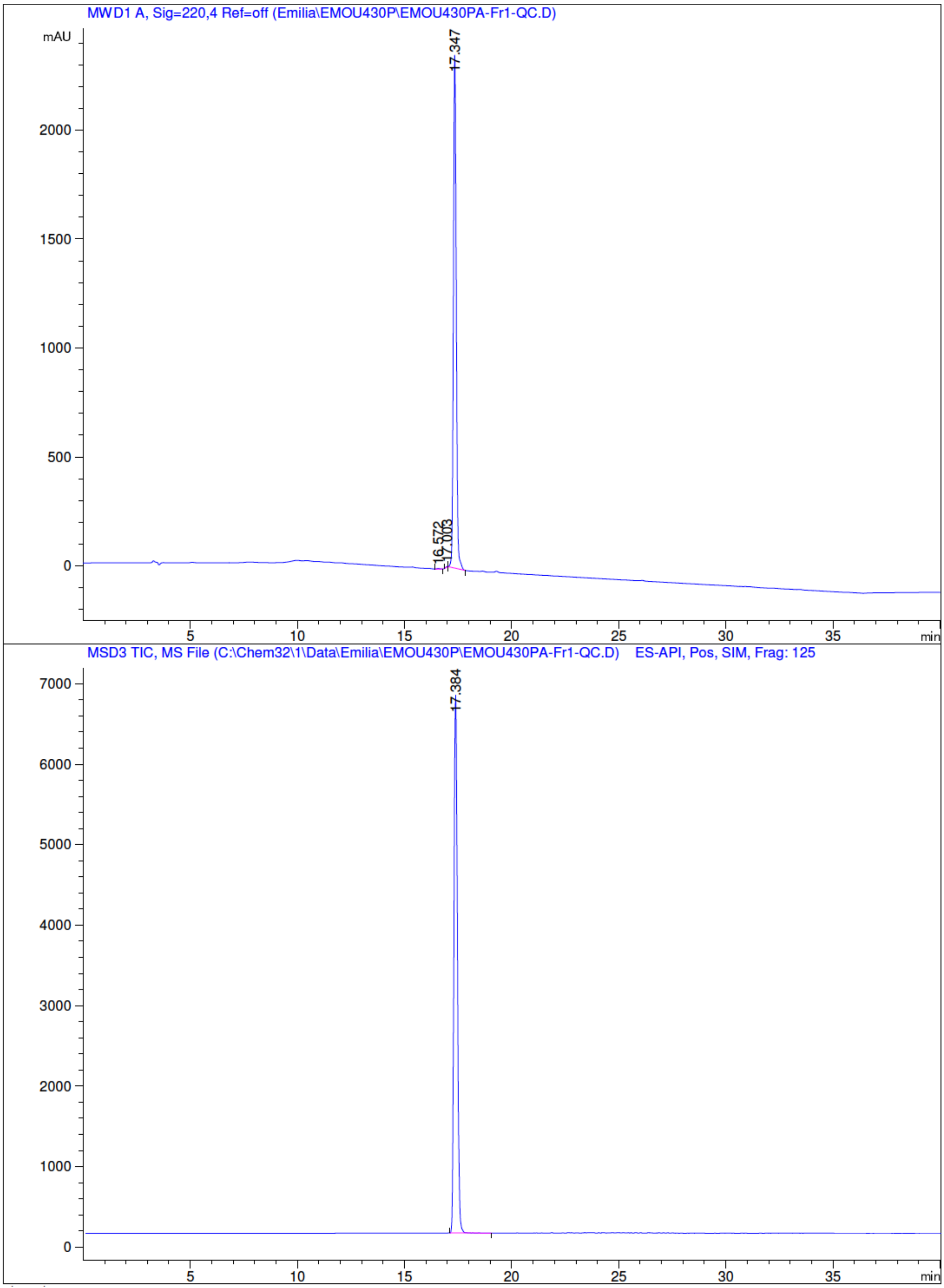

Peptide **16**; VGAG-**3-Abz**-GFPAYD-NH_2, system A1

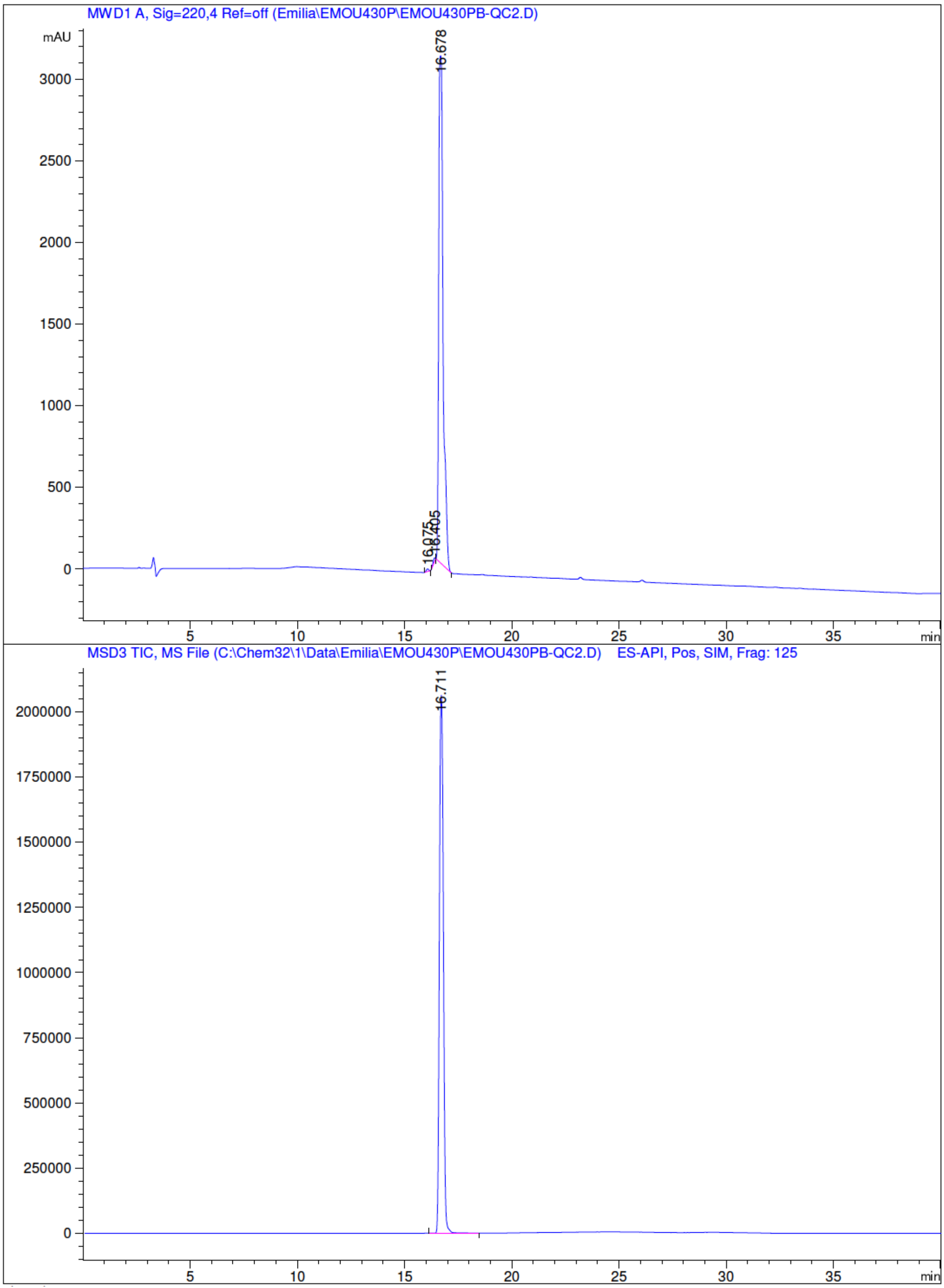

Peptide **17**; VGAG-**4-Abz**-GFPAYD-NH_2, system A1

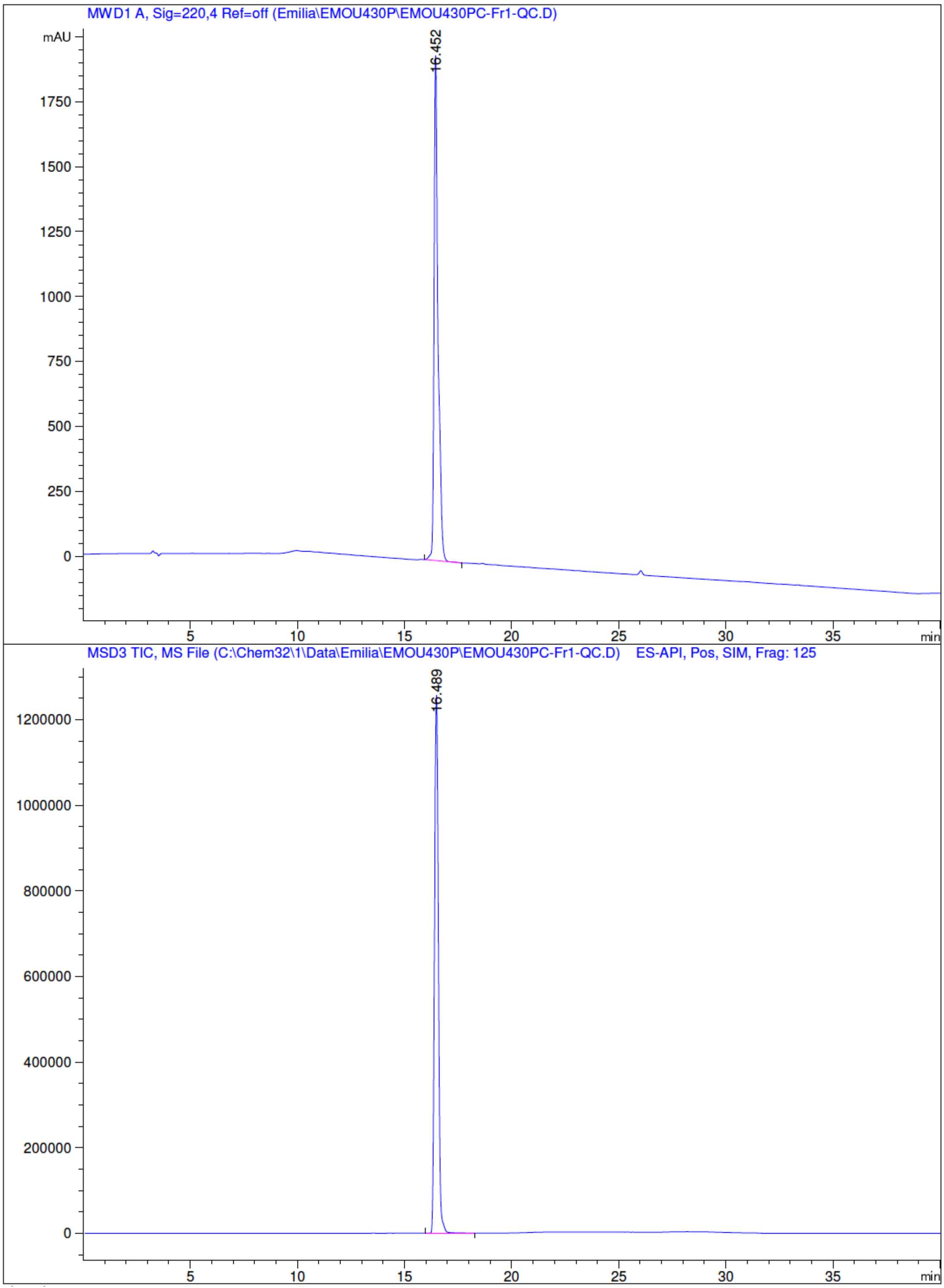

Peptide **18**; VGAG-**Rib**-IGFPAYD-NH_2, system A1

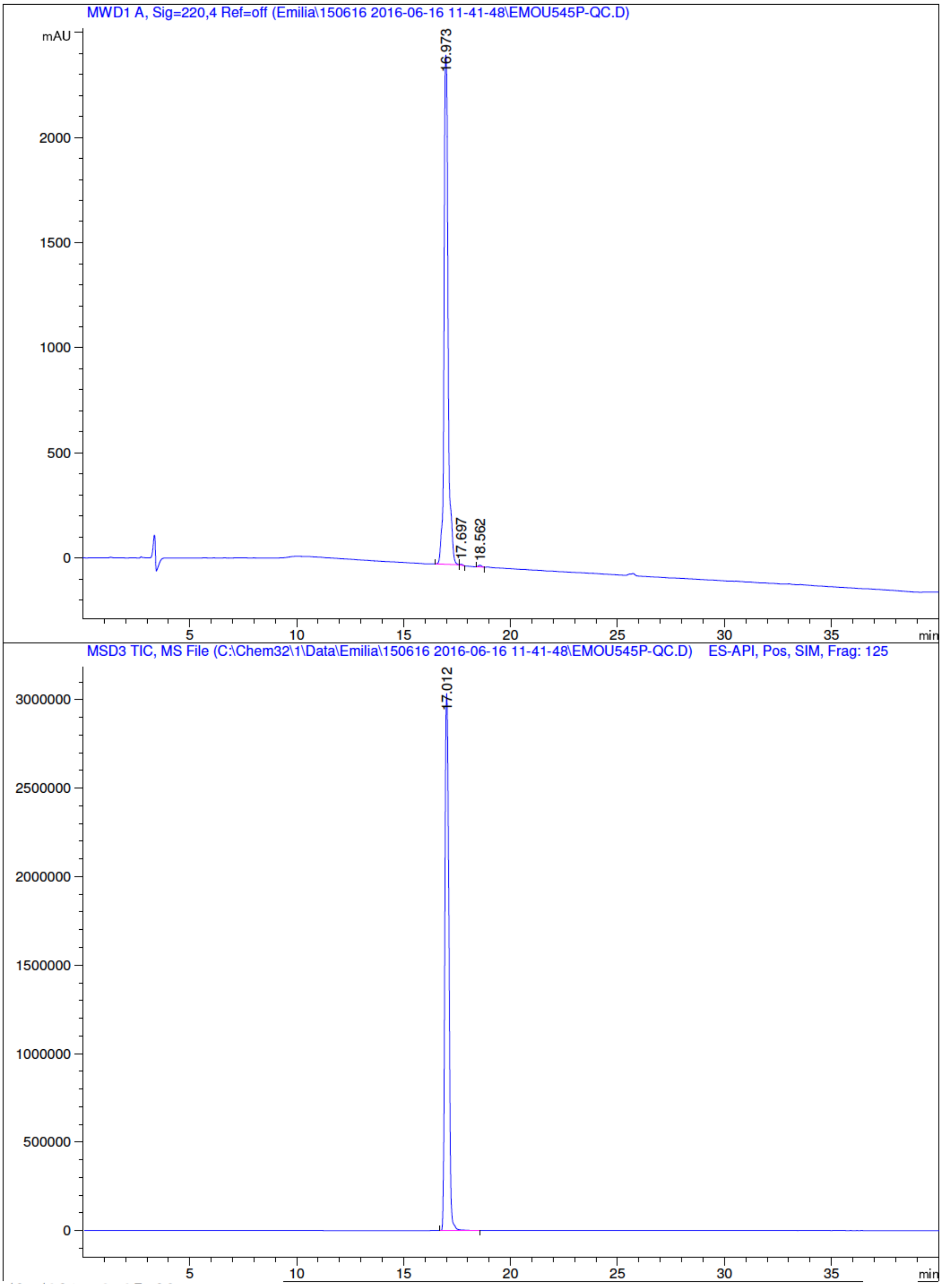

Peptide **19**; H-**8Aoc**-AGIGFPAYD-NH_2, system A1

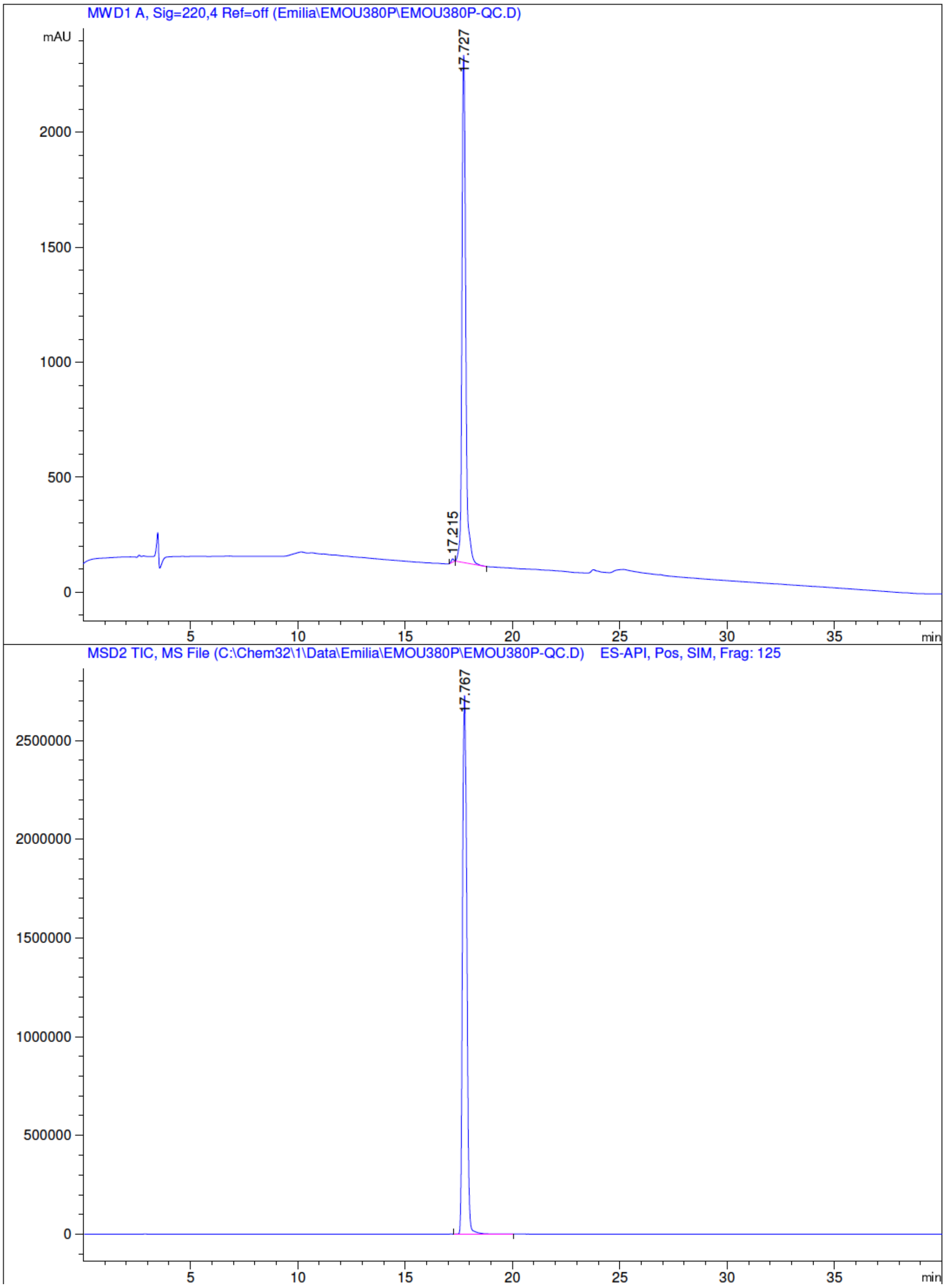

Peptide **20**; V-**7Ahp**-GAGFPAYD-NH_2, system A1

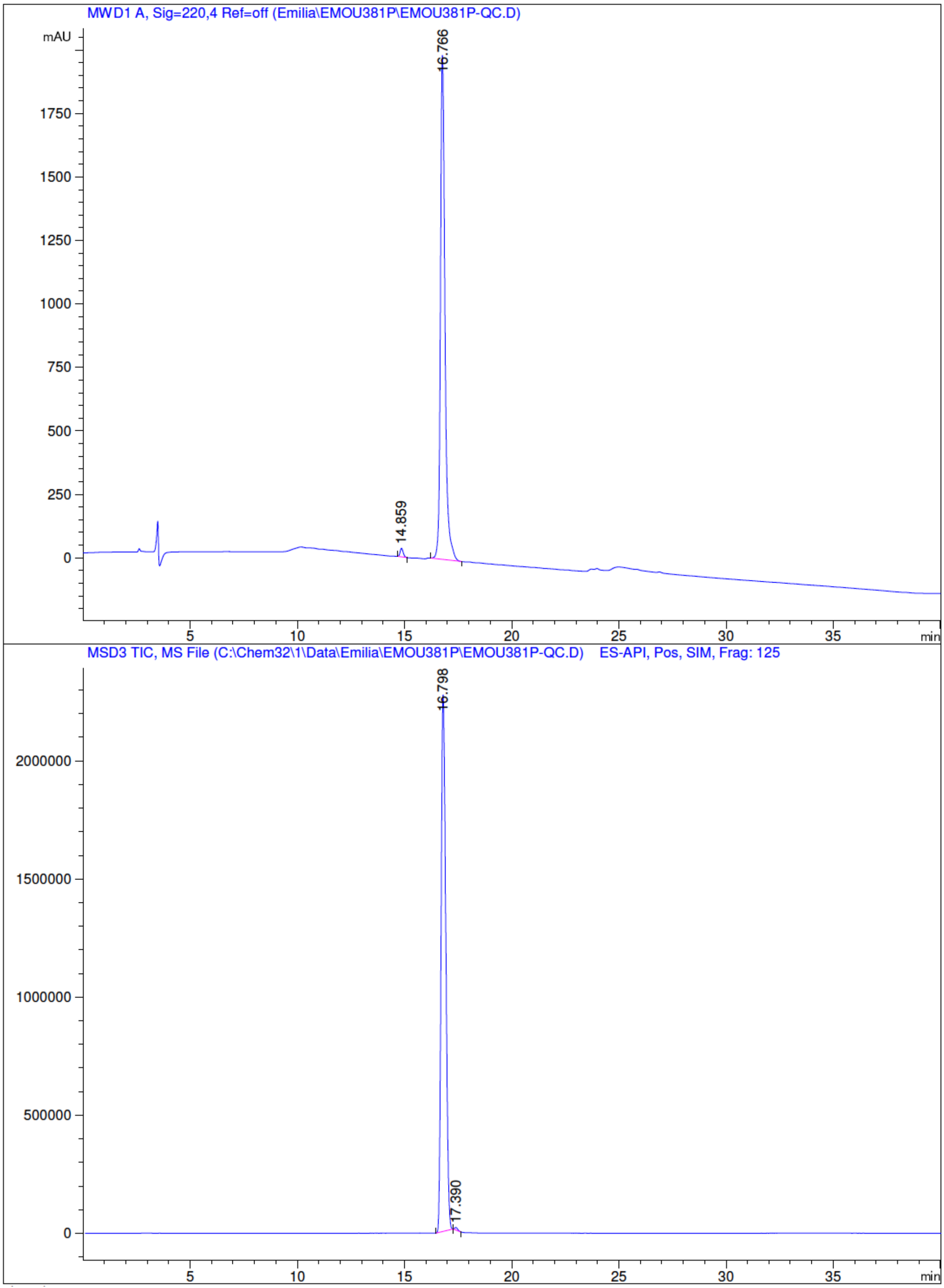

Peptide **21**; VGAG-**7Ahp**-PAYD-NH_2, system A1

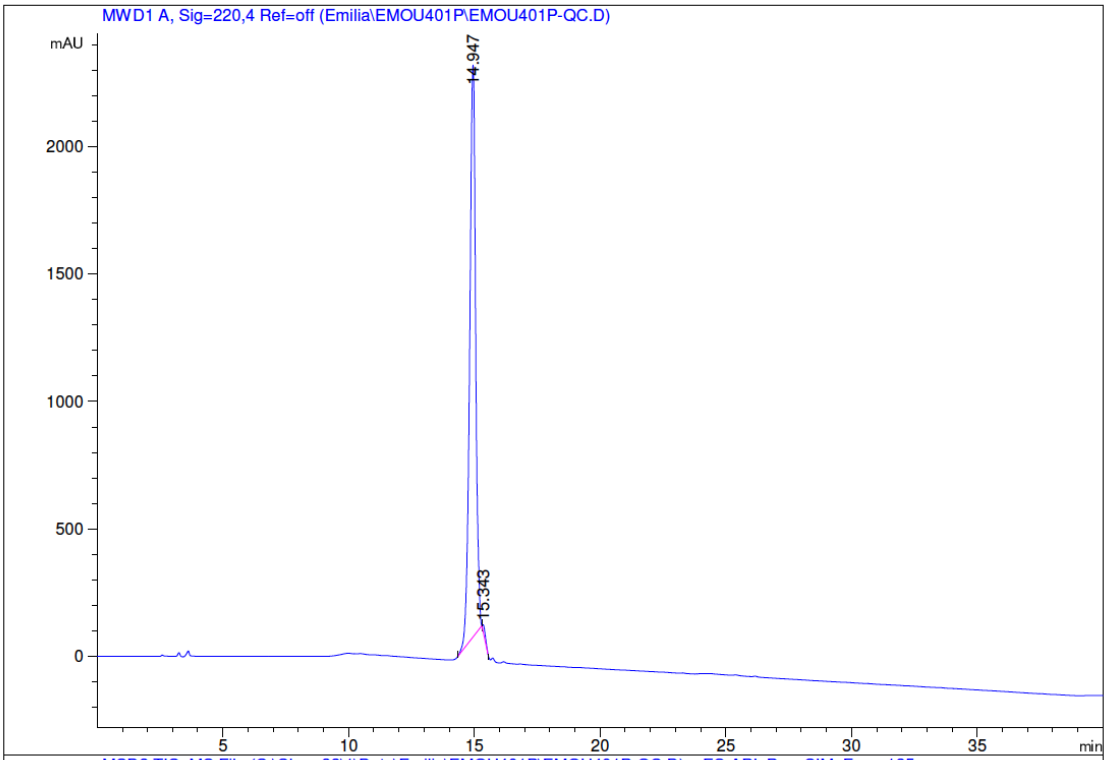

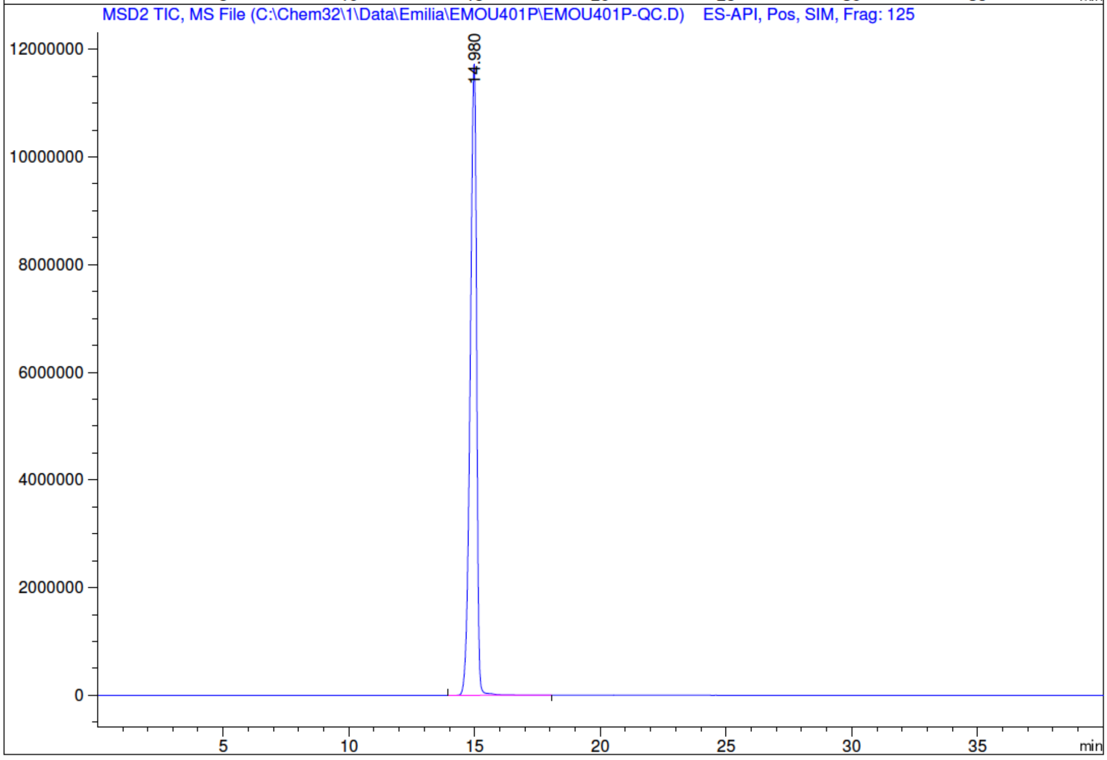

Peptide **22**; VGAG-**8Aoc**-FPAYD-NH_2, system A1

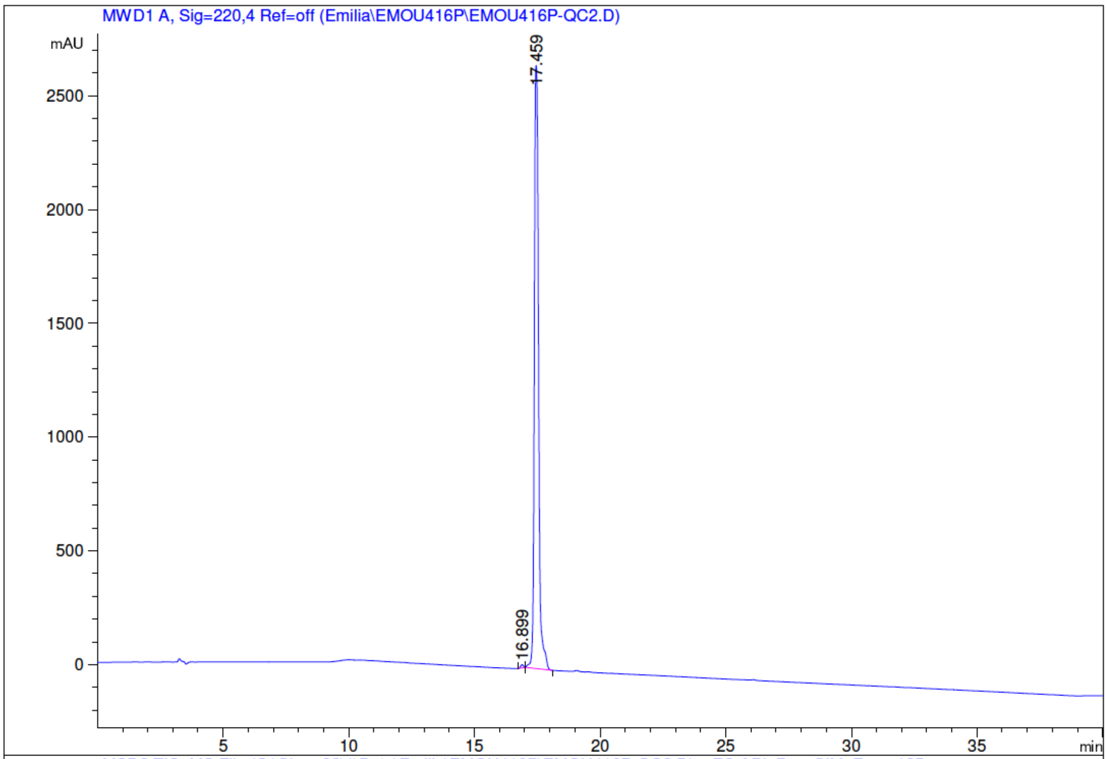

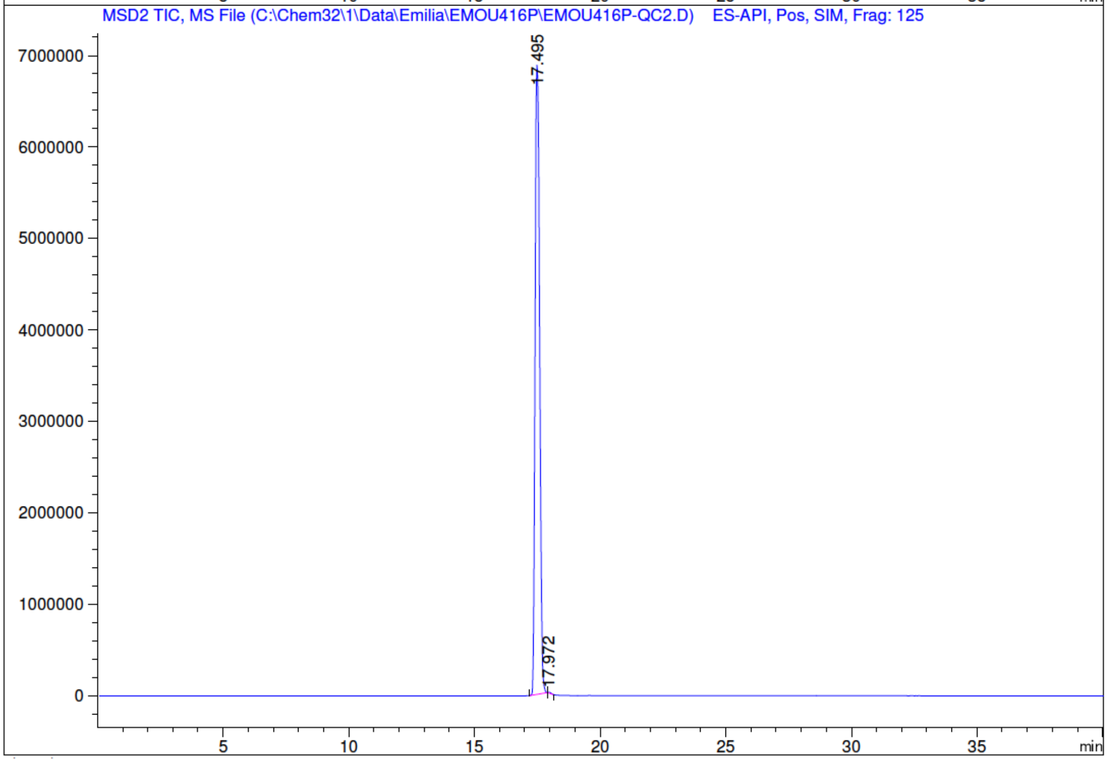

Peptide **23**; V-**8Aoc**-FPAYD-NH_2, system A1

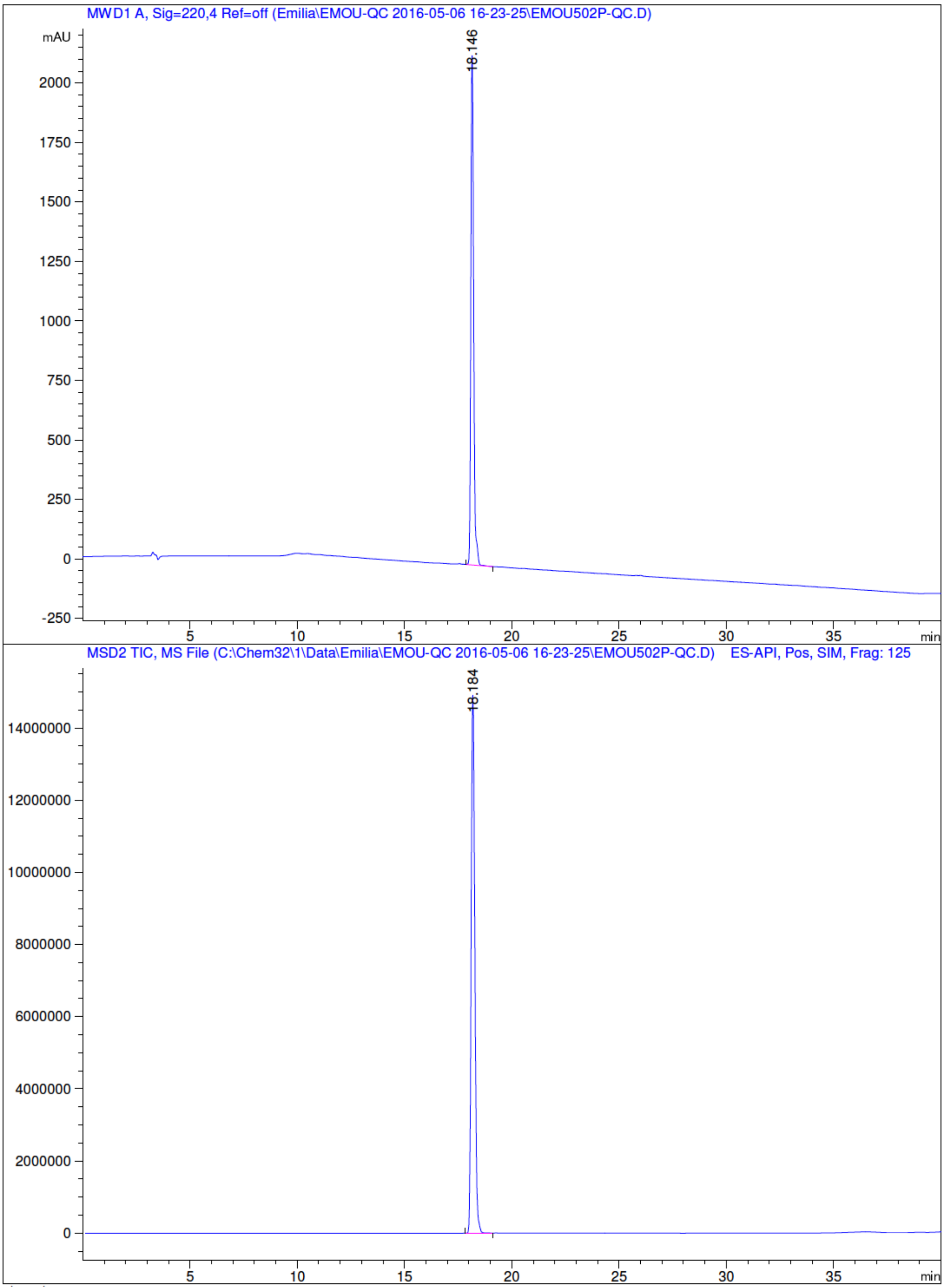

Peptide **24**; V-**8Aoc-8Aoc**-FPAYD -NH_2, system A1

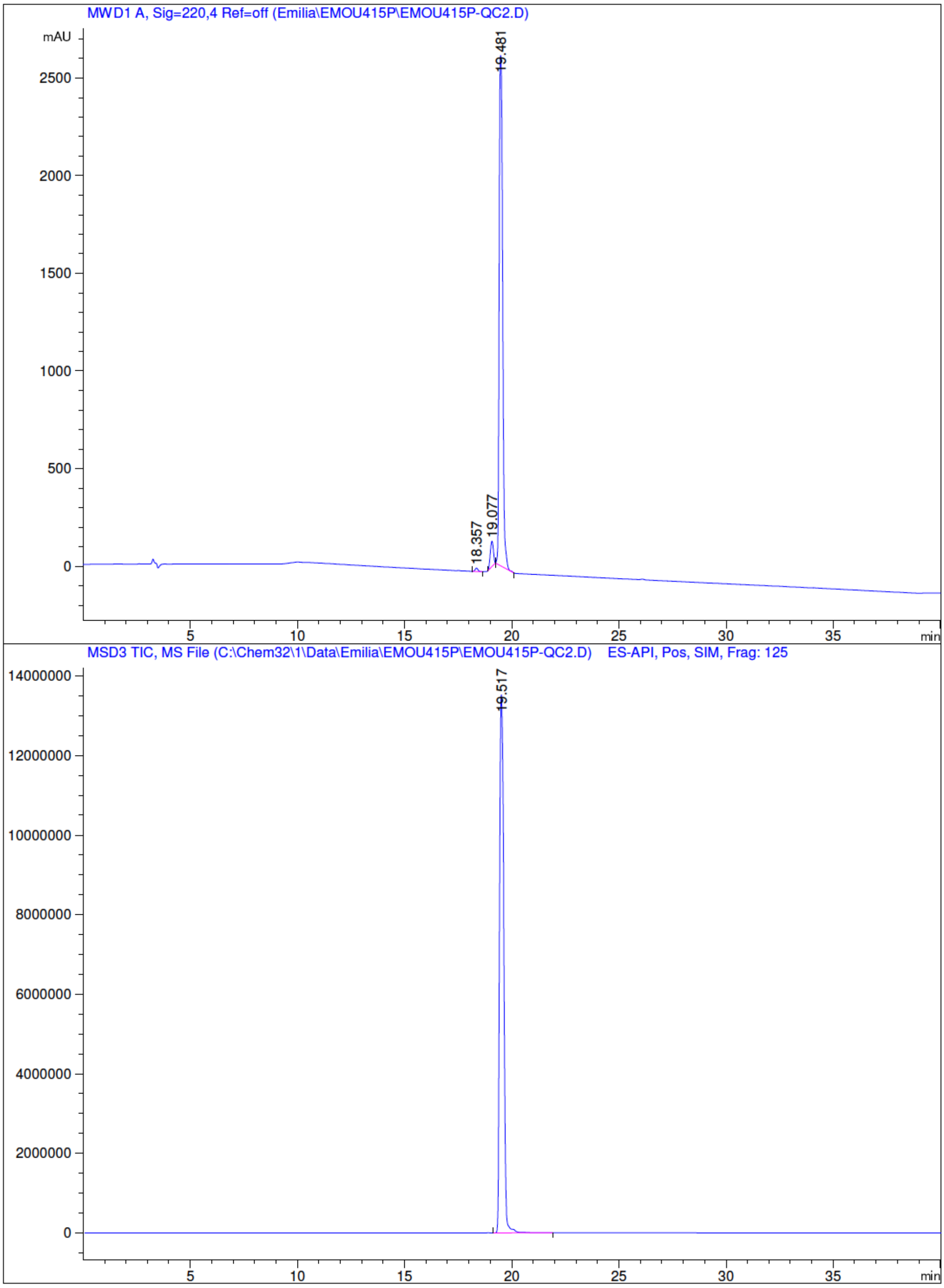

Peptide **25**; V-(**PEG)$_4$**-FPAYD -NH_2, system A1

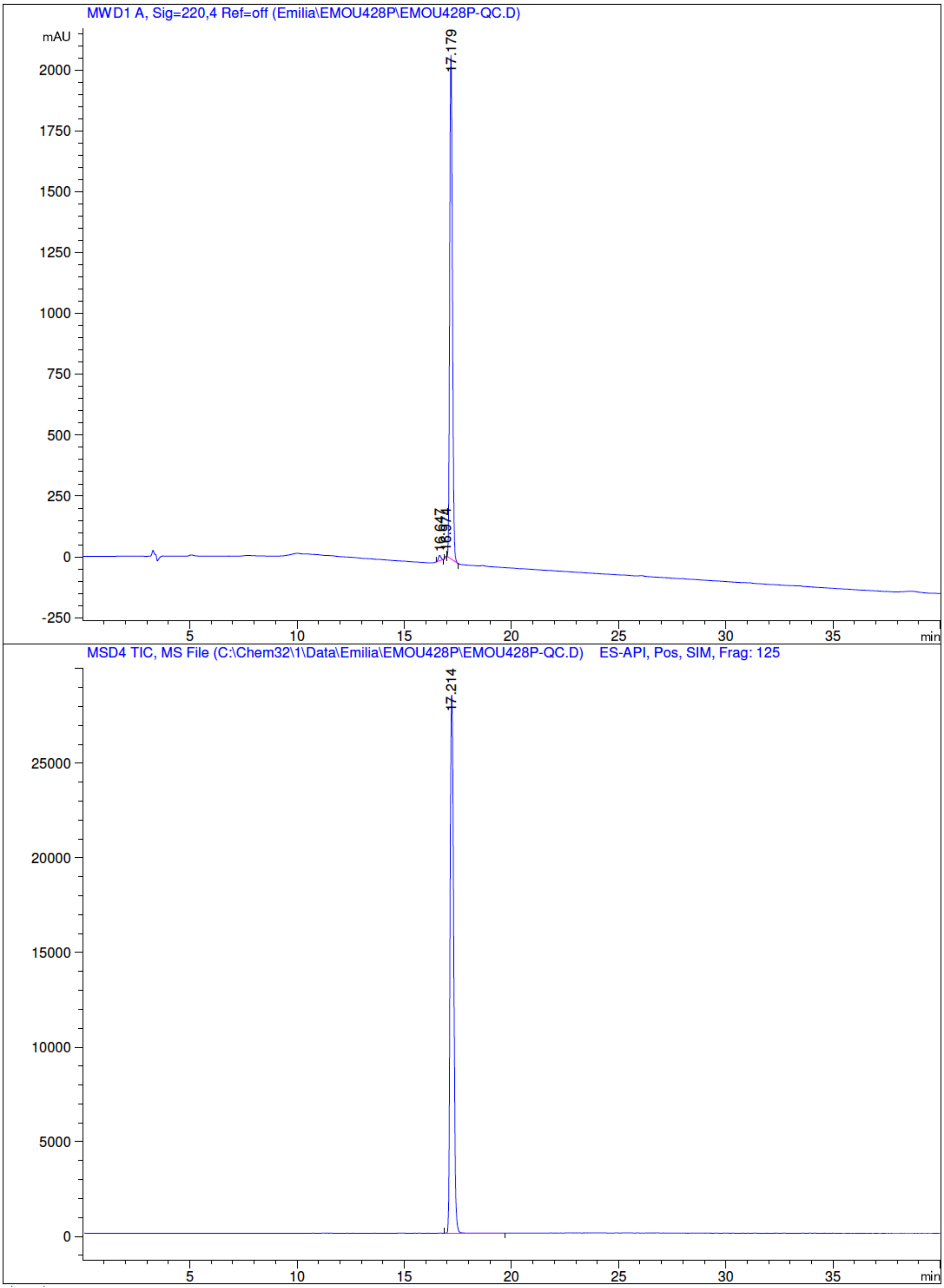

Peptide **26**; V-(**PEG)₄**-FCAYD -NH_2, system A1

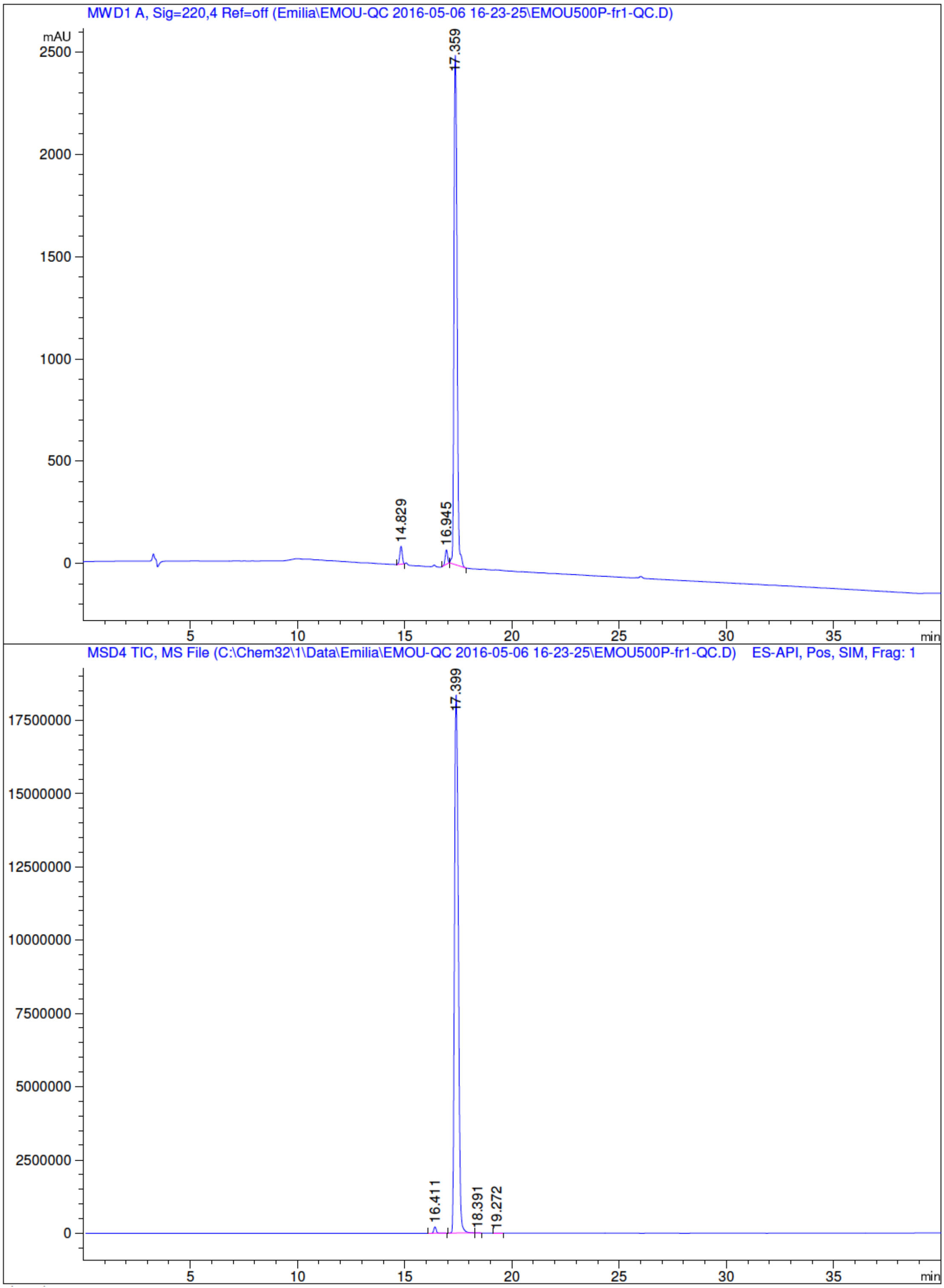

Peptide **36**; V-**7Ahp-7Ahp**-PAYD -NH_2, system A1

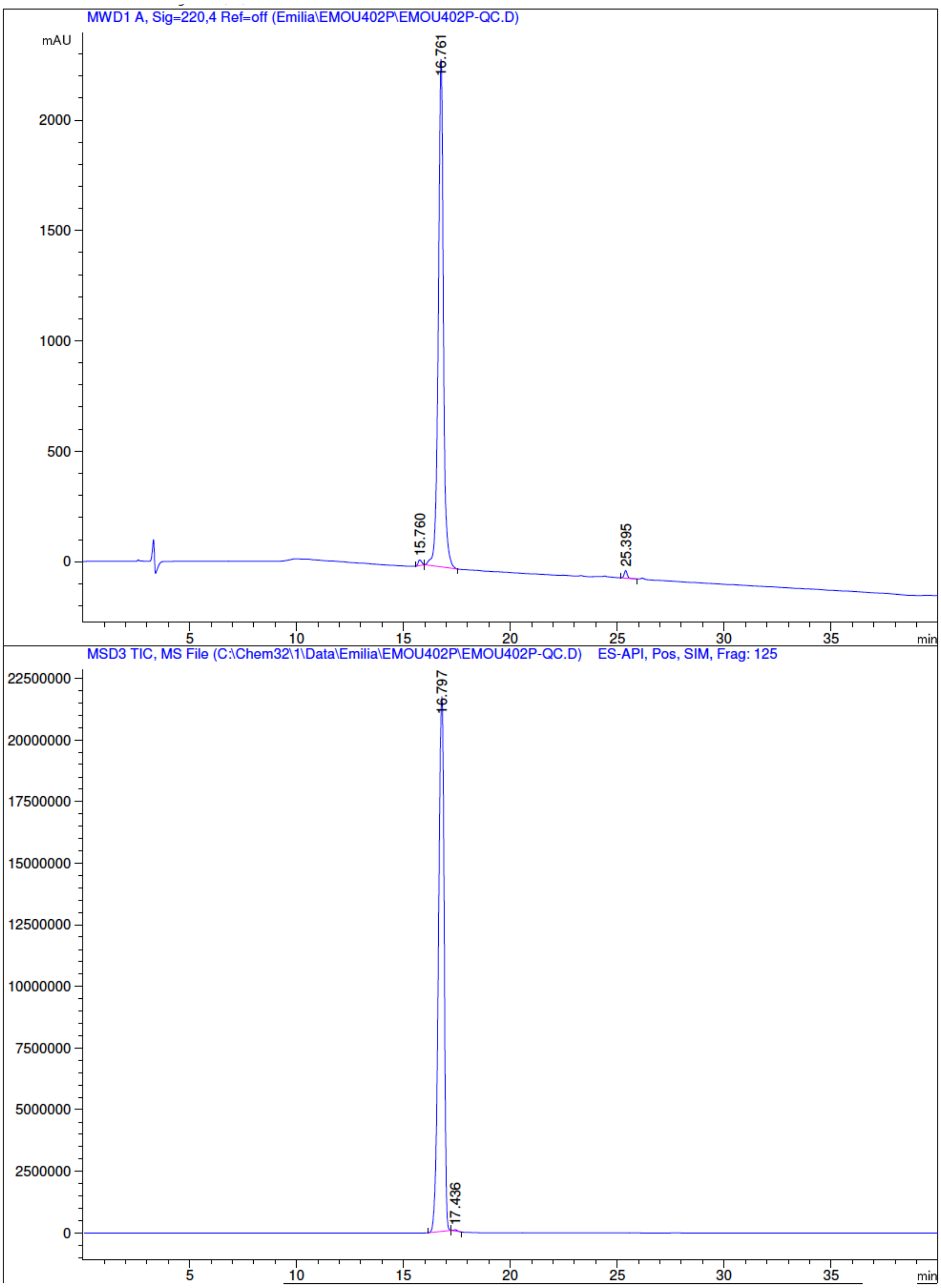

Peptide **37**; **8Aoc-8Aoc**-PAYD -NH_2, system A1

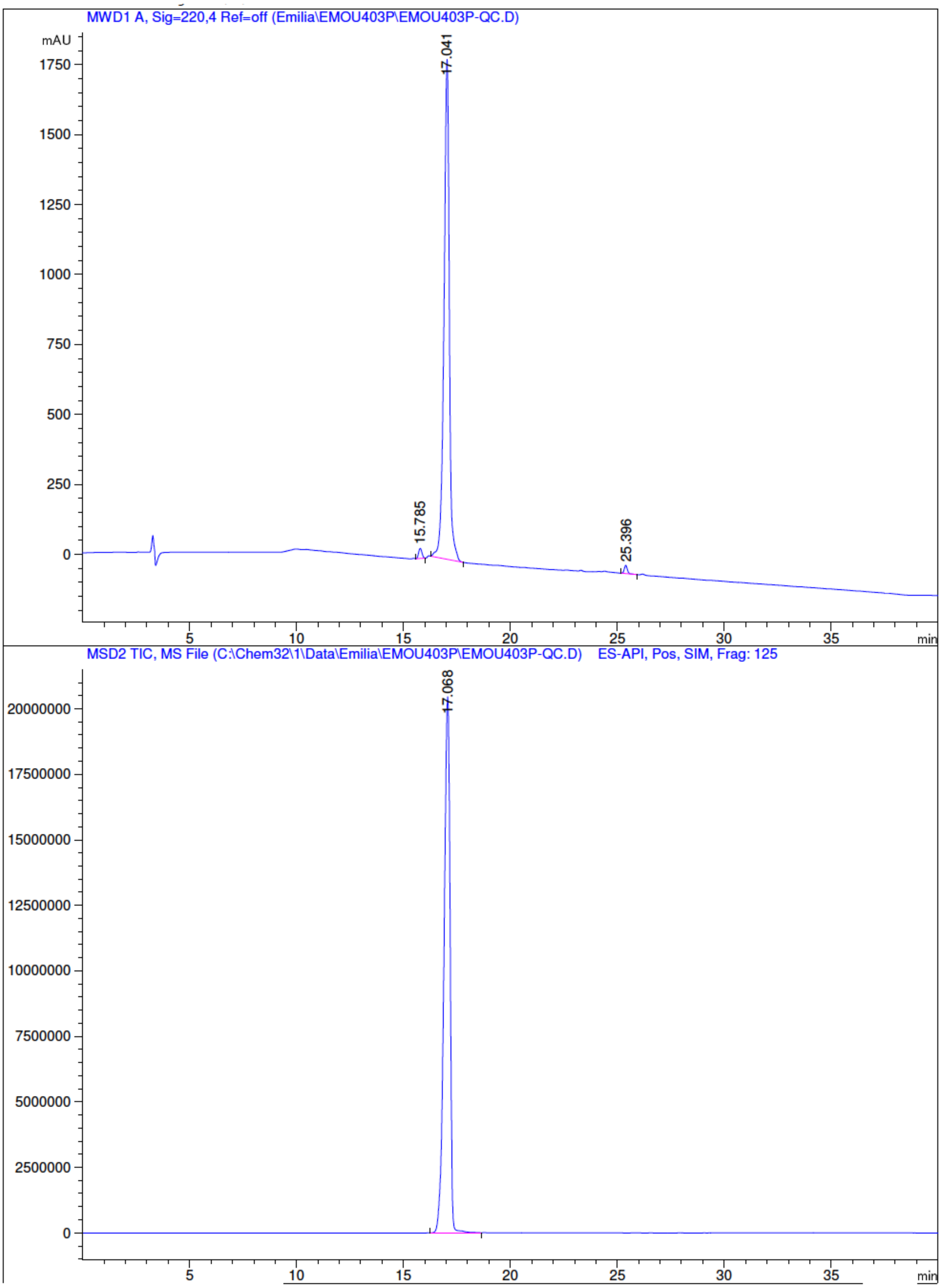

Peptide **38**; V-(**PEG)$_4$**-PAYD -NH$_2$, system A1

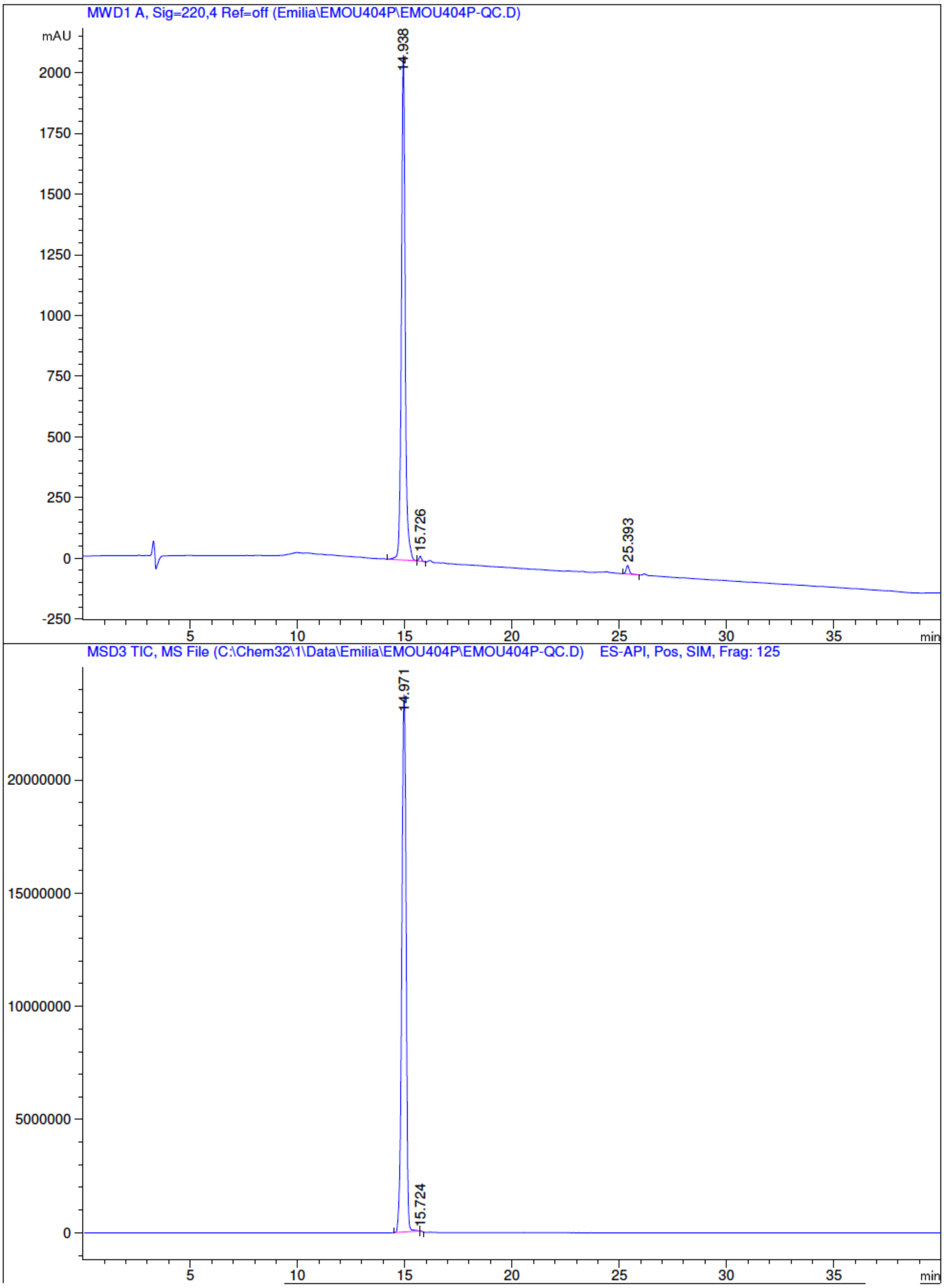

XVI. LC-MS traces of the sugar analogues intermediates

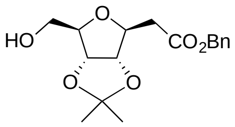

31, system A2

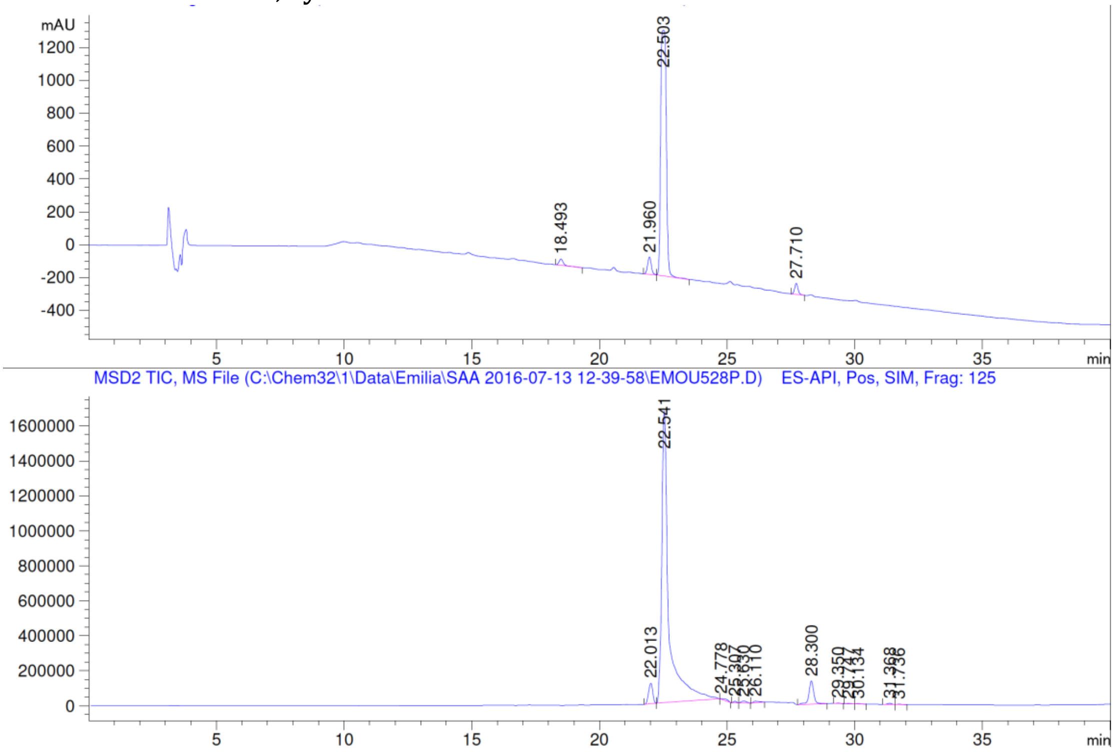

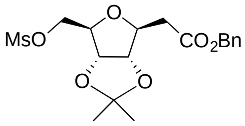

33, system A2

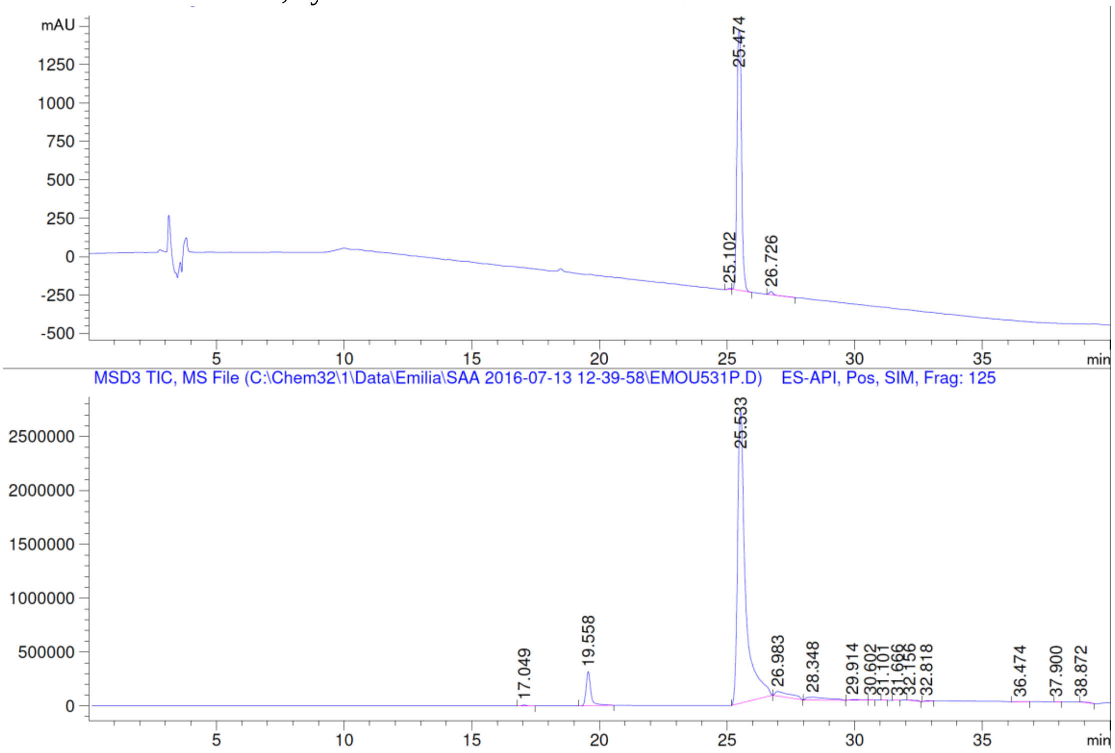

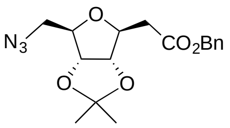

28, system A2

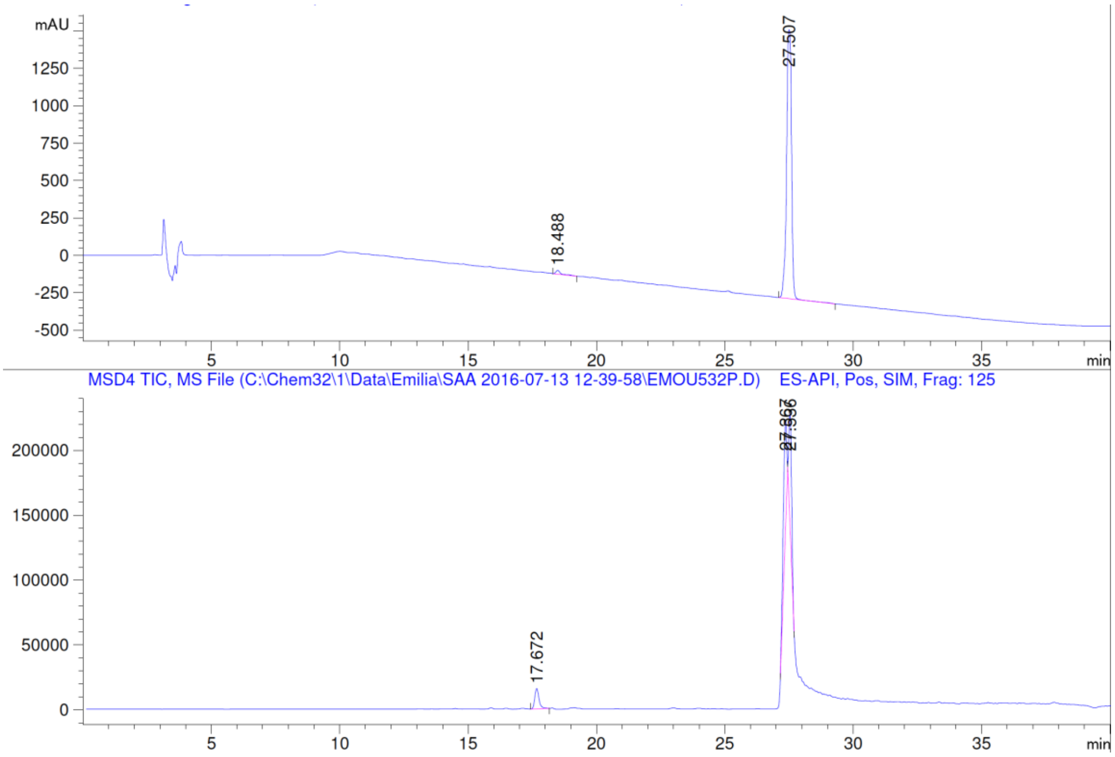

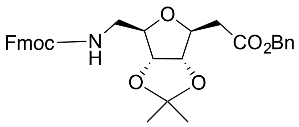

29, system A1

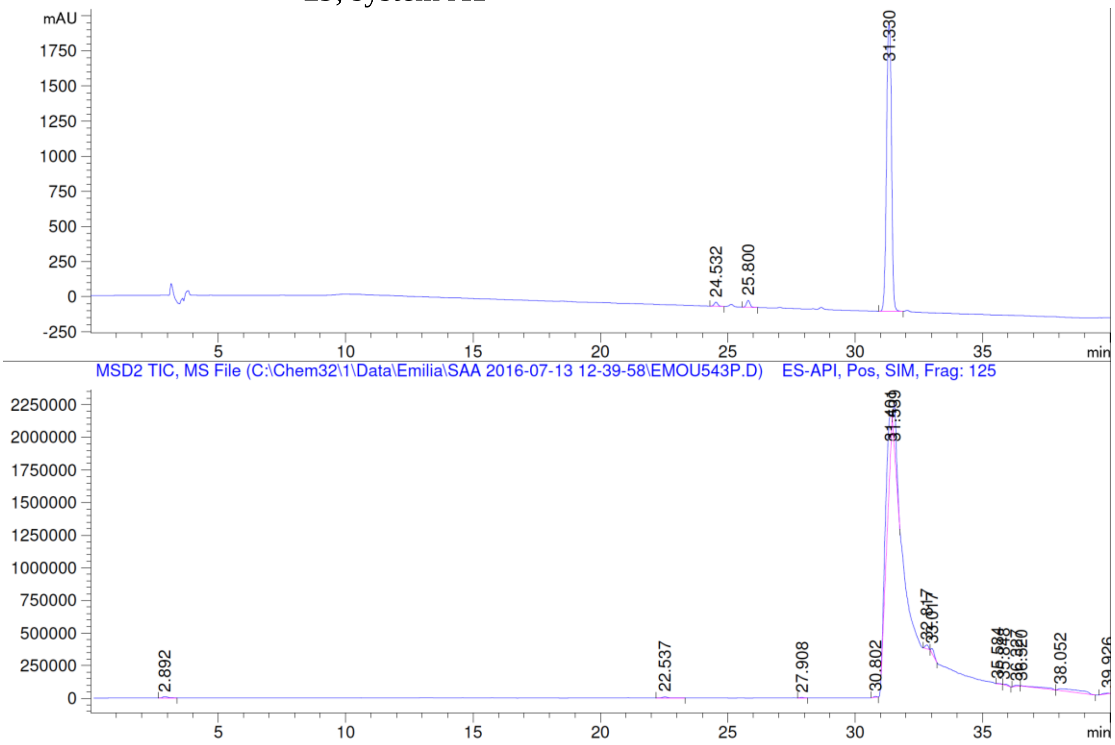

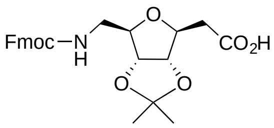

27, system A1

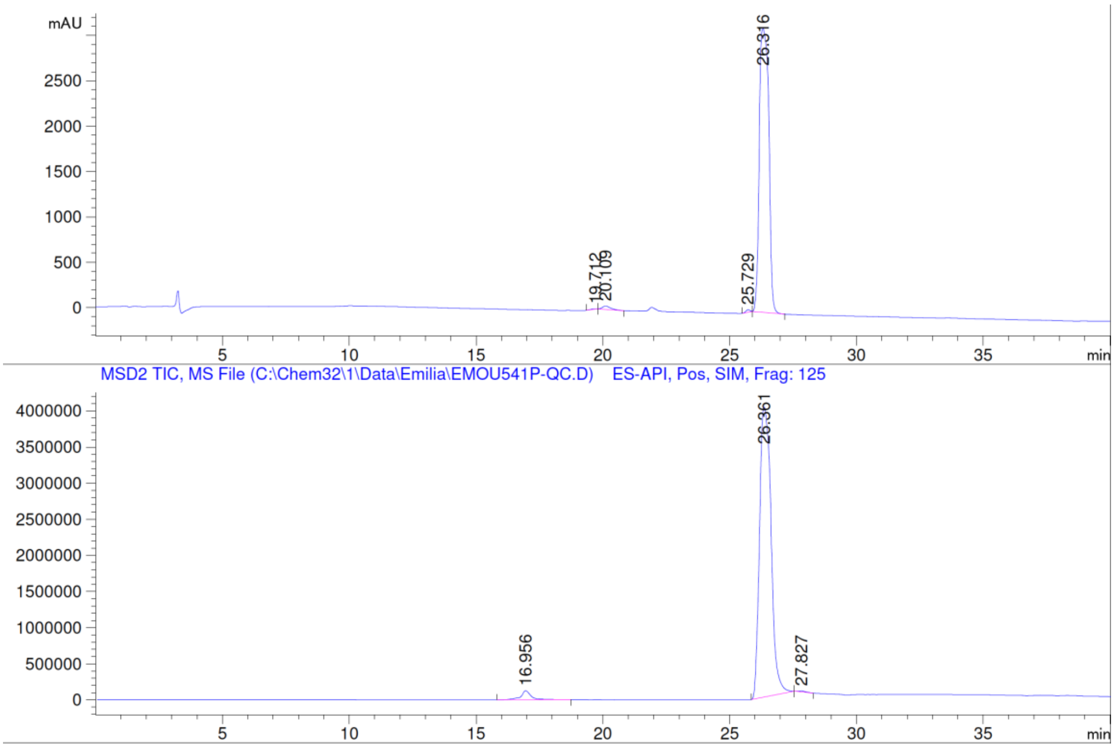

XVII. References

[1] G. R. Fulmer, A. J. M. Miller, N. H. Sherden, H. E. Gottlieb, A. Nudelman, B. M. Stoltz, J. E. Bercaw, K. I. Goldberg, *Organometallics* **2010**, *29*, 2176.

[2] J. Koehnke, A. Bent, W. E. Houssen, D. Zollman, F. Morawitz, S. Shirran, J. Vendome, A. F. Nneoyiegbe, L. Trembleau, C. H. Botting, M. C. M. Smith, M. Jaspars, J. H. Naismith, *Nat. Struct. Mol. Biol.* **2012**, *19*, 767.

[3] J. Koehnke, G. Mann, A. F. Bent, H. Ludewig, S. Shirran, C. Botting, T. Lebl, W. E. Houssen, M. Jaspars, J. H. Naismith, *Nat. Chem. Biol.* **2015**, *11*, 558.

[4] W. E. Houssen, A. F. Bent, A. R. McEwan, N. Pieiller, J. Tabudravu, J. Koehnke, G. Mann, R. I. Adaba, L. Thomas, U. W. Hawas, H. Liu, U. Schwarz-Linek, M. C. M. Smith, J. H. Naismith, M. Jaspars, *Angew. Chem. Int. Ed.* **2014**, *53*, 14171.

[5] R. M. Van Well, H. S. Overkleeft, M. Overhand, E. Vang Carstenen, G. A. van der Marel, J. H. van Boom, *Tetrahedron Lett.* **2000**, *41*, 9331.

[6] N. A. Ivanova, Z. R. Valiullina, O. V. Shitikova, M. S. Miftakhov, *Russ. Chem. Bull.* **2005**, *54*, 2698.

[7] Renate M. Van Well, L. Marinelli, K. Erkelens, Gijsbert A. van d. Marel, A. Lavecchia, Herman S. Overkleeft, Jacques H. v. Boom, H. Kessler, M. Overhand, *Eur. J. Org. Chem.* **2003**, *2003*, 2303.

[8] Y.-C. Hsieh, J.-L. Chir, W. Zou, H.-H. Wu, A.-T. Wu, *Carbohydr. Res.* **2009**, *344*, 1020.
